# The intriguing chemistry and biology of sulfur-containing natural products from marine microorganisms (1987–2020)

**DOI:** 10.1007/s42995-021-00101-2

**Published:** 2021-05-19

**Authors:** Yang Hai, Mei-Yan Wei, Chang-Yun Wang, Yu-Cheng Gu, Chang-Lun Shao

**Affiliations:** 1 Key Laboratory of Marine Drugs, School of Medicine and Pharmacy, The Ministry of Education of China, Ocean University of China, Qingdao, 266003 China; 2 Laboratory for Marine Drugs and Bioproducts, Pilot National Laboratory for Marine Science and Technology (Qingdao), Qingdao, 266237 China; 3 College of Food Science and Engineering, Ocean University of China, Qingdao, 266003 China; 4 Syngenta Jealott’s Hill International Research Centre, Bracknell, Berkshire RG42 6EY UK

**Keywords:** Sulfur-containing natural products, Marine microorganisms, Molecular diversity, Bioactivities, Marine drugs

## Abstract

Natural products derived from marine microorganisms have received great attention as a potential resource of new compound entities for drug discovery. The unique marine environment brings us a large group of sulfur-containing natural products with abundant biological functionality including antitumor, antibiotic, anti-inflammatory and antiviral activities. We reviewed all the 484 sulfur-containing natural products (non-sulfated) isolated from marine microorganisms, of which 59.9% are thioethers, 29.8% are thiazole/thiazoline-containing compounds and 10.3% are sulfoxides, sulfones, thioesters and many others. A selection of 133 compounds was further discussed on their structure–activity relationships, mechanisms of action, biosynthesis, and druggability. This is the first systematic review on sulfur-containing natural products from marine microorganisms conducted from January 1987, when the first one was reported, to December 2020.

**Supplementary Information:**

The online version contains supplementary material available at 10.1007/s42995-021-00101-2.

## Introduction

The ocean is the birthplace of life and occupies more than 70% of the earth's surface. Owing to the unique marine environment of hypoxia, high pressure, high salt and low temperature in which they are living, marine organisms have proven to be a rich source of structurally diverse and pharmacological active substances. Approximately 28,500 marine natural products (MNPs) had been identified by the end of 2018 (Carroll et al. 2019, 2020; Jimenez 2018). MNPs have a very high hit rate in biological activity screening (Gerwick and Moore 2012; Jimenez 2018). Prominently, marine microorganisms have taken the limelight as potential sources of biologically active natural products, and their potential will be explored continuously as promising new chemistry entities for drug development (Hou et al. 2015, 2019a; Liu et al. 2019b; Pettit et al. 1987).

The chemistry of marine natural sulfur compounds can be traced back to 1909 when Tyrian purple was discovered and considered to be produced by sulfur-containing precursors (Christophersen 1989; Friedländer 1909). Gliovictin is the first marine sulfur-containing MNP (non-sulfated) derived from microorganisms reported in 1987 (Shin and Fenical 1987).

**Fig. 1:**
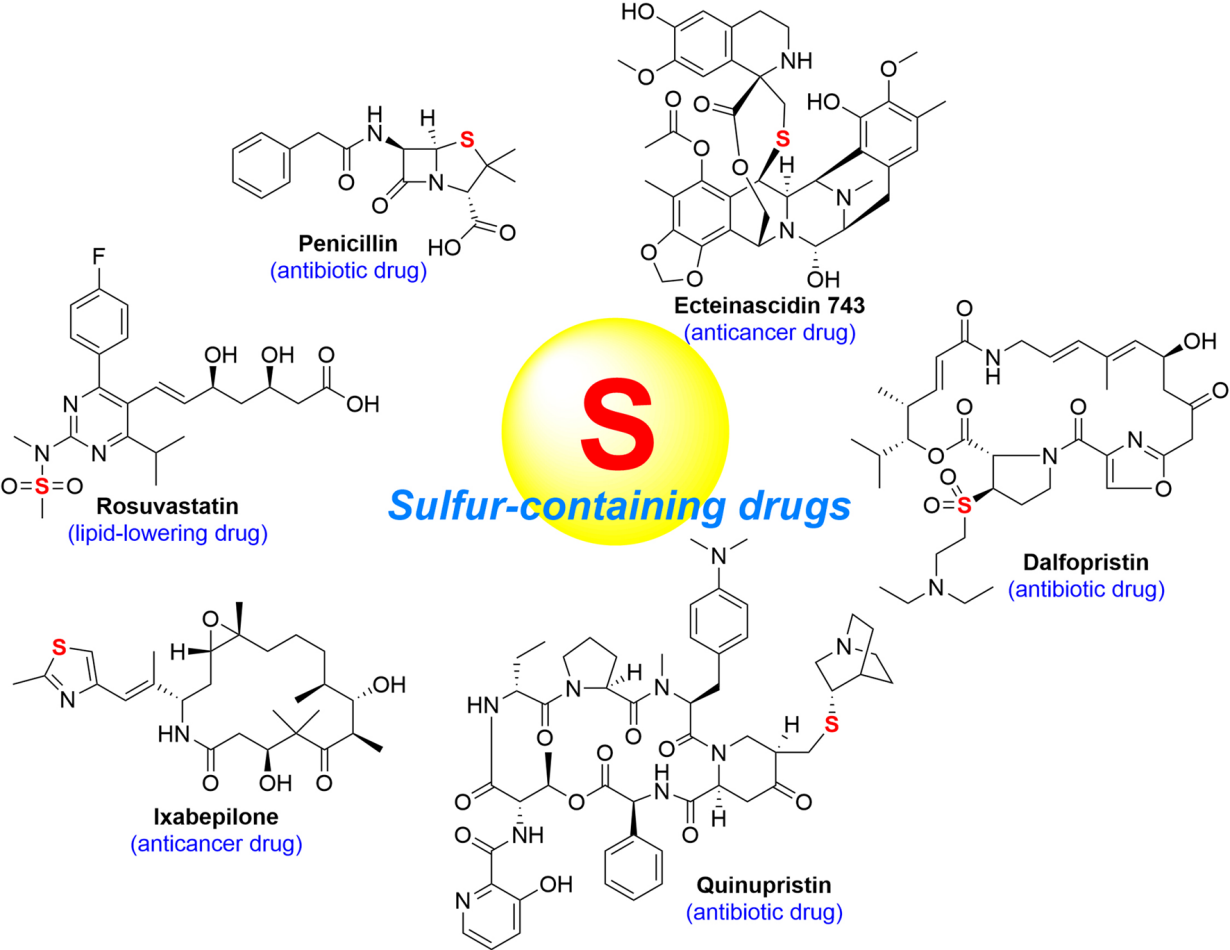
Representatives of sulfur-containing drugs

The influence of sulfur in the pharmaceutical industry is self-evident. It was reported that 41 sulfur-containing commercial drugs appear in the Top 200 Pharmaceuticals by Retail Sales in 2019 worldwide, it counts for 20.5% (McGrath et al. 2010). The well-known penicillin, ecteinascidin 743 (ET-743) and conotoxin belong to sulfur-containing clinical drugs developed from natural products (Fleming 1929). In addition, many sulfur-containing drugs are modified from natural products, for instance, ixabepilone and phthalascidin for cancer treatments, quinupristin and dalfopristin for bacteria-related infectious diseases, and rosuvastatin for hyperlipidemia. (Fig. 1).

There are few books and papers focused on marine microorganisms and sulfur-containing compounds derived from them. Timely revisions on new MNPs and their biological activities are important to update researchers on the fast progresses in these fields (Christophersen 1989; Christophersen and Anthoni 1986; Jiang and Guo 2011; Petkowski et al. 2018; Zhu et al. 2020a, b).

This review focuses on the comprehensive information from biological sources to pharmacological activities of all 484 sulfur-containing natural products (non-sulfated) of marine microorganisms reported from January 1987, when the first one was published, to December 2020. The compound isolation, structural elucidation, biological property evaluation, structure–activity relationship and mechanism of action will be discussed. In particular, the introduction of the sulfur atom in the field of biosynthesis and total synthesis and druggability are also highlighted.

## Thioethers

Thioethers are a class of compounds with the general formula R–S–R, and occupy a classic category among MNPs.

### Sulfides

#### Thiodioxopiperazines

Thiodioxopiperazines (TDPs) are a class of prominent dipeptides with a wide range of biological activities, including anticancer (Harms et al. 2015; Rodrigues et al. 2015; Yamada et al. 2004, 2002), antibacterial (Fukuda et al. 2015a, b; Li et al. 2006) and antiviral (Niu et al. 2017a, b) effects. The TDP ring confers increased structural rigidity, making TDPs attractive in pharmaceutical development. In particular, the presence of sulfur bridge plays an important role in biological activity (Feng et al. 2004; Takahashi et al. 1995a; Yamada et al. 2002, 2004).

Over the past three decades, more than 150 TDPs isolated from marine fungi have been reported. The first TDP, gliotoxin, a metabolite of the terrestrial fungus *Gliocladium fimbriatum* presented antibiotic, antiviral, immunosuppressive, anti-platelet aggregation and antitumor effects (Bell et al. 1958; Fridrichsons and McL Mathieson 1967; Johnson et al. 1943; Weindling 1932). *L*-phenylalanine and *L*-serine constitute the skeleton of gliotoxin.

Due to these intriguing properties, other genus of *Pseudallescheria*, *Neosartorya*, *Aspergillus, Dichotomomyces, Trichoderma* and *Penicillium* were studied which led to the isolation of ten congeners **1**–**10**. It was reported that 6-acetylmonodethiogliotoxin (**1**) and 6-acetylbisdethiobis (methylthio) gliotoxin **(2)** showed anti-inflammatory properties and inhibited TNF-α-induced NF-κB activity, while acetylgliotoxin G (**3**) displayed 20–30-fold increased cytotoxicity against HCT-116 cell line versus that of **2** (Harms et al. 2015; Rodrigues et al. 2015). Compared to potent cytotoxic reduced gliotoxin (**4**), 6-acetylbis (methylthio)gliotoxin (**5**) lacked any activity against HEK 293, HCT-116 and RKO cells (IC_50_ > 50 μmol/L), presumably since the thiol groups at C-3 and C-10a were methylated or the 6-OH was acetylated (Liang et al. 2014). Bis(dethio)-10a-methylthio-3a-deoxy-3,3a-didehydrogliotoxin (**6**) and 6-deoxy-5a,6-didehydrogliotoxin (**7**) exhibited strong and potent inhibition of the P388 cells. The presence of a hydroxy group at C-6 in **7** interfered with the histone methyltransferase (HMT) G9a inhibitory activity compared with other reported compounds (Sun et al. 2012). Dehydroxybisdethiobis(methylthio)gliotoxin (**8**) displayed moderate antibacterial effects against methicillin resistant *Staphylococcus aureus* (MRSA) with an MIC value of 31.2 μg/ml (Li et al. 2006). (Table 1).

**Table 1:** The gliotoxins origin, cytoxicity and other activities

Compound	Origin	Cytoxicity (IC_50_, cell)	Other activities
6-Acetylmonodethiogliotoxin (**1**)	*Dichotomomyces cejpii*	–	Anti-inflammatory; inhibition of NF-κB activity
6-Acetyl*bis*dethio*bis*(methylthio) gliotoxin **(2)**	*Dichotomomyces cejpii*	80.26 μmol/L (HCT-116 cells)	anti-inflammatory; inhibition of NF-κB activity
Acetylgliotoxin G (**3**)	*Dichotomomyces cejpii*	2.45 μmol/L (HCT-116 cells)	–
Reduced gliotoxin (**4**)	*Neosartorya pseudofischeri*	0.43 μmol/L (HCT-116 cells	–
6-Acetyl*bis*(methylthio)gliotoxin (**5**)	*Neosartorya pseudofischeri*	–	–
Bis(dethio)-10a-methylthio-3a-deoxy-3,3a-didehydrogliotoxin (**6**)	*Penicillium* sp.	3.4 μmol/L (P388 cells)	–
6-Deoxy-5a,6-didehydrogliotoxin (**7**)	*Penicillium* sp.	0.058 μmol/L (P388 cells)	Inhibition of (HMT) G9a activity
Dehydroxy*bis*dethio*bis*(methylthio)gliotoxin (**8**)	*Pseudallescheria* sp.	–	Antibacterial activity against MRSA
Dehydroxymethyl*bis*(dethio)*bis*(methylthio)gliotoxin **(9)**	*Trichoderma virens*	–	–
5a,6-Anhydro*bis*dethio*bis* (methylthio)gliotoxin (**10**)	*Dichotomomyces cejpii*	–	–

Geospallins A–C (**11**–**13**) were found to moderately inhibit angiotensin converting enzyme, which were obtained from *Geosmithia pallida* (Sun et al. 2018). Using a bioassay-guided isolation strategy, bioactive compounds **14**–**16** from *Dichotomomyces cejpii* were isolated and identified (Chen et al. 2017b; Zhen et al. 2016). Astonishingly, dichotocejpin A (**14**) displayed stronger inhibitory activity (IC_50_ = 138 µmol/L) against α-glucosidase than the positive control acarbose (IC_50_ = 463 µmol/L) (Supplementary Fig. S1).

A fermentation of the fungus *Penicillium janthinellum* HDN13-309 yielded six gliovirin-like compounds, penicisulfuranols A–F (**17**–**22**). Of which, compounds **17**–**19** were strongly cytotoxic to the HeLa and HL-60 cells with IC_50_ values ranging from 0.1 to 3.9 μmol/L whilst **20**–**22** were inactive. Compounds **20**–**22** can be considered as the methylated derivatives after sulfur bridge cleavage. The authors analyzed the fresh EA fractions using HPLC and concluded that they were not artificial products during the isolation process (Zhu et al. 2017). Additionally, compound **17** was a novel C-terminal inhibitor of Hsp90 targeting Hsp90 to exert the inhibitory effects of tumor cells (Dai et al. 2019). All activity evaluations confirmed that disulfide bonds were important structures for bioactivities (Dai et al. 2019; Zhu et al. 2017). Adametizine A (**23**) showed strong mortality on brine shrimp and moderate antibacterial action whilst adametizine B (**24**) only demonstrated weak antibacterial activity against *S. aureus.* Activity differences between **23** and known adametacorenols A–B proved that the Cl atom at C-7 enhanced the brine shrimp lethality and antimicrobial activity (Liu et al. 2015b). Subsequently, the analogs pretrichodermamides D–F (**25**–**27**) originated from *Penicillium* sp. did not show any cytotoxicity (Yurchenko et al. 2016). Extensive chemical investigations yielded peniciadametizines A (**28**) and B (**29**), which slightly inhibited the plant pathogenic fungus *Alternaria brassicae* (Liu et al. 2015c). DC1149B (**30**), iododithiobrevamide (**31**), DC1149R (**32**) and chlorotrithiobrevamide (**33**) were biosynthesized by *Trichoderma* sp. in the culture medium with added NaCl, NaBr, NaI and DMSO (Yamazaki et al. 2015a, b). The antimicrobial and antitumor effects of **30** and **32** have been reported (Nakano et al. 1990). Compound **33** possessing a rare trithio-bridge exhibited evidently reduced effects against HCT-15 cells and moderate cytotoxic effects against Jurkat cells (Yamazaki et al. 2015b) (Supplementary Fig. S2).

(+)-Gliocladins A (**34**) and B (**35**) with 3′-indolyl unit at C-3 were a type of moderate cytotoxic metabolites from the fungus *Gliocladium* sp. (Usami et al. 2004). Regioselective synthesis of **35** resolved the absolute configuration of *S*-methyl at C-15, exploiting a Friedel–Crafts-based strategy (Boyer and Movassaghi 2012). Luteoalbusins A (**36**) and B (**37**), isolated from *Acrostalagmus luteoalbus*, showed stronger cytotoxicity against SF-268, MCF-7, NCI-H460, and HepG-2 cells (IC_50_ = 0.23–1.31 μmol/L) than positive control cisplatin in vitro (IC_50_ = 2.45–4.76 μmol/L). Comparing the test results with other analogs (**36a** and **37a**), it can be deduced that the presence of the acetoxy group at C-17 may reduce cytotoxic activity (Adams et al. 2015; Wang et al. 2012a). Plectosphaeroic acids A–C (**38**–**40**), as the inhibitors of IDO in vitro, were isolated from *Plectosphaerella cucumerina* along with inactive T988 A. Therefore, their phenoxazinone moieties were recognized as a new IDO inhibitory pharmacophore (Carr et al. 2009). Compounds **39** and **40** had been synthesized by an enantioselective method applying the copper-mediated amination methods (Jabri and Overman 2013) (Supplementary Fig. S3).

Following the biological effects combined with an ^1^H NMR/ESIMS method, eutypellazines A–L (**41**–**52**) were isolated from the fungus *Eutypella* sp. MCCC 3A00281 (Niu et al. 2017a, b). All compounds displayed significant antiviral activities against HIV-1 virus with IC_50_ values ranged from 3.2 to 18.2 μmol/L and no cytotoxicity to normal human cell line 293 T (Niu et al. 2017a). Compound **50** showed the reactivation activities of latent HIV in vitro at 80 μmol/L. In continuing efforts to investigate *Eutypella* sp., eutypellazines N–S (**53**–**58**), six antibacterial congeners, were discovered. The authors inferred the series of compounds were formed by oxidation and nucleophilic attack of the intermediate, cyclo-*L*-Phe-*L*-Phe. Glutathione *S*-transferase mediated the introduction of *S*-methyl or sulfhydryl (Niu et al. 2017b). Phomazines A–C (**59**–**61**) were isolated from *Phoma* sp. and only **60** demonstrated weak cytotoxic effects against MGC-803 (Kong et al. 2014) (Supplementary Fig. S4).

A number of new disulfide-bridged diketopiperazine derivatives, brocazines A–G (**62**–**68**), were obtained from the cytotoxic extract of *Penicillium brocae* MA-231 (Meng et al. 2014). Compounds **62, 63** and **66**–**68** displayed potent to strong cytotoxicity against a range of human tumor cell lines (HTCLs) (Meng et al. 2016). The same sample also provided penicibrocazines A–E (**69**–**73**), which inhibited a range of bacteria at different levels with MIC values in the range of 0.25–32.0 µg/ml (Meng et al. 2015). A sample of *Exserohilum rostratum* produced rostratin A (**74**), with a *trans-*ring-fused system, and rostratins B–D (**75**–**77**), with a *cis-*ring-fused system, were structurally determined by the modified Mosher's methodology and NMR with low-temperature probes (Tan et al. 2004). All four metabolites indicated their potent or strong cytotoxicity to HCT-116 cells, with IC_50_ values of 8.5, 1.9, 0.76, and 16.5 µg/ml, respectively. With C–H bond activation as the key step, total synthesis of **74** had been achieved in 20 steps with an overall yield of 12.7% (Thesmar and Baudoin 2019). Cytotoxic cladosporins A (**78**) and B (**79**) were obtained from *Cladosporium* sp. by applying high-speed counter-current chromatography (Gu et al. 2015). Pseuboydones C (**80**) and D (**81**) were isolated from *Pseudallescheria boydii*. It is worth mentioning that **80** revealed potent cytotoxicity against Sf9 insect cells with an IC_50_ value of 0.7 μmol/L (Lan et al. 2016).

Ten new epipolythiodioxopiperazines (ETPs), amphiepicoccins A–J (**82**–**91**), were isolated from the extract of fungus *Epicoccum nigrum* HDN17-88. Compounds **82**, **84** and **87** exhibited moderate anti-HSV-2 activities with IC_50_ values of 70, 64 and 29 μmol/L, respectively; while **86** and **87** also existed inhibitory activity against *Bacillus subtilis* with MIC values of 13 and 25 μmol/L, respectively (Wang et al. 2020a). The investigation of *E. nigrum* SD-388 led to the isolation of six new thiodiketopiperazines **92–97**. Among them, 7-dehydroxyepicoccin H (**92**) and 7-hydroxyeutypellazine F (**93**) displayed moderate antibacterial activities against aquatic pathogens *Vibrio vulnificus*, *V. alginolyticus* and *Edwardsiella tarda*, with MIC values ranging from 4.0 to 8.0 μg/ml. 7′-demethoxyrostratin C (**97**) showed potent cytotoxic activity against Huh7.5 cells with an IC_50_ value of 9.52 μmol/L, comparable to that of the positive control of sorafenib (8.2 µmol/L) (Chi et al. 2020a, b). Penispirozines A–D (**98–101**) from *P. janthinellum* possessed interesting spirocyclic skeletons. Meanwhile, compounds **100** and **101** increased the expression of the two relevant phase II detoxifying enzymes SOD2 and HO-1 at 10 μmol/L (Zhu et al. 2020a, b) (Supplementary Fig. S5).

Based on a screening system, a strain of *Graphium* sp. isolated from marine sediment yielded a great number of compounds, graphiumins A–J (**102–111**). Most of these metabolites exhibited the selective inhibition of yellow pigment production in MRSA without influencing the growth of pathogenic bacteria (Fukuda et al. 2015a, b). Alternarosin A (**112**) possessing slight antibacterial activity was obtained from *Alternaria raphanin* (Wang et al. 2009). Deoxyapoaranotin (**113**) was isolated from *Aspergillus* sp. and found to have direct cytotoxic and apoptosis-inducing effects towards HCT-116 cells (Choi et al. 2011) (Supplementary Fig. S6).

As the name implies, monocyclic compounds have only one ring system in the skeleton, including compounds **114**–**140**. Gliovictin **(114)** from *Asteromyces cruciatus* is the first isolated marine sulfur-containing natural product (non-sulfated) derived from microorganisms in 1987 (Shin and Fenical 1987). The strains *Fusarium chlamydosporum, Penicillium crustosum, Pleosporales* sp. are the sources of Sch54794 (**115**), 54796 (**116**), fusaperazines A, B, F (11**7, 118, 119**) and (Z)-6-benzylidene-3-hydroxymethyl-1,4-dimethyl-3-methylsulfanylpiperazine-2,5-dione (**120**) (Liu et al. 2019a; Prachyawarakorn et al. 2008; Usami et al. 2002). Meanwhile, **126** and **129** showed strong cytotoxic activities against Hep2 and K562 cells, respectively (Li et al. 2008; Liu et al. 2019a; Prachyawarakorn et al. 2008; Usami et al. 2002). Other structurally similar metabolites, bilains A–C (**121**–**123**), were produced by *Penicillium bilaii* (Capon et al. 2007) and *Sarocladium kiliense*, the culture that yielded saroclazines A (**124**) and B (**125**). The free amide moiety appeared for the first time in sulfur-containing aromatic DKPs. Despite a few differences in structures between them, only **125** had a strong cytotoxic effect (Li et al. 2018a) (Supplementary Fig. S7).

Two pairs of enantiomers, (±)-acrozines A (**126** and **127**) and B (**128** and **129**), were acquired from *A. luteoalbus.* Surprisingly, they had a unique N-OMe group in their indolediketopiperazine scaffold, which had been proved as natural products, not artifacts. Compound **129** showed moderate activity against the plant pathogen *Fusarium solani*. After chiral resolution, six samples including two racemates and four pure compounds were tested for the inhibitory activity toward acetylcholinesterase (AChE) in vitro. On the whole, acrozine A-related samples showed stronger anti-AChE activities than acrozine B. Among them, **126** was the most active AChE inhibitor with an IC_50_ value of 2.3 μmol/L. Furthermore, it had been proved that C-3 assigned as *R* configuration may enhance AChE activity. These results suggested that even enantiomer or epimer can possess different bioactivities (Cao et al. 2019b). Three new DKPs consisting of a pair of bridged epimonothiodiketopiperazine diastereomers **130**–**132** were identified from *Pseudallescheria ellipsoidea* F42-3 (Liu et al. 2015a; Wang et al. 2016). *Streptomyces olivaceus* yielded two oxazole/thiazole derivatives, tetroazolemycins A (**133**) and B (**134**) with metal ion-binding affinity for the metal ions Fe^3+^, Cu^2+^ and Zn^2+^, and the Zn^2+^ complexes showed weak activity against pathogenic bacteria *Klebsiella pneumonia* (Liu et al. 2013). Further investigation of the antitumor constituents of *Aspergillus fumigatus* and *Trichoderma virens* led to the isolation of two gliotoxin analogues **135** and **136**. However, both of these did not exhibit cytotoxic activity, which also confirmed that sulfide bridge in the gliotoxin family might be an important pharmacophore for their cytotoxic activity (Shi et al. 2018b; Zhao et al. 2009). Glioperazine (**137**) was obtained from *Gliocladium* sp. along with gliocladins A and B, which also displayed modest cytotoxicity against P388 cells (Usami et al. 2004). Maremycins A–B (**138**–**139**) and cyclo (*L*-Pro-*D*-Met) **(140)** were metabolites from *Streptomyces* sp. and *Pseudomonas aeruginosa*, respectively (Balk-Bindseil et al. 1995; Jayatilake et al. 1996) (Supplementary Fig. S8).

Spirobrocazines A (**141**) and B (**142**) were isolated from *P. brocae*, whereas only **141** had weak antibacterial activity against three pathogenic bacteria (Meng et al. 2016). Another strain *Penicillium* sp. yielded citriperazines A–C (**143**–**145**), and they did not show cytotoxic activity against human prostate cancer cells (Yurchenko et al. 2019). Spirogliotoxin (**146**) was a spiro compound in gliotoxin family isolated from the fungus *A. fumigatus* (Wang et al. 2012c) (Supplementary Fig. S9).

The investigation of cytotoxic metabolites from *Leptosphaeria* sp. led to the isolation of leptosins **147**–**169**. Their absolute configuration had been elucidated by spectral data and chemical strategies. All these metabolites exhibited significant cytotoxic activity against P388 cells in vitro (Takahashi et al. 1994a, b,1995a, b, 2004; Yamada et al. 2002). In the same series, compounds **147**–**149** reached the strongest nanomole-level activity at ED_50_ values of 1.75–2.4 ng/ml (Takahashi et al. 1994a). Compounds **162**–**169** were dimer compounds, where one monomer contains a sulfur bridge resulting in the cytotoxicity being greatly reduced. Monomer compounds **150**–**152** were also not as active as **147**–**149** (Takahashi et al. 1995a). These results confirmed that the dimer structure and the number of sulfur bridges were conducive to cytotoxic activity. In addition, **162** was proved to exhibit strong selective cytotoxic effects against 39 HTCLs, and to inhibit two protein kinases, PTK and CaMKIII, and human topoisomerase II. Intriguingly, using the COMPARE program, it showed the possibility that the mode of action for **162** might be different from that shown by any other anticancer drug developed (Yamada et al. 2002). Chetracins E (**170**) and F (**171**) were produced by *A. luteoalbus* and exhibited strong cytotoxicity against five cancer lines and could function as Hsp90 C-terminal inhibitors (Takahashi et al. 1994a; Yu et al. 2018) (Table 2).

**Table 2:** Cytotoxity of leptosins against P388 cells (unit: μg/ml)

Compound	ED_50_	Compound	ED_50_
Leptosin A **(147)**	1.85 × 10^–3^	Leptosin K **(159)**	3.80 × 10^–3^
Leptosin B **(148)**	2.40 × 10^–3^	Leptosin K_1_ **(160)**	2.20 × 10^–3^
Leptosin C **(149)**	1.75 × 10^–3^	Leptosin K_2_ **(161)**	2.10 × 10^–3^
Leptosin D **(150)**	8.60 × 10^–2^	Leptosin M **(162)**	1.05
Leptosin E **(151)**	4.60 × 10^–2^	Leptosin M_1_ **(163)**	1.40
Leptosin F **(152)**	5.60 × 10^–2^	Leptosin N **(164)**	0.18
Leptosin G **(153)**	4.60 × 10^–3^	Leptosin N_1_ **(165)**	0.19
Leptosin G_1_ **(154)**	4.30 × 10^–3^	Leptosin O **(166)**	1.10
Leptosin G_2_ **(155)**	4.40 × 10^–3^	Leptosin P **(167)**	0.10
Leptosin H **(156)**	3.00 × 10^–3^	Leptosin Q **(168)**	14.80
Leptosin I **(157)**	1.13	Leptosin R **(169)**	15.20
Leptosin J **(158)**	1.25		

*Penicillium* sp. produced two dimers, 11,11-dideoxyverticillin A (**172**) and 11-deoxyverticillin A (**173**), both of which displayed potent cytotoxicity against the HCT-116 cell line in vitro with IC_50_ values in the low nmol/L range (Son et al. 1999). In subsequent research, **173** was found to induce autophagy, apoptosis and necrosis of tumor cells (Zhang et al. 2014). Compound **172** blocked tumor cells in G_1_ phase, and also had tyrosine kinase and neovascularization inhibitory effects (Chen et al. 2005). With the probable biogenetic synthesis and total synthesis of **172** having been resolved, it can be said that the potential to become a drug lead compound is huge (Kim et al. 2009). All four compounds from *Chaetomium cristatum* contained cristazine (**174**), which demonstrated the significant antioxidant activity of scavenging DPPH free radical at the same level as vitamin C. It also displayed potent cytotoxic activity against HeLa cells (Yun et al. 2016). Studies on its anti-proliferative and anticancer mechanisms revealed that **174** induced Type I death receptor apoptosis and G_1_/S cell cycle arrest in A431 cells (Jo et al. 2019) (Supplementary Fig. S10).

#### Thiophenes

The strain *Streptomyces* sp. provided four novel compounds, thioquinomycins A–D (**175**–**178**), that were used as inhibitors of PKCα and ROCK2 protein kinases. Additionally, they all exhibited weak cytotoxicity (Zhang et al. 2018a). Seriniquinone (**179**), an anticancer agent isolated from bacterium *Serinicoccus* sp., showed potent and selective cytotoxicity against melanoma cancer cells. Mechanism research found that **179** declined cell proliferation by autophagocytosis and induced cell death through the caspase-9 apoptotic pathway. Meanwhile, **179** was the first small molecular targeting dermcidin, a significant anticancer protein (Trzoss et al. 2014). Subsequent structure–activity relationships confirmed the important role of the thiophene ring in antitumor effect and designed and confirmed a carbamate derivative with potential for prodrug development (Hammons et al. 2019). Chromogenic ketones are a wide range of compounds with potential application value, but the natural products containing dihydrothiophene-condensed chromone skeleton were still rarely reported. When the medium condition was changed to PDB medium, the strain *Aspergillus terreus* produced an unreported compound, 8-hydroxy-2-[1-hydroxyethyl]-5,7-dimethoxynaphtho[2,3-b]thiophene-4,9-dione (**180**) (Deng et al. 2013).

The investigation of *Penicillium oxalicum* identified oxalicumones A (**181**), B (**182**), D (**183**) and E (**184**), which exhibited cytotoxic activity against several cancer cell lines included H1975, U937, K562, BGC823, MOLT-4, MCF-7, HL60 and Huh-7 (IC_50_ = 9.8–18.0 μmol/L). Through structural modification, structure–activity relationships could be inferred that the dihydrothiophene ring and methoxyl groups at C-16 and C-17 had a key role in the cytotoxicity of **181**, while the hydroxyl groups at C-1, C-11 and C-13 reduced the activity. Moreover, the configuration of C-6 had a significant effect on the cytotoxic activity of these compounds (Bao et al. 2014; Sun et al. 2013).

Improved HPLC–UV–MS technology combined with the experimental design and chemometric analysis guided the discovery of a class of macrocyclic polyketides from *Penicillium* sp., including cyclothiocurvularins A (**185**) and B (**186**), cyclothiocurvularin methyl ester (**187**), cyclosulfoxicurvularin (**188**) and cyclosulfoxicurvularin methyl ester (**189**). Among them, sulfoxide-containing cyclosulfoxicurvularins possessed more complex structures. *L*-cysteine was confirmed to be the precursor of the mercaptolactate moiety in cyclothiocurvularins by using feeding experiments with [U–^13^C_3_^15^N]-*L*-cysteine. In addition, the spontaneous formation of cyclothiocurvularins from mercaptopyruvate and 10,11-dehydrocurvularin clarified that biosynthesis of cyclothiocurvularins may be a detoxification process for the strain. Other metabolites obtained from *Streptomyces* sp. were 3-acetylamino-*N*-2-thienyl-propanamide (**190**) (Ye et al. 2017) and 2,5-bis(5-tertbutyl-2-benzoxazolyl)thiophene (**191**) (Cao et al. 2019a). The latter consisted of a benzoxazolyl structure and showed weak antibacterial activity against *Enterococcus faecalis* (Supplementary Fig. S11).

### Polyketides

The potent cytotoxic curvularin derivatives, sumalarins A–C (**192**–**194**), were identified from organic extracts of *Penicillium sumatrense*. Structure–activity relationships of sumalarins indicated the sulfur atom at C-11 or the double bond at C-10 increased the cytotoxic activity significantly (Meng et al. 2013). From the perspective of biotransformation, 3-mercaptolactate was a metabolite of cysteine in microorganisms. Then, **194** was the product of 3-mercaptolactate and cyclohexenone formed by condensation reaction (Adelin et al. 2012).

Pandangolides and thiocladospolides were obtained from *Cladosporium herbarum* and *Cladosporium cladosporioides*, respectively (Jadulco et al. 2001; Smith et al. 2000; Zhang et al. 2019a). In the process of separating thiocladospolides A–D (**195**–**198**), the structure of pandangolide 3 (**200**) that had been reported was revised by NMR and ECD, and the sulfur side chain was reassigned from the C-3 to C-2 position. By analogy, the structures of pandangolides 2 (**199**) and 4 (**201**) also need to be reconsidered and revised, unfortunately there is no relevant report yet. Similar to cyclothiocurvularins, based on the structural characteristics of pandangolides and thiocladospolides, they were also considered the metabolites during the detoxification process. In addition, **195**–**198** and **200** showed strong antimicrobial activities against several pathogenic bacterium strains (Zhang et al. 2019a). Chemical investigation of *Cladosporium oxysporum* obtained thiocladospolides F–J (**202**–**206**), while they displayed moderate or weak antimicrobial activities (Wang et al. 2020b). Thiocladospolides F′ (**207**) and G′ (**208**) from *C. cladosporioides* displayed moderate activities against pathogenic bacteria *E. tarda*, *Vibrio anguillarum* and *Helminthosporium maydis* with MIC values ranging from 2.0 to 8.0 μg/ml (Zhang et al. 2020). The introduction of neomycin resistance into *Penicillium purpurogenum* led to the isolation of a novel cyclopentachromone sulfide chromosulfine (**209**) with weak cytotoxicity. From the analysis of structural characteristics, 3-mercaptolactate was also involved in biosynthesis (Yi et al. 2016). ( −)-Homoseongomycin (**210**) was a metabolite bearing a benzo[b]fluorene core produced by the detoxification pathway of bacterium *Salinispora pacifica* (Woo et al. 2013) (Supplementary Fig. S12).

This strain *Streptomyce*s sp. produced a series of pyranonaphthoquinone dimers linked by a sulfur bridge including compounds **211**–**213** (Che et al. 2016). Although (−)-BE-52440A (**213**) had been obtained through chemical synthesis before, it was discovered as a natural product for the first time (Tatsuta et al. 2007). Compound **213** showed strong cytotoxic effects on NB4 and HL-60 cells, and naquihexcin A (**211**) exhibited a certain inhibitory effect on adriamycin-resistant MCF-7 cancer cell line with an IC_50_ value of 16.1 μmol/L, indicating that the hexuronic acid fragment may have important significance in improving selectivity for tumor cells (Che et al. 2016). Two strong antibacterial agents, kendomycins C (**214**) and D (**215**) were extracted from actinomycete *Verrucosispora* sp. Unlike **214** with moderate cytotoxic activity, **215** demonstrated only weak activity. However, their cytotoxicity lacked the selectivity between normal cells and cancer cells. The presence of *S*-methyl appeared to have little effect on antibacterial and cytotoxic activity (Zhang et al. 2019b).

Abyssomicins possessed mostly a four-membered or five-membered ring system as well as spirotetronate of 19 carbon atoms. Chemical semi-synthetic method found that members can be obtained by Michael addition reaction. Abyssomicin J (**216**) discovered from *Verrucosispora* sp. was the first compound with a sulfur-containing dimer structure, which showed the potential to be developed as an anti-tuberculosis prodrug. The experiment verified that **216** could spontaneously transform into *atrop-*abyssomicin C to exert its anti-tuberculosis activity at a cellular level and revealed that it can overcome the instability of atrop-abyssomicin C (Wang et al. 2013). Another two analogues, neoabyssomicins F (**217**) and G (**218**) were separated subsequently from *Streptomyces koyangensis*. They displayed weak antiviral activity and antibacterial activity against MRSA (Huang et al. 2018). Urdamycinones E (**219**) and G (**220**) were the C-glycosylated benz[α]anthraquinone derivatives extracted from *Streptomyces* sp., and co-isolated urdamycin E was regarded as the common precursor. Owing to the activity-guided separation strategy, these compounds had abundant activities, including anti-tuberculosis, antimalarial against *Plasmodium falciparum* and cytotoxicity. Among all the obtained compounds, **219** showed the strongest activity in the above aspects, indicating the presence of the *S*-methyl and glycosidic moieties were conducive to activity (Supong et al. 2012). Algal sinapic acid induced a cultured *Phaeobacter inhibens* strain to produce the novel compound roseochelin B (**221**). The characteristic of iron binding and algicidal activity had been investigated. Additionally, the biosynthesis of **221** was proposed to involve the nonenzymatic and enzymatic conversion (Wang and Seyedsayamdost 2017) (Supplementary Fig. S13).

#### Peptides

Quinomycin A (echinomycin), a prominent target molecule for the development of anti-tumor drugs, inhibited hypoxia-inducible factor-1 (HIF-1) DNA binding (Kong et al. 2005). Chemical investigation of *Streptomyces* sp. obtained its analog, quinomycin G (**222**), which exhibited not only moderate antibacterial activities against drug-resistant/sensitive strains but also excellent antitumor activities. However, its bioactivities were lower than echinomycin (Zhen et al. 2015). With the assistance of peptidogenomics and molecular networking constructed from 35 *Salinispora* strains, another analogue, retimycin A (**223**) was found from *Salinispora arenicola* (Duncan et al. 2015). *Moorea producens* yielded a new lipopeptide, precarriebowmide (**224**) (Mevers et al. 2013) and two congeners, carriebowmide (**225**) and carriebowmide sulfone (**226**). Compounds **225** and **226** were first reported from *Lyngbya polychroa* (Gunasekera et al. 2008) and *Lyngbya majuscula* (Jiménez et al. 2009), respectively. Generally, methione sulfoxide was considered as the artificial product formed by the oxidation of methionine residue. The verification experiment found that the sulfur in methionine was easily oxidized. Hence, **224** was the true natural product, while **225** and **226** were only artificial products. In addition, **225** actually represented a mixture of two diastereomers due to the racemic sulfoxide group (Mevers et al. 2013). Oryzamides C–E (**227**–**229**) were isolated from the fungus *Nigrospora oryzae*. Similar to the aforementioned rule, **228** and **229** were also a pair of epimers, and both originated from **227**. Unfortunately, in the antibacterial, antiparasitic and cytotoxic tests, no activity was observed on **227** (Ding et al. 2016). A cyclohexadepsipeptide, arenamide C (**230**) was obtained from actinomycete *S. arenicola*. Co-isolated arenamides A and B were cytotoxic NFκB inhibitors, unfortunately, no activity was mentioned in **230** (Asolkar et al. 2009). Using the strategy of heterologous expression in *Streptomyces* sp. strains, neothioviridamide (**231**) with strong cytotoxicity was discovered. However, the absolute configuration of most amino acid residues were not determined (Kawahara et al. 2018). Verrucosamide (**232**) displayed moderate cytotoxicity and selectivity in the NCI 60 cell from *Verrucosispora* sp. (Nair et al. 2020) (Supplementary Fig. S14).

#### Alkaloids

Five *N*-methylsuccinimide derivatives, violaceimides A–E (**233**–**237**) were isolated from *Aspergillus violaceus*. Biosynthetic pathways involving methylsuccinic acid, cysteine and 3-mercaptolactate were proposed. Compounds **233, 234** and **237** displayed selective cytotoxicity against tumor cells, but no toxic effects on normal cells. From the results, the structure–activity relationships suggested that the presence of a mercaptoacetic unit reduced cytotoxicity (**235**, **236** vs. **233**, **234**), and at least one sulfur atom was necessary for cytotoxic activity (**233**, **234** vs*.* versimide) (Yin et al. 2018). A collection of *Streptomyces* sp. yielded bagremycins C (**238**) and F (**239**). It was found that **238** had cytotoxicity against glioma cells, induced apoptosis in a dose- and time-dependent fashion, and arrested the cell cycle at the *G*_0_/*G*_1_ phase. Just a small difference in structure, **239** had only weak antibacterial ability (Chen et al. 2017a; Zhang et al. 2018b). A cyslabdan-like antibacterial compound C_25_H_41_NO_5_S (**240)**, possessing β-lactamase inhibitory capability of Gram-negative pathogens and MRSA, was discovered from *Streptomyces* sp. Additionally, it enhanced the activity of third-generation cephalosporins and meropenem (Shanthi et al. 2015). The investigation of the *Streptomyces* sp. yielded two novel thioether compounds, cyanosporaside F (**241**) and heronamycin A (**242**) (Lane et al. 2013; Raju et al. 2012). Compound **242** exhibited modest antimicrobial activity against *B. subtilis*. Dermacozine J (**243**) possessed free radical scavenging activity with an IC_50_ value of 19.6 μmol/L from *Dermacoccus abyssi* (Wagner et al. 2014) (Supplementary Fig. S15).

*Bacillus* sp. produced a class of amicoumacin derivatives, including a pair of diastereoisomers, bacillcoumacins E (**244**) and F (**245**). These polyketide synthase-nonribosomal peptide synthetase (PKS-NRPS) hybrids displayed weak inhibitory activities. Additionally, co-isolated AI-77-F without *S*-methyl potently inhibited the NO production induced by lipopolysaccharide (Bai et al. 2014). *Streptomyces* sp. was the source of cysrabelomycin (**246**) that exhibited moderate cytotoxicities and antibacterial activities against *S. aureus* and *Candida albicans* (Zhou et al. 2019). Compared with its precursor, gliomastin B (**247**) isolated from *Gliomastix* sp. had no cytotoxic activity and anti-tuberculosis activity (Elnaggar et al. 2017). Benzoxacystol (**248**) derived from *Streptomyces griseus* was an inhibitor of glycogen synthase kinase 3β. In addition, it possessed a 1,4-benzoxazine skeleton and slight anti-proliferative activity in vitro (Nachtigall et al. 2011). Under the guidance of GC–MS, a series of pyrazines were discovered from *Loktanella* sp. including 2,5-dimethyl-3-(methylsulfanyl) pyrazine (**249**), which was previously reported as a flavoring agent and first reported from a natural source (Dickschat et al. 2005). 1-Methyl-4-methylthio-β-carboline (**250**) and 4-(1H-indol-3-yl-sulfanyl) phenol (**251**) had been discovered and identified in succession (Lorig-Roach et al. 2017; Nair et al. 2016). Chemical investigation of several actinomycete strains also led to the isolation of compounds **252**–**257**. Among them, streptopertusacin A (**253**) demonstrated moderate antibacterial effects against MRSA with an MIC value of 40 μg/ml (Bu et al. 2014; Fu and MacMillan 2015a; Kyeremeh et al. 2014; Newton et al. 2008; Zhang et al. 2017b). Monacycliones H (**258**) and I (**259**) were isolated from *Streptomyces* sp. and **259** showed moderate cytotoxicity against HL-60 cells with an IC_50_ value of 7.6 μmol/L (Chang et al. 2020). Androsamide (**260**) was a potential inhibitor against colorectal cancer motility from *Nocardiopsis* sp. and strongly suppressed the motility of Caco2 cells caused by epithelial-mesenchymal transition (Lee et al. 2020). Halosmysin A (**261**) was isolated from *Halosphaeriaceae* sp. Additionally, it exhibited bioactivity against P388, HL-60 and L1210 cells with IC_50_ values ranging from 2.2 to 11.7 µmol/L (Yamada et al. 2020) (Supplementary Fig. S16).

#### Disulfides

Thiomarinols A–G (**262**–**268**), excellent antimicrobial agents, were obtained from *Alteromonas rava* sp. (Shiozawa et al. 1994, 1995, 1997; Shiozawa and Takahashi 1994). Mutant strain *Pseudoalteromonas* sp. yielded a group of thiomarinol derivatives (**269**–**276**), polyketide/fatty acid hybrids, which were also enol-ketone tautomers or epimers (Gao et al. 2017) (Supplementary Fig. S17).

The stereostructure of thiocoraline (**277**), a potent antitumor thiodepsipeptide produced by *Micromonospora* sp., had been determined by total synthesis (Boger and Ichikawa 2000; Perez Baz et al. 1997; Romero et al. 1997). As the lead compound, **277** showed nmol/L-level cytotoxicity against a series of cancer cells both in vitro and in vivo, such as lung, colon carcinoma and melanoma cells. Additionally, its antiviral and antibacterial activities against several strains of Gram-positive bacteria were demonstrated (Boger et al. 2001; Faircloth et al. 1997; Romero et al. 1997). *Verrucosispora* sp. also yielded five thiocoraline congeners **278**–**282**. 12′-Sulfoxythiocoraline (**279**) and thiochondrilline C (**282**) exhibited potent cytotoxicity against A549 cells with EC_50_ values of 1.26 and 2.86 μmol/L but not as good as **277** with an EC_50_ value of 0.0095 μmol/L. According to studies on structure–activity relationships, both 3-OH-quinoxaline and two phenol groups were identified as the key contributors to the bioactivity (Boger et al. 2001; Wyche et al. 2011). Research on the culture of *Streptomyces cyaneofuscatus* confirmed C_32_H_24_N_6_O_10_S_2_ (**283**)_._ The disulfide derivative was obtained by the spontaneous dimerization of compound C_16_H_13_N_3_O_5_S (**284**) in solution (Ortiz-López et al. 2018).

**Fig. 2:**
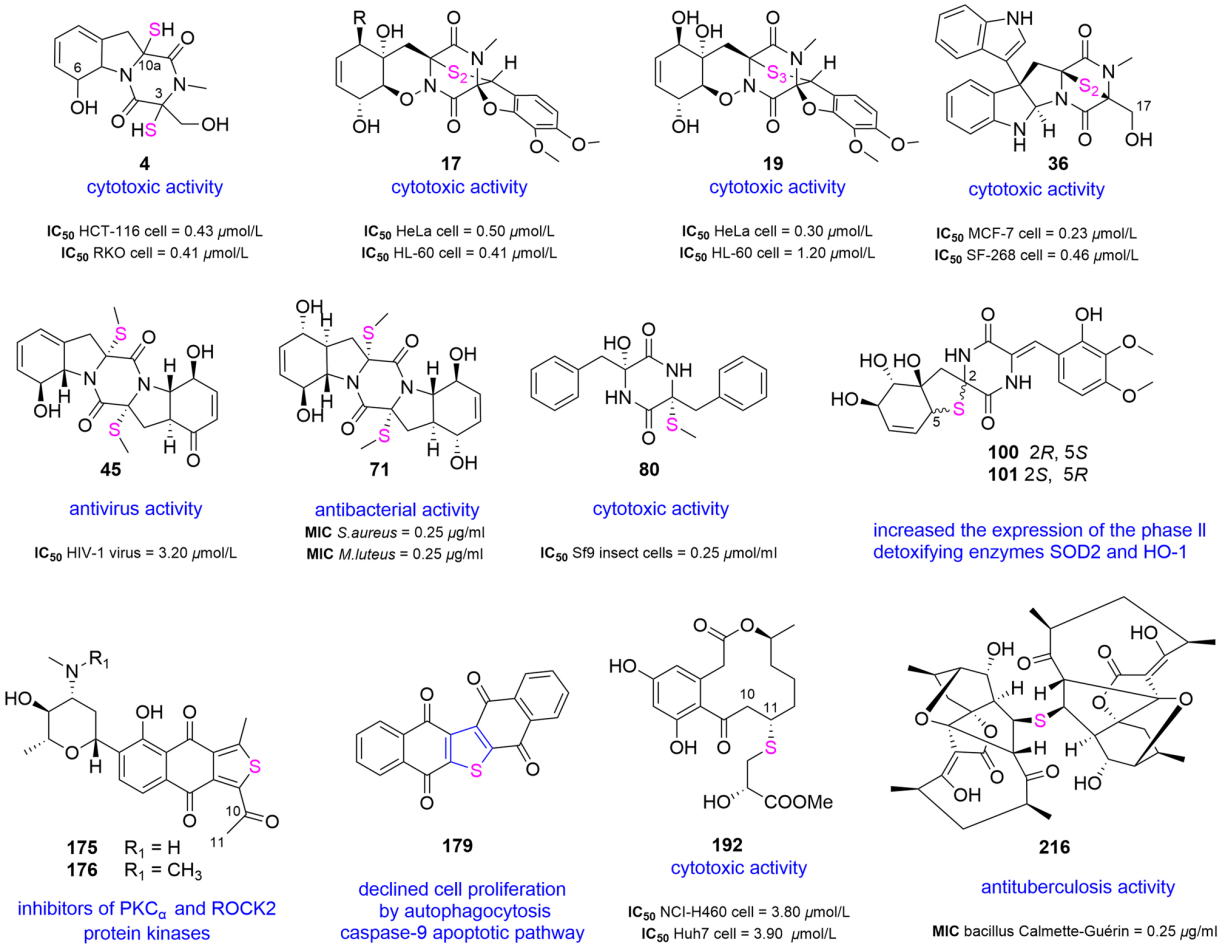
Representatives of thioethers compounds

A study of strain *Streptomyces* sp. successfully used a one strain–many compounds (OSMAC) strategy to assist the discovery of holomycin (**285**) and its two congeners (Ding et al. 2017). Compound **285** was a member of dithiolopyrrolone antibiotics and had a broad-spectrum antibacterial activity and strong cytotoxicity. It was found that it can interfere with the synthesis of bacterial RNA and exert its antibiotic activity by chelating intracellular metal ions, especially Zn^2+^ (Chan et al. 2017; Oliva et al. 2001). From the slight antibacterial activity of holomycin A (**286**) and (1Z)-*S,S'*-dimethyldihydroholomycin (**287**), we can know that the disulfide bond played a key role in the antibacterial ability (Ding et al. 2017). Different fermentation conditions yielded different metabolites. In a static fermentation condition, dithioaspergillazine A (**288**) obtained from *Trichoderma brevicompactum* had a strong cytotoxic effect, suggesting that the disulfide bond was necessary for the cytotoxic activity in such compounds (Yamazaki et al. 2016). A mixed assemblage of *L. majuscula*/*Schizothrix* sp. produced a NRPS/PKS hybrid, somocystinamide A (**289**). The cytotoxic disulfide dimer was sensitive to acid, and spontaneously transformed into its derivatives in acid (Nogle and Gerwick 2002). A *Blastobacter* sp. gave B-90063 (**290)**, an endothelin converting enzyme inhibitor (Takaishi et al. 1998) (Fig. 2, Supplementary Fig. S18).

### Thiazole/thiazoline-containing compounds

The thiazole ring is an important aromatic five-membered heterocyclic ring. The structure of this heterocyclic ring contains both nitrogen and sulfur atoms, which suggests that it is easy to form hydrogen bonds and can coordinate with non-metal ions and hydrophobic interactions. The diverse physical and chemical properties have determined that thiazole rings have broad application prospects in prosperous fields such as chemistry, pharmacy, biology and materials science.

### Peptides

The thiazole group-containing peptides also account for a large proportion of sulfur-containing compounds derived from marine microorganisms.

Apratoxins A–H (**291**–**298**) and apratoxin A sulfoxide (**299**) were a group of cytotoxic cyclic depsipeptides isolated from marine cyanobacteria, for instance, *Lyngbya majuscule*, *Lyngbya bouillonii*, *Lyngbya* sp*.* and *M. producens* (Gutierrez et al. 2008; Luesch et al. 2001b, 2002b; Matthew et al. 2008b; Thornburg et al. 2013; Tidgewell et al. 2010). It had been confirmed that apratoxin A (**291**) prevented co-translational translocation of proteins destined for the secretory pathway. The anticancer efficacy of **291** was achieved by down-regulating cancer related proteins simultaneously to reduce the intracellular content (Liu et al. 2009). Although **291** exhibited excellent cytotoxicity in vitro and antitumor effect in vivo, the poor selectivity to different cancer cells and a small therapeutic window limited its potential role as an antitumor drug (Tidgewell et al. 2010). In addition to natural products, the derivatives of apratoxins through total synthesis or semi-synthesis further clarified its structure–activity relationships and mechanism. **291** inhibited the secretory pathway at the level of co-translational translocation to cure cancer. Apratoxin S4 was the first viable candidate that showed the requisite tumor selectivity and increased antitumor activity and potency in the apratoxin family (Chen et al. 2011) (Table 3, Supplementary Fig. S19).

**Table 3:** IC_50_ of cytotoxic activity data for apratoxins (unit: nmol/L)

Cell line compound	LoVo	KB	NCI-H460	HT29	HeLa	U2OS	HCT-116
Apratoxin A (**291**)	0.38	0.52		1.40	10.00	10.00	5.97
Apratoxin B (**292**)	10.80	21.30					
Apratoxin C (**293**)	0.73	1.00					
Apratoxin D (**294**)			2.60				
Apratoxin E (**295**)				21.00	72.00	59.00	52.10
Apratoxin F (**296**)			2.00				36.00
Apratoxin G (**297**)			14.00				
Apratoxin H (**298**)			3.40				
Apratoxin A Sulfoxide (**299**)			89.90				

Fermentation of *Lyngbya confervoides* led to the production of obyanamide (**300**) (Williams et al. 2002), and its C-3 stereochemistry had been revised by total synthesis (Zhang et al. 2006). Usually, this type of cyclodepsipeptide contains two *N*-methyl amino acids, an Ala-thiazole unit, α-amino acid and *β*-amino acid. Ulongamides A–F (**301**–**306**), several structural analogs to **299**, had also been isolated from *Lyngbya* sp. All but ulongamide F (**306**) demonstrated moderate cytotoxic activity against KB and LoVo cells (Luesch et al. 2002a). Kakeromamide A (**307**) isolated from *Moorea bouillonii* was the first marine cyclic peptide inducing neural stem cell (NSC) differentiation. Compound **307** induced NSCs into astrocytes in a dose-dependent manner in vitro without cell death and exhibited moderate cytotoxicity against HeLa cells (Nakamura et al. 2018).

*Lyngbya confervoides*, *Leptolyngbya* sp. and *L. majuscula* were the sources of grassypeptolides A–G (**308**–**314**), which contained two thiazolines and several unnatural amino acids, including *D*-amino acid, *N*-methyl amino acid, 2-aminobutyric acid and *β*-amino acid. They demonstrated strong or potent cytotoxic activity against several HTCLs (Kwan et al. 2008, 2010; Thornburg et al. 2011). Structure–activity relationships showed **310** had 16–23-fold higher cytotoxic activity than **308** because the configuration of C-29 was converted from *S* to *R*. In addition, **308** and **310** caused G_1_ phase arrest at low concentrations, while at high concentrations they induced cell cycle arrest at the G_2_/M phase. Moreover, **313** and **314** were identified as the inhibitors of transcription factor AP-1 (Kwan et al. 2010). Attracted by these characteristics, the total synthesis of **308** had been achieved. The whole synthesis includes 17 steps, with a total yield of 11.3% and an average of 88% per step (Liu et al. 2010) (Supplementary Fig. S20).

Alotamide A (**315**), originally isolated from *L. bouillonii*, significantly promoted the calcium influx in mouse neurons. The absolute configuration of polyketide fragment (C_15_**–**C_32_) was not solved due to the limitation of quantity (Soria-Mercado et al. 2009). The complete stereochemistry of **315** was assigned by asymmetric synthesis in which four possible diastereomers of indefinite fragments were designed (Shi et al. 2018a). Investigation of active antimalarial components from the cyanobacterium *Oscillatoria* sp. resulted in the isolation of venturamides A (**316**) and B (**317**). Both of them existed selective antimalarial activity against *P. falciparum* but mild activity against other tropical parasites and mammalian cells (González and Gerwick 2007). Investigation of cyanobacterium *L. majuscule* resulted the isolation of dolastatin 3 (**318**), homodolastatin 3 (**319**) and kororamid (**320**), among which **318** inhibited HIV-1 integrase with an IC_50_ value of 5 mmol/L (Mitchell et al. 2000). Furthermore, **318** originally found in sea hare *Dolabella auricularia*, may be produced by the cyanobacteria diet of sea hare (Pettit et al. 1982, 1987). By comparison with the retention time of synthetic compounds in HPLC, the stereochemistry of *N,N-*dimethylisoleucine moiety was assigned (*S, S*) in symplostatin 1 (**321**) (Harrigan et al. 1998; Luesch et al. 2001a). Also, as a potent cytotoxic agent and potent microtubule inhibitor, it was similar in structure to dolastatin 10 except for the methyl group at C-28. Moreover, it has been confirmed that **321** shows cytotoxic activity in vitro against KB and LoVo cells and in vivo activity against some murine tumors. After a small dose of intravenous injection of **321**, its toxicity would cause lethality on the first day (Luesch et al. 2001a).

Hoiamides (**322**–**325**) were a class of lipopeptides bearing one thiazole and two consecutive α-methylated thiazolines fragments. Hoiamides A (**322**) and B (**323**) were obtained from the assemblages of *L. majuscula* and *Phormidium gracile*, *Symploca* sp. and *Oscillatoria* cf. sp., respectively (Choi et al. 2010; Pereira et al. 2009). As the site 2 partial agonists, **322** and **323** can effectively inhibit the activity of cortical neurons in mouse, whilst hoiamides C (**324**) and D (**325**) with linear structure had no activity. The comparison of major differences indicated that the macrocycle was the core structure. Toxicity tests on brine shrimp showed that **324** was toxic. Compound **325** displayed strong inhibitory activity against p53/MDM2 protein binding (Choi et al. 2010; Malloy et al. 2012; Pereira et al. 2009) (Supplementary Fig. S21).

Lyngbyabellins family was a class of cytotoxic peptides promoting of actin polymerization, and 18 members (**326**–**343**) had been identified over the past 21 years. *Lyngbya, Moorea* and *Okeania*, these three cyanobacterial genera were regarded as the main sources of lyngbyabellins (Choi et al. 2012; Han et al. 2005; Luesch et al. 2000b, c, 2002c; Matthew et al. 2010; Milligan et al. 2000; Petitbois et al. 2017; Williams et al. 2003). All compounds contained two thiazole functional groups except lyngbyabellin B (**327**), which contained one thiazole ring and one thiazoline ring. Biosynthetically, these lyngbyabellins with a mixed polyketide-peptide construction may derive from an assembly by NRPS and PKS (Gerwick et al. 2001). Interestingly, some complex metabolites had been discovered one after another, for example, lyngbyabellins D (**329**) and N (**339**). In addition to the skeleton of lyngbyabellins, this molecule also exhibited an *N,N*-dimethylvaline side chain similar to dolastatin 10 (Choi et al. 2012). Considering the close relationship with the animal toxin, dolabellin, the following hypotheses had been proposed. These secondary metabolites were produced by marine cyanobacterial organisms, and sea hares in the environment absorb these metabolites when they eat cyanobacteria, and biotransformation occurs in the process (Han et al. 2005). It can be inferred that the ester bond at C-24 or C-16 position was prone to methanolysis and a regioselective ester cleavage, yielding corresponding products. In the research of lyngbyabellin C (**328**), it was found that the ester bond at C-16 tended to be methanolized. Consequently, homohydroxydolabellin, the possible artifact, is formed by selective ester cleavage (Luesch et al. 2002c). Similarly, lyngbyabellins F (**331**) and I (**334**) may be converted from lyngbyabellins E (**330**) and H (**333**) due to the methanol used in the extraction. Another interesting example was lyngbyabellin O (**340**), which may be formed by lyngbyabellin G (**332**) or regioselective ester cleavage of **331** or lyngbyabellin P (**341**) at C-24 (Han et al. 2005). It is precisely because these metabolites can be transformed into each other through simple conditions that the members of the lyngbyabellins family are so abundant. However, the question of whether it is an artifact should be treated with caution (Supplementary Fig. S22).

Regardless of whether linear peptide or cyclic peptide, the structure–activity relationships of lyngbyabellins were predicted that the increasing number of chlorine atoms and the presence of side chains can improve cytotoxic activity. But the relationship is far from simple, and some lyngbyabellins without side chains also showed potential activity, for instance, **327**. Rich structures brought diverse biological activities. Not only cytotoxic activities but also other activities were observed in lyngbyabellins. Compounds **340** and **341** exhibited potent antifouling activity with EC_50_ values of 0.38 and 0.73 μmol/L. (Table 4) (Supplementary Fig. S23).

**Table 4:** The lyngbyabellins origin, cytoxicity and structure

Compound	Origin	Cytoxicity	Structure	Other activities
Lyngbyabellin A (**326**)	*Lyngbya majuscula*	++	Cyclopeptide	Disrupter of the cellular microfilament network
Lyngbyabellin B (**327**)	*Lyngbya majuscula*	++	Cyclopeptide	Toxicity toward brine shrimp; antifungal
Lyngbyabellin C (**328**)	*Lyngbya* sp.	+	Cyclopeptide	–
Lyngbyabellin D (**329**)	*Lyngbya* sp.	++	linear peptide	–
Lyngbyabellin E (**330**)	*Lyngbya majuscula*	++	Cyclopeptide	–
Lyngbyabellin F (**331**)	*Lyngbya majuscula*	++	linear peptide	–
Lyngbyabellin G (**332**)	*Lyngbya majuscula*	+	Cyclopeptide	–
lyngbyabellin H (**333**)	*Lyngbya majuscula*	++	Cyclopeptide	–
lyngbyabellin I (**334**)	*Lyngbya majuscula*	++	linear peptide	–
lyngbyabellin J (**335**)	*Lyngbya bouillonii*	++	Cyclopeptide	–
lyngbyabellin K (**336**)	*Moorea bouillonii*	−	Cyclopeptide	–
lyngbyabellin L (**337**)	*Moorea bouillonii*	−	Cyclopeptide	–
lyngbyabellin M (**338**)	*Moorea bouillonii*	−	linear peptide	–
lyngbyabellin N (**339**)	*Moorea bouillonii*	++	Cyclopeptide	–
lyngbyabellin O (**340**)	*Okeania* sp.	−	linear peptide	Antifouling activity
lyngbyabellin P (**341**)	*Okeania* sp.	+	linear peptide	
27-deoxylyngbyabellin A (**342**)	*Lyngbya bouillonii*	++	Cyclopeptide	
7-*epi*-lyngbyabellin L (**343**)	*Moorea bouillonii*	−	Cyclopeptide	

++ represent potent cytotoxicity (IC_50_ < 0.5 μmol/L); + represent strong or moderate cytotoxicity (IC_50_ = 0.5–5 μmol/L); − represent inactivity

Anti-proliferative hectochlorin (**344**) with no cytotoxicity was discovered from *L. majuscule,* which was described as a potent promoter of actin polymerization and an efficient fungicidal compound against *C. albicans* (Marquez et al. 2002). Additionally, molecular networking combined with genome sequencing analysis guided the isolation of hectochlorins B–D (**345**–**347**) (Boudreau et al. 2015). Lyngbyapeptins A–D (**348**–**351)** and 15-norlyngbyapeptin A **(352)** were a series of linear peptides derived from the genus *Lyngbya* (Klein et al. 1999; Luesch et al. 2000b, 2002c; Matthew et al. 2010; Williams et al. 2003). It was observed that the unstable **351** resulted in its decomposition (Matthew et al. 2010). Nevertheless, compared with co-isolated compounds in the lyngbyabellins family, lyngbyapeptins were not found to display any activity. Apramides A–G (**353**–**359**) were a group of linear depsipeptides possessing olefin or alkyne moieties from *L. majuscule* (Luesch et al. 2000a). The potent cytotoxic component of *Symploca* sp. was identified as micromide (**360**), which consisted of three *N*-methyl amino acids as well as two amino acids. Despite the similarity between the structures of **360** and apramides, the IC_50_ of the **360** against KB cells was an order of magnitude greater than the latter (Williams et al. 2004) (Supplementary Fig. S24).

Mechercharstatin A (former name: mechercharmycin A) (**361**) and urukthapelstatin A (**362**) containing oxazoles and thiazoles, were potent cytotoxic metabolites isolated from *Thermoactinomyces* sp. and *Mechercharimyces asporophorigenens*, respectively (Kanoh et al. 2005, 2007; Matsuo et al. 2007a, b). Compared with their analogs, the rigidity and sequential aromatic heterocyclic cyclic structure were necessary for their significant cytotoxic activity. Due to the remarkable bioactivities, biomimetic synthesis by aza-Wittig ring contraction and total synthesis of **362** had been achieved (Lin et al. 2013; Schwenk et al. 2016). Unexpectedly, **362** was not stable, because the Z/E configuration of the double bond at C-8/C-9 could be transformed in solution (Schwenk et al. 2016). A culture of *Marinactinospora thermotolerans* produced a consecutive *tris* thiazole-thiazoline-containing metabolite, marthiapeptide A (**363**), which exhibited not only potent cytotoxic properties but also antibacterial effect against Gram-positive bacteria (Zhou et al. 2012).

Nocathiacins I–III (**364**–**366**) were obtained from the actinomycete *Nocardia* sp. These antibiotics demonstrated potent in vitro activity against a wide spectrum of 17 strains of Gram-positive bacteria containing several multiple-drug-resistant bacteria (MDR), and showed excellent efficacy in the systemic *S. aureus* infection mice model in vivo (Leet et al. 2003). However, the poor water solubility of nocathiacins limited further research. To improve water solubility, a series of nocathiacin derivatives had been synthesized (Naidu et al. 2006). Further investigation of *Nocardiopsis* sp. led to the isolation of TP-1161 (**367**), featuring an uncommon aminoacetone moiety. Compound **367** displayed antibacterial activity against several Gram-positive strains (Engelhardt et al. 2010a). Furthermore, it was discovered that the biosynthetic gene cluster of **367** comprised 13 open reading frames (Engelhardt et al. 2010b). As a new approach to the discovery of natural products, genome mining led to the isolation of trichamide (**368**) from cyanobacterium *Trichodesmium erythraeum.* The planer structure of **368** was determined by MS/MS fragmentation pattern. However, the stereochemistry was just inferred and not supported by experiment data (Sudek et al. 2006). Kocurin (**369**) was an antibacterial metabolite of the bacterium *Kocuria palustris.* It displayed strong antibacterial activity against Gram-positive bacteria including MRSA, whilst no obvious effect on Gram-negative bacteria and *Canidia albicans* (Martin et al. 2013).

YM-266183 (**370**) and YM-266184 (**371**) exhibited potent antibacterial activities against several types of pathogenic bacteria involving MDRs. However, they were not sensitive to Gram-negative bacteria (Suzumura et al. 2003a, b). Litoralimycins A (**372**) and B (**373**) were isolated from *Streptomonospora* sp. while they had no antibacterial activity and **372** displayed moderate cytotoxicity against several cell lines (Khodamoradi et al. 2020). A bioassay-guided approach applied to search for novel cytotoxic compounds from cyanobacteria *Lyngbya* sp. This led to the isolation of bisebromoamide (**374**) and norbisebromoamide (**375**), which possessed a group of *D*-amino acids, bromine atom and a rare 2-(1-oxopropyl) pyrrolidine moiety unprecedented in natural products (Sasaki et al. 2011; Teruya et al. 2009). The stereochemisty of **374** was revised later by total synthesis (Gao et al. 2010). These peptides showed potent anti-proliferative activity against a class of HTCLs. Moreover, **374** played the role of protein kinase inhibitor. Extracellular Signal Regulated Protein Kinase (ERK) pathway was inferred as the target of compound **374**, which acted against anomalous activated cells of the ERK-MAP pathway without side-effects (Sasaki et al. 2011). The structure–activity relationships were analyzed using synthetic derivatives. When the methyl group at C-17 changed, the cytotoxicity disappeared, whereas the bromine atom, methyl group at C-4, phenolic hydroxyl group, and the stereochemistry of methylthiazoline only produced slight effects (Li et al. 2011). Unreported strong sterol *O*-acyltransferase (SOAT) inhibitors, the linear lipopeptides were identified as biseokeaniamides A–C (**376**–**378**), which were obtained from *Okeania* sp. Their inhibitory effects were not merely at an enzyme level but also a cellular level. Additionally, the relatively low activity of **378** meant that the existence of the *N*-methyl moiety in *N*-Me-Val enhanced the SOAT-inhibitory activity. Furthermore, **377** displayed significant cytotoxicity and induced apoptosis in HeLa cells and activated caspase 3 dose-dependently (Iwasaki et al. 2017). Compound **376** had anti-inflammatory effects through selective inhibition of LPS-induced signal transduction (Ohno et al. 2020).

Kalkitoxin (**379**), a hybrid NRPS/PKS lipopeptide from *L. majuscula*, proved to be a potent anti-inflammation metabolite. Compound **379** not only inhibited cell division but was also a highly effective blocker of voltage sensitive-sodium channels in mouse neuro-2a cells (Wu et al. 2000). Furthermore, as the inhibitor of *N*-methyl-*D*-aspartate receptor antagonists, it displayed exposure time-dependent neurotoxic activity in cerebellar granule neurons (Berman et al. 1999). The metabolite also demonstrated potent cytotoxicity, and structure–activity relationships clarified the considerable role of thiazoline (White et al. 2004). In addition, the mechanism of cytotoxicity was discussed. As an HIF-1 inhibitor, it disrupted mitochondria-mediated oxygen consumption by suppressing the multi-enzyme mitochondrial NADH-ubiquinone oxidoreductase system (Morgan et al. 2015). *Caldora penicillata* also yielded three mixed PKS/NRPS metabolites, laucysteinamide A (**380**) along with two known compounds (Zhang et al. 2017a) (Supplementary Fig. S25).

### Benzothiazoles

Para-terphenyl with a tricyclic or polycyclic C-18 aromatic skeleton presented mainly in fungi showed diverse biological activities (Li et al. 2018b). Interestingly, the following four compounds **381**–**384** were all derived from actinomycete (Deng et al. 2014). Nocarterphenyl A (**383**) with potent cytotoxicity along with nocarterphenyl B (**384**) were obtained from *Nocardiopsis* sp. (Wang et al. 2019). *Alternaria* sp. was observed to produce three new compounds. Among them, altenusinoides A (**385**) and B (**386**) contained a rare altenusin/thiazole hybrids 6/6/5 framework. Intriguingly, methyl 2-(6-hydroxybenzothiazol-4-yl) acetate (**387**) was the first benzothiazole derivative obtained from fungi (Chen et al. 2018). In the application of high-throughput screening technology, the investigation of bacterium *Erythrobacter* sp. revealed two benzothiazole diterpenes, erythrazoles A (**388**) and B (**389**). Compound **388** lacked only an olefin moiety, and it did not show the same level of cytotoxic activity as that of **389** (Hu and MacMillan 2011). Four benzothiazoles **390**–**393** were obtained from *Micrococcus* sp. (Stierle et al. 1991).

*Bacillus* sp., *Bacillus endophyticus* and *Bacillus vallismortis* produced four tryptamide thiazole metabolites, bacillamides A**–**C (**394**–**396**) and neobacillamide A (**397**) (Jeong et al. 2003; Socha et al. 2007; Yu et al. 2009). Previously, comparing compound CD spectra with their known analogs were used to determine the absolute configuration. However, the organic syntheses of **395**–**397** revised the C-13 stereochemistry of these compounds (Bray and Olasoji 2009; Martínez and Davyt 2013). To some extent, comparing CD spectra is not completely trustworthy, especially when there is only marginal difference among analogs of various substituents. In particular, **394** showed strong algicidal activity against *Cochlodinium polykrikoides* with LC_50_ of 3.2 μg/ml. As the potent algaecides, this type of compound targets red tide algae. Meanwhile, synthetic aniline-derived analogs **394a**–**394f** exhibited higher algicidal activity (EC_50_ = 4.0–33.9 mg/L) against three freshwater harmful algae (*Mycrocyctis aeruginosa*, *Scenedesmus obliquus*, *Chlorella pyrenoidosa*) than **394** (EC_50_ = 19.33–250.1 mg/L**)** (Wang et al. 2017) (Supplementary Fig. S26).

### Others

A biosynthetic gene cluster with a great quantity of Fe(II)/α-ketoglutarate-dependent halogenases was identified in the genome of *Fischerella* sp. Above-mentioned genome analysis combined with mass spectrometry led to the isolation of aranazoles A–D (**398**–**401**), which were a panel of unusual hybrid highly chlorinated nonribosomal peptide–polyketide metabolites. However, **398** had no significant activity including antiviral, cytotoxic and antimicrobial activities (Moosmann et al. 2018). Chlorosulfolipids were a type of halogenated compounds isolated from algae and mussels (Ciminiello et al. 2002). Considering the similarity between aranazoles and chlorosulpholipids, it was supposed that they had similar biosynthetic enzymes and chlorosulpholipids were produced by related cyanobacteria (Moosmann et al. 2018).

Barbamide (**402**) was a mixed polypeptide–polyketide molluscicidal agent isolated from *L. majuscula* in 1996. It is worth mentioning that **402** contained a unique trichloromethyl group, *β*-methoxy amide and a thiazole unit (Orjala and Gerwick 1996). Owing to the uncommon structural features, a range of feeding experiments with stable isotopes-labeled substrates confirmed that the chlorination was accomplished by the tandem action of two halogenases using leucine, with the first involving the participation of at least two halogen atoms and the second achieving the conversion to a trichloromethyl moiety (Flatt et al. 2006; Galonić et al. 2006). Additionally, the nitrogen atom in the thiazole ring stemmed from glycine (Sitachitta et al. 2000). In the process, some new natural products **403**–**408** were also excavated (Balunas et al. 2010; Flatt et al. 2006; Jiménez and Scheuer 2001; Kim et al. 2012; Sitachitta et al. 2000). Given inactive dechlorobarbamide (**407**), and 4-*O*-demethylbarbamide (**408**) with much stronger activity than **402**, structure–activity relationships revealed that the carbonyl at C-4 and the trichloromethyl at C-2 improved molluscicidal activity. Mass spectrometry analysis of *Trichodesmium* sp. impelled the discovery of trichothiazole A (**409**), which possessed a terminal alkyne, two vinyl chlorides and displayed moderate cytotoxic activities against Neuro-2A cells (Belisle et al. 2017). Curacins A–D (**410**–**413**) were mixed PK/NRP compounds from *L. majuscula* (Gerwick et al. 1994; Márquez et al. 1998; Yoo and Gerwick 1995). Compound **410** displayed potent anti-proliferative and antimitotic activity against a panel of HTCLs with IC_50_ values ranging from 7 to 200 nmol/L. In addition, as a microtubule inhibitor, **410** interacts with the colchicine binding site on tubulin to block cell cycle progression (Blokhin et al. 1995).

Phenyl thiazole or thiazoline compounds occupied a certain proportion of MNPs. A screening strategy of broadly targeted biological evaluation was applied in the isolation of neuroactive pulicatins A–E (**414**–**418**) from *Streptomyces* sp. In the assay of dorsal root ganglion neurons in mice, **414** decreased Ca^2+^ influx while aerugine, with a difference in the methyl group at C-6′, showed the opposite phenomenon. Furthermore, **414** and **417** were tested and displayed highly selective binding activity to the 5-HT_2B_ receptor. More thiazolines in the molecule may reduce the activity. Compared to the strongest activity of **414** containing only one thiazoline ring, the co-isolated watasemycins A and B with two thiazolines were weaker (Lin et al. 2010). In subsequent research, 30 derivatives were designed to analyze the structure–activity relationships of pulicatins, which indicated that the 2-arylthiazoline scaffold was the adjustable serotonin receptor-targeting pharmacophore. Additionally, **415** revealed the remarkable antiseizure and antinociceptive effects in vivo (Lin et al. 2017). Anithiactins A–C (**419**–**421**) were obtained from *Streptomyces* sp. and soon afterwards *Actionomycetospora* sp. was found to yield thiasporine A (**422**), **419** and **421**, which possessed a 2-phenylthiazole moiety (Fu and MacMillan 2015b; Kim et al. 2014). Structure revision of **422** was completed by the synthesis of 2-aminophenylthiazinone derivatives. By comparing the NMR spectrum data of previously separated **422** and synthetic products, the possibility that natural product existed in the form of a carboxylate was raised (Seitz et al. 2016). In the acetylcholinesterase inhibitory test, **419**–**421** exhibited weak inhibitory effects. In addition, only **419** showed moderate cytotoxicity against H2122 cells. It seemed that the methyl group linked to an amino group decreased the activity. Afterward, Suzuki–Miyaura cross-coupling was applied in the total synthesis of these four metabolites (Vaaland et al. 2019).

**Fig. 3:**
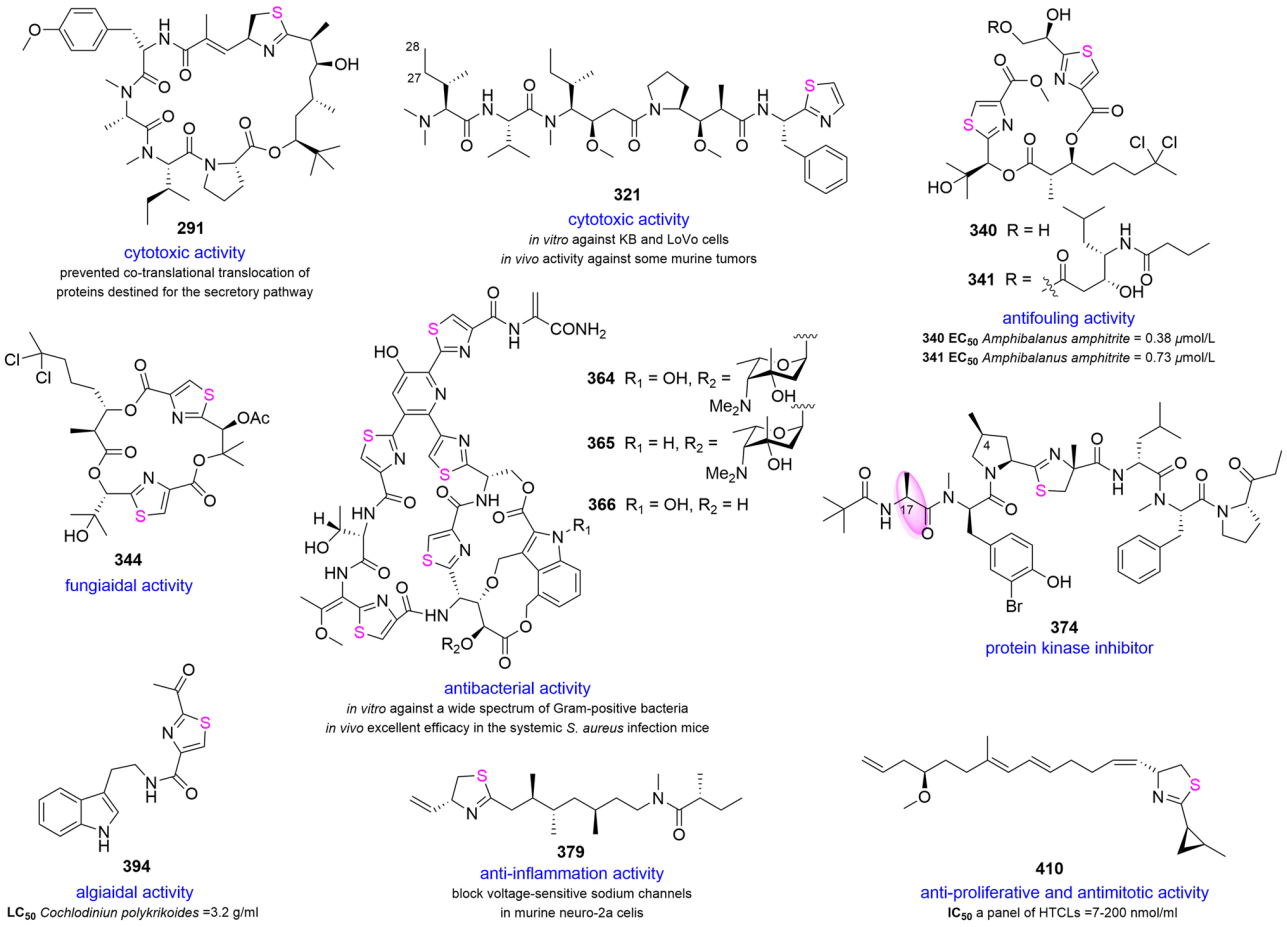
Representatives of thiazole/thiazoline-containing compounds

Cultures of a bacterium *Agrobacterium* sp. were reported to yielded agrochelin (**423**), which demonstrated chelating properties to the Zn^2+^ ion and strong cytotoxicity in vitro but its acetylated derivative was much weaker (Cañedo et al. 1999). Subsequently, its diastereomer, massiliachelin (**424**), was detected in a genome sequence analysis of *Massilia* sp. The result disclosed that the alkaloid was predominantly produced under an iron-deficient environment by comparing fingerprints in different conditions (Diettrich et al. 2019). Other new analogs containing an uncommon heterocyclic structure, ulbactins F (**425**) and G (**426**), were obtained from *Brevibacillus* sp. Their inhibitory activities on tumor cell migration had been proven. The investigation of *Saccharomonospora* sp. described the discovery of lodopyridones A–C (**427**–**429**) which exhibited weak inhibitory activities on the *β*-site amyloid precursor protein cleaving enzyme 1. Additionally, **427** exhibited modest cytotoxicity in HCT-116 cells (Le et al. 2017; Maloney et al. 2009). Due to the challenge in structure, **427** was subsequently synthesized in nine steps with an overall yield of 23% (Burckhardt et al. 2012). Acaromyester A (**430**) was characterized from the fungus *Acaromyces ingoldii* with no activity (Gao et al. 2016). Culture of *Streptomyces fradiae* produced two indolocarbazoles, fradcarbazoles A (**431**) and B (**432**). They exhibited significant cytotoxicity against a panel of HTCLs and inhibited the kinase PKC-α with IC_50_ values of 0.001–4.58 μmol/L (Fu et al. 2012). Anguibactin (**433**) was a siderophore from *V. anguillarum*. Its structure determination benefited from anhydroanguibactin (**434**) (Jalal et al. 1989; Lee et al. 2018) (Fig. 3, Supplementary Figs. S27, S28).

## Sulfoxides and sulfones

The sulfoxide exists in two configurations, *R* and *S*, resulting in the doubling of several signals in the 1D NMR spectra. Techniques such as 2D NMR including COSY, HMBC and TOCSY were extensively employed. Sulfoxide and sulfone are probably artificial products formed by oxidation of natural products containing methionine in ambient atmosphere during the separation process (Ogino et al. 1996; Yokokawa and Shioiri 2002).

### Sulfoxides

Anabaenopeptins NP 867 (**435**), 869 (**436**) and 883 (**437**) were identified in extracts of cyanobacterial bloom material composed of *Nodularia spumigena*, *Aphanizomenon flos-aquae* and *Dolichospermum* spp. using LC–MS/MS techniques. They were found to be a moderate inhibitor of carboxypeptidase A and protein phosphatase (Spoof et al. 2015). Pompanopeptin A (**438**) was a selective inhibitor of trypsin and chymotrypsin in vitro with an IC_50_ value of 2.4 ± 0.4 μmol/L, and its selectivity was conferred by arginine residues (Matthew et al. 2008a). Symplostatin 2 (**439**) and somamide A (**440**) were dolastatin 13 analogs, and isolated from *Symploca hydnoides* and assemblages of the cyanobacteria *L. majuscula* and *Schizothrix* sp., respectively (Harrigan et al. 1999; Nogle et al. 2001). The investigation of cyanobacterium *L. confervoides* led to the characterization of three new cyclodepsipeptides including tiglicamide C (**441**), a moderate inhibitor of porcine pancreatic elastase in vitro with an IC_50_ value of 7.28 µmol/L. Combining the co-isolated compounds, the structure–activity relationships revealed that carboxylic acid residue was not the necessary moiety to inhibit elastase activity (Matthew et al. 2009). Penilumamides family was the first reported natural lumazine peptides from fungi *Penicillium* sp. and *Aspergillus* sp. (Chen et al. 2014; Meyer et al. 2010). Biosynthetic feeding experiments on *Aspergillus* sp. using *L*-methionine suggested that *L*-methionine was a precursor of these lumazine peptides. The yield of penilumamide (**442**) and penilumamide B (**443**) was found to increase extremely in response to the concentration of the *L*-methionine in the medium. When **443** was exposed to air, the production of **442** and penilumamide C (**444**) were observed a few days later, which also once again verified the speculation of artificial products generated by oxidation (Chen et al. 2014). They were synthesized in eight steps from 1,3-dimethyllumazine-6-carboxylic acid using sequential saponification and amide coupling as the preparation methods (Reddy Penjarla et al. 2017) (Supplementary Fig. S29).

Two peptidic proteasome inhibitors, carmaphycins A (**445**) and B (**446**), containing a leucine-derived α, *β*-epoxyketone moiety, were obtained from *Symploca* sp. The absolute configurations of carmaphycins had been determined by total synthesis. Their strong capacity to inhibit the *β*5 subunit (chymotrypsin-like) of the *Saccharomyces cerevisiae* 20S proteasome was determined. Additionally, they displayed potent cytotoxicity to lung and colon cancer cells with IC_50_ values ranging from 9 to 19 nmol/L, as well as potent anti-proliferative effects to HTCLs (Pereira et al. 2012). The use of LC/MS-based metabolomics identified two antifungal polyketides, forazolines A (**447**) and B (**448**). Intriguingly, **447** exhibited antifungal activity against *C. albicans *in vivo without toxicity. Further research on the yeast chemical genomics revealed that **447** destroyed membrane integrity of fungi in a dose-dependent manner. Compound **448** was produced by increasing the concentration of KBr in the medium to help determine the position of the chlorine atom in **447** (Wyche et al. 2014). The first novel sulfur-containing angucyclinone with a unique ether-bridged system, grisemycin (**449**), was isolated from *S. griseus.* Additionally, it exhibited weak cytotoxic activity against HL-60 cells (Xie et al. 2016). *Salinispora pacifica* produced a class of nitrogen-containing volatiles which originated from biogenic amines derived from the amino acids valine, isoleucine and leucine. The structures of *N*-isobutylmethanesulfinamide (**450**) and *N*-isopentylmethanesulfinamide (**451**) were determined by total synthesis (Harig et al. 2017). Sydoxanthone C (**452**) was a kind of xanthone from *Aspergillus* sp. and communol D (**453**) obtained from *Penicillium commune* was the first known molecule of a naturally occurring aromatic polyketide with a sulfoxide functional group from marine fungi (Tian et al. 2015; Wang et al. 2012b). Quadricinctone B (**454**) was isolated from *Neosartorya quadricincta* and the single-crystal X-ray analysis established the absolute configuration (Prompanya et al. 2016) (Supplementary Fig. S30).

### Sulfones

Sulfonamides, such as sulfadiazine and sulfamethoxazole, are antibiotics routinely used worldwide. So far, few natural products containing aromatic sulfonamide or diarylsulfone have been discovered. Three unexpected sulfonyl-bridge alkaloid dimers, sulfadixiamycins A–C (**455**–**457**), were found in the recombinant *Streptomyces* sp. containing the biosynthetic gene cluster of xiamycin. The key role of flavoenzyme in the formation of these metabolites was confirmed. Sulfadixiamycins had moderate anti-mycobacterial activity without cytotoxicity or anti-proliferative effects. Additionally, **455** exhibited strong antibacterial activity (Baunach et al. 2015). From a biosynthetic perspective, alkaloid skeletons originate from amino acids. An amino acid directed strategy was applied to discover a series of metabolites in *Scedosporium apiospermum*. Scedapin C (**458**), the first example of fumiquinazoline bearing an aminosulfonyl group, showed high antiviral activity against the hepatitis C virus (Huang et al. 2017). Investigation of *Aspergillus* sp. also revealed a series of quinazoline-containing indole alkaloids, one of which was aspertoryadin A (**459**) bearing a similar structure to **458** (Kong et al. 2019). Scetryptoquivaline A (**460**) was a fumiquinazoline alkaloid isolated from *S. apiospermum* (Li et al. 2020) (Supplementary Fig. S31).

## Thioesters

As far as we know, naturally occurring secondary metabolites containing thioester groups are rare, and the thioester-type metabolites are mostly produced by sponges or bacteria. Chemical investigations of the marine microorganisms led to the isolation of compounds **461**–**472** (Boger and Ichikawa 2000; Han et al. 2019; Horton et al. 1990; Mahyudin et al. 2012; Perez Baz et al. 1997; Romero et al. 1997; Sata et al. 2005).

**Fig. 4:**
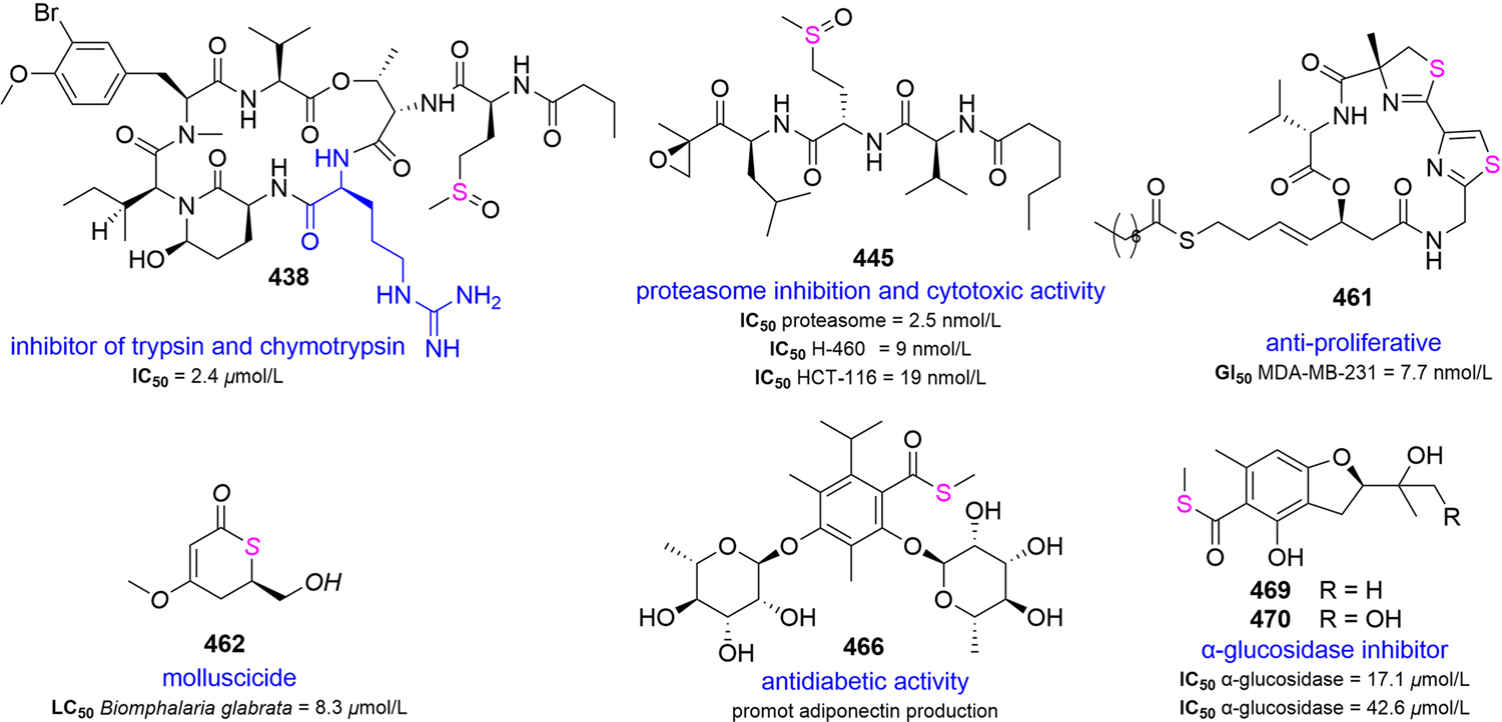
Representatives of other compounds

The potent anti-proliferative depsipeptide derived from *Symploca* sp., largazole (**461**) represented the first thioester reported from a marine cyanobacterium. Compound **461** displayed highly selective cytotoxicity towards transformed cancer cells in a dose-dependent manner. The absolute configuration was determined by ozone decomposition, followed by oxidation post-treatment and acid hydrolysis to produce optically active fragments. This natural product has attracted widespread attention (Taori et al. 2008a, b). Luesch and co-workers accomplished the first total synthesis of **461** in eight steps and identified histone deacetylases as the molecular targets (Ying et al. 2008). An assemblage of the cyanobacteria, cf. *Oscillatoria* and *Hormoscilla* spp. induced the second thioester, thiopalmyrone (**462**). The biodata for **462** highlighted its potential as a new molluscicide against the snail *Biomphalaria glabrata* with a LC_50_ value of 8.3 μmol/L (Pereira et al. 2011). Suncheonosides A (**463**), B (**464**) and D (**466**) from *Streptomyces* sp. have potential as an antidiabetic agents by promoting the production of adiponectin during adipogenesis in human mesenchymal stem cells in a concentration-dependent manner (Shin et al. 2015). Nitrosporeusines A (**467**) and B (**468**) possessing the novel skeleton, benzenecarbothioc cyclopenta[c]pyrrole-1,3-dione from *Streptomyces nitrosporeus* inhibited the H_1_N_1_ virus strongly in infected Madin-Darby canine kidney cells. They were first synthesized through allylic oxidation, enzymatic resolution and Michael addition in a scalable and green approach (Yang et al. 2013). A further study had confirmed that **467** was able to reduce the levels of nitric oxide, reactive oxygen species and pro-inflammatory cytokines (Philkhana et al. 2017). Eurothiocins A (**469**) and B (**470**), the potent competitive inhibitors of α-glucosidase, were isolated from fungus *Eurotium rubrum*. Even when compared with the clinically useful α-glucosidase inhibitor acarbose, their inhibition effects should be greater in vitro (Liu et al. 2014). By the development of an HPLC bioactivity profiling/microtiter technique in conjunction with microprobe NMR spectroscopy and access to the AntiMarin database, the efficiency of isolation and dereplication can be greatly improved (Mitova et al. 2008) (Fig. 4, Supplementary Fig. S32).

## Others

Chemical investigations on some bacteria strains led to the isolation of seven macrocyclic polydisulfides (**473**–**481**), which were a class of monocyclic or dimeric cyclic products of co-isolated metabolites. Meanwhile, their preferred conformations were predicted by DFT calculations and NMR spectroscopy. In the antimicrobial tests, **475** and **476** displayed antibiotic activities, while the remaining compounds were inactive (Ritzau et al. 1993; Sobik et al. 2007) (Supplementary Fig. S33).

Ammosamide A (**482**) was the first obtained natural product containing a thio-γ-lactam ring from *Streptomyces* sp. Another interesting feature of this metabolite was its specific nanomolar cytotoxicity against selected cancer cell lines including HCT-116 cells (Hughes et al. 2009). Artificial products had been summarized through the following two reasons by Chambers C. Hughes’ group. First, an artifact originated from reaction between an electrophilic site on a natural product and a nucleophilic metabolite or solvent. Second, it is relatively rare for natural products containing nucleophilic sites to react with electrophiles to form artificial products. Compound **482** was an interesting example of the latter. They confirmed ammosamide C can be spontaneously converted to **482** under weak base conditions, which was caused by the electrophilicity of the active imine functional group (Reimer and Hughes 2017).

Isothiocyanates are a class of compounds with the general formula R–N=C=S and have so far appeared only a few times in MNPs. Paulomycin G (**483**) was isolated from *Micromonospora matsumotoense* with moderate cytotoxicity against serval HTCLs (Sarmiento-Vizcaino et al. 2017). Hapalindole M (**484**) was an antibacterial and antifungal agent isolated from the cyanobacterium *Hupulosiphon fontinalis* (Moore et al. 1987) (Supplementary Fig. S34).

## Introduction of sulfur atoms

How is the sulfur atom introduced into molecules? The earliest feeding experiments dating back to gliotoxin and sirodesmin PL confirmed that a cyclo-dipeptide or an amino acid is the intermediate or it is interconverted with the intermediate (Bose et al. 1968; Ferezou et al. 1980; Kirby et al. 1978; Suhadolnik and Chenoweth 1958; Winstead and Suhadolnik 1960). Meanwhile, it was inferred that the introduction of sulfur occurs immediately following the cyclo-dipeptide formation (Pedras et al. 1990) and the sulfur in thiodiketopiperazine is derived from methionine, cysteine, and sodium sulfate (Gardiner et al. 2005). However, the mechanism of how the sulfur is introduced to a molecule is unknown. In 2011, Guo and co-workers comprehensively summarized the proposed biosynthesis hypothesis of thiodiketopiperazines (Jiang and Guo 2011) (Supplementary Fig. S35).

The assumption that glutathione is a direct donor of sulfur atoms is confirmed because the combination of a cyclic dipeptide intermediate and glutathione was found in the fermentation broth. Using gene knockouts and other molecular biological methods proved that *gliG* is responsible for encoding a glutathione sulfur transferase, GliG, which plays an important catalytic role in the sulfur transfer of glutathione into the diketopiperazine framework. In addition, it has been proved that *gliC* is the gene responsible for encoding a P450 monooxygenase, and the amino acid of the cyclo-dipeptide intermediate in α-position is oxidized to generate a hydroxyl group. It is a key step for introducing a sulfur atom to form a sulfur bridge (Cramer et al. 2006; Jiang and Guo 2011; Scharf et al. 2011).

It is the fusion of natural product chemistry and organic chemistry that enables MNPs to have adequate quality and wider application. Most molecules are also considered as synthetic targets, which further enhances their value as drug candidates.

Jiang and co-workers focused on the construction of sulfur-containing moieties in the total synthesis of natural products. Their reviews have been published on the total synthesis of sulfur-containing natural products via introducing sulfur atoms with different sulfurization agents and constructing related sulfur-containing moieties (Wang et al. 2020a, b, c). They summarized comprehensively the introduction of sulfur atoms into natural products and methods to construct sulfur-containing moieties in synthesis. For instance, H_2_S, S_8_, TrSCl, SO_2_, Na_2_S, Na_2_Me, NaSSO_2_Ph, P_2_S_5_, ClSO_3_H, RSH, AcSH, TMSSMe, cysteine and thiazole are common sulfurization reagents.

Overman and co-workers accomplished the total synthesis of (+)-leptosin D (**150**) and (+)-gliocladin A (**34**), installing the crucial *S*-methyl moiety by the participation of H_2_S (DeLorbe et al. 2013). Based on this route, the construction of disulfide bonds in molecule can be easily achieved (Supplementary Fig. S36).

In 2009, Movassaghi and co-workers completed the first asymmetric total synthesis of (+)-11,11-dideoxyverticillin A (**172**). First, the indole compound **a** was used as the starting material to obtain the tetracyclic skeleton compound **b**. CoCl(PPh_3_)_3_ was used as a catalyst to obtain the dimer compound **c** in the form of free radicals. Subsequently, K_2_CS_3_ was used as the sulfur source to introduce sulfur into the skeleton under oxidation conditions, and then the persulfide bridge was constructed (Kim et al. 2009) (Supplementary Fig. S37).

Aiming at the synthesis of luteoalbusins (**36** and **37**), a regioselective sulfuration method using H_2_S (g) and TFA was developed by Movassaghi and co-workers. The key steps are shown below (Adams et al. 2015) (Supplementary Fig. S38).

## Druggability

Natural products are considered to be an important source of innovative drug and lead compounds, such as artemisinin for malaria, paclitaxel for cancer and morphine for pain relief. The proportion of natural products and natural product derivatives in all new approved drugs is 22.7% (Newman and Cragg 2020).

Sulfur-containing drugs are widely used in the treatment of antibacterial, anti-inflammatory, skin diseases and cancer. The most important members are sulfa drugs with broad-spectrum antibacterial activity, and now they are not only used for antibacterial, but also expanded to the fields of hypoglycemia, anti-inflammatory, diuretic, anti-thyroid, anti-hypertensive, etc (Ilardi et al. 2014).

Much of the research in drugs is spurred by the rise of natural products. A number of potent sulfur-containing MNPs have been identified as potential lead compounds for further drug development, especially in the area of anticancer agents.

Staurosporine with a [2,3-α]pyrrolo[3,0.4-*c*]carbazole skeleton was isolated from a terrestrial actinomycete *Streptomyces staurosporeus* in 1977 (Bohonos et al. 1977). Biological activity studies revealed that the molecule exhibited significant cell proliferation inhibitory effects (Tamaoki et al. 1986). Due to the excellent biological activities, the focus of scholars on staurosporine has never been diminished. At present, the main representatives entering clinical research are UCN-01 (Bastians 2011), midostaurin (Kim 2017), edotecarin (Ciomei et al. 2006), lestaurtinib and becatecarin (Wishart et al. 2018). In 2006, The United States Food and Drug administration (FDA) granted orphan drug status to lestaurtinib for the treatment of acute myeloid leukemia (AML) (Bharate et al. 2013). Except for UCN-01, other staurosporine members that have entered clinical research are all organic-synthesized. They are used mainly for clinical research such as AML, breast cancer, prostate cancer, and hepatobiliary cancer. In 2017, midostaurin had been approved by the FDA for the treatment of adult patients with newly diagnosed Feline McDonough Sarcoma-like tyrosine kinase 3 mutation-positive AML (Kim 2017).

**Fig. 5:**
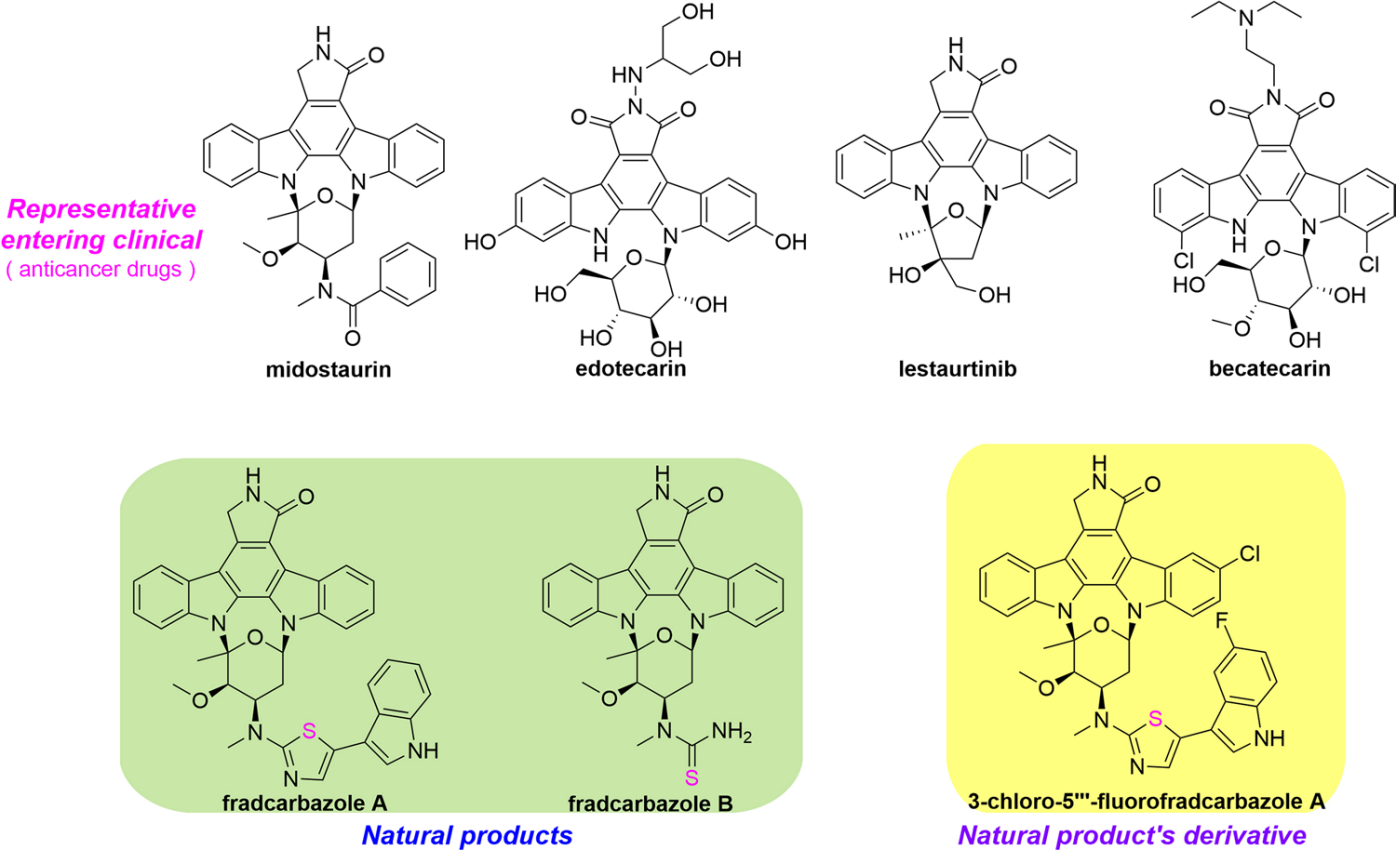
The chemical structures of representative drugs entering clinical studies, natural product and natural product derivatives

Marine actinomycetes also provide staurosporine-type compounds, for instance fradcarbazoles A (**431**), B (**432**) and C from *S. fradiae* (Fu et al. 2012). Based on **431**, the Zhu’s group designed a series of derivatives, where 3-chloro-5‴-fluorofradcarbazole A was considered to be a potential anti-AML agent. It induced apoptosis of the MV4-11 cells and arrested the cell cycle at the G_0_/G_1_ phase. Furthermore, it can downregulate p-FLT3, FLT3 and c-kit in a dose-dependent manner (Li et al. 2019; Wang et al. 2015) (Fig. 5).

**Fig. 6:**
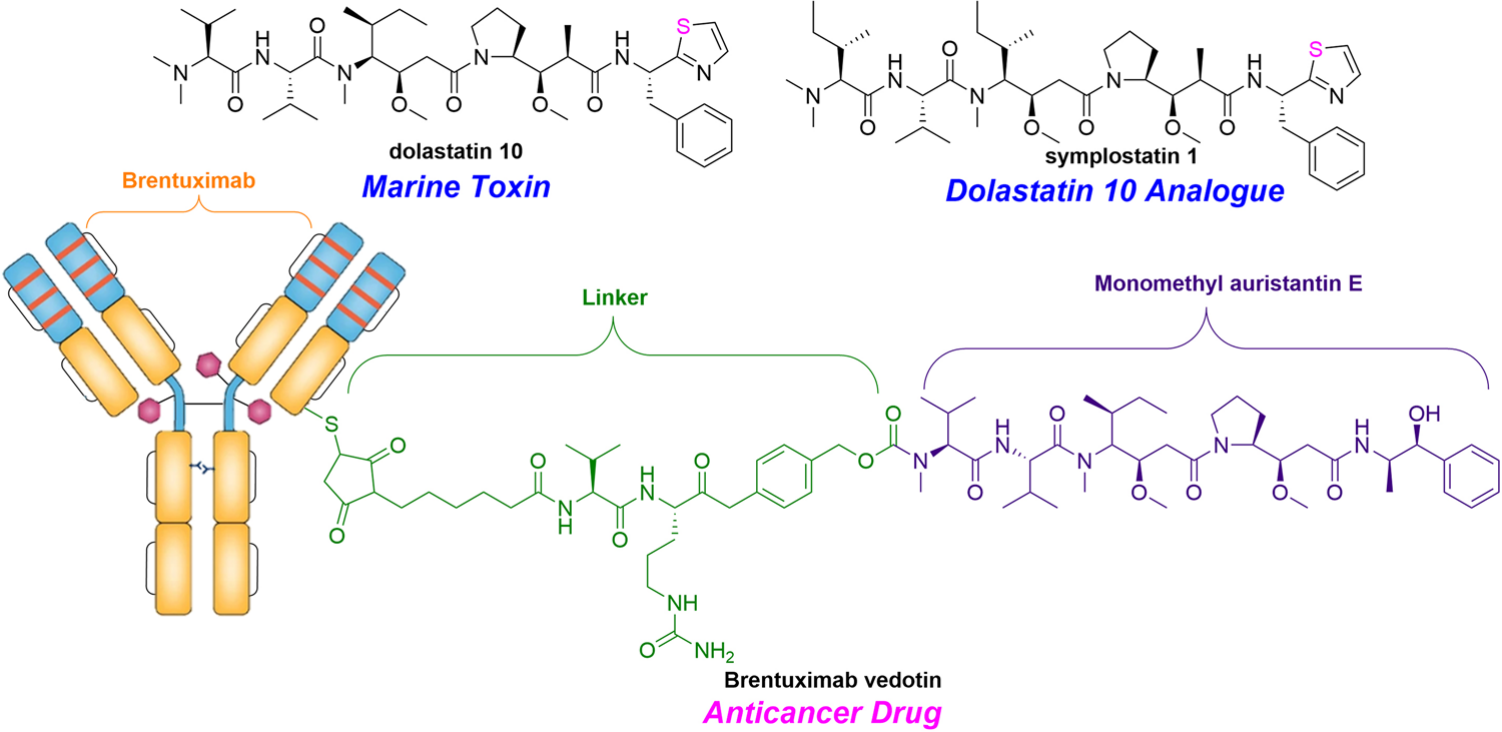
The chemical structures of dolastatin 10, symplostatin 1 and brentuximab vedotin

Dolastatins, a large family of peptides, were gradually isolated from the marine mollusk *D. auricular*ia. Later studies found that the metabolites were actually produced by cyanobacteria *S. hydnoides* and *L. majuscula *(Han et al. 2005). It is worth mentioning that dolastatin 10 has an IC_50_ of 0.046 ng/ml on P388 cells, which is one of the most active natural products found to date (Poncet 1999). Additionally, it can inhibit the polymerization of microtubules and promote their disaggregation, interfere with the mitosis of tumor cells, and induce apoptosis of various cancer cells (Margolin et al. 2001). Monomethyl auristantin E (MMAE), a dolastatin 10 analog, is too toxic to be used alone. Hence, brentuximab vedotin, approved by the FDA in 2011 for treatment of hodgkin lymphoma and systemican aplastic large cell lymphoma, is an antibody–drug conjugate with brentuximab conjugated to the MMAE. The successful development of brentuximab vedotin undoubtedly provides a strategy for this type of natural product, for instance, symplostatin 1 (**321**), an analog of dolastatin 10 also showed excellent anticancer activity and toxicity (Fig. 6).

## Conclusions

Natural products have been contributing significantly to modern drug development. These compounds are of considerable synthetic interest as novel chemical entities for drug discovery. Encouragingly, the recent research progresses of marine natural products provide more candidates for the pharmaceutical industry (Molinski et al. 2009).

Structure–activity relationships (compounds **179**, **181**–**184**, **379**) have confirmed the key roles of the disulfide bond, thiophene ring dihydrothiophene and thiazoline in bioactivities. Take disulfide bonds as an example, because disulfide bonds have low toxicity in the body and they can be broken in the presence of reduced glutathione in the external environment, many scientists have introduced it into drugs to achieve better therapeutic effects.

**Fig. 7:**
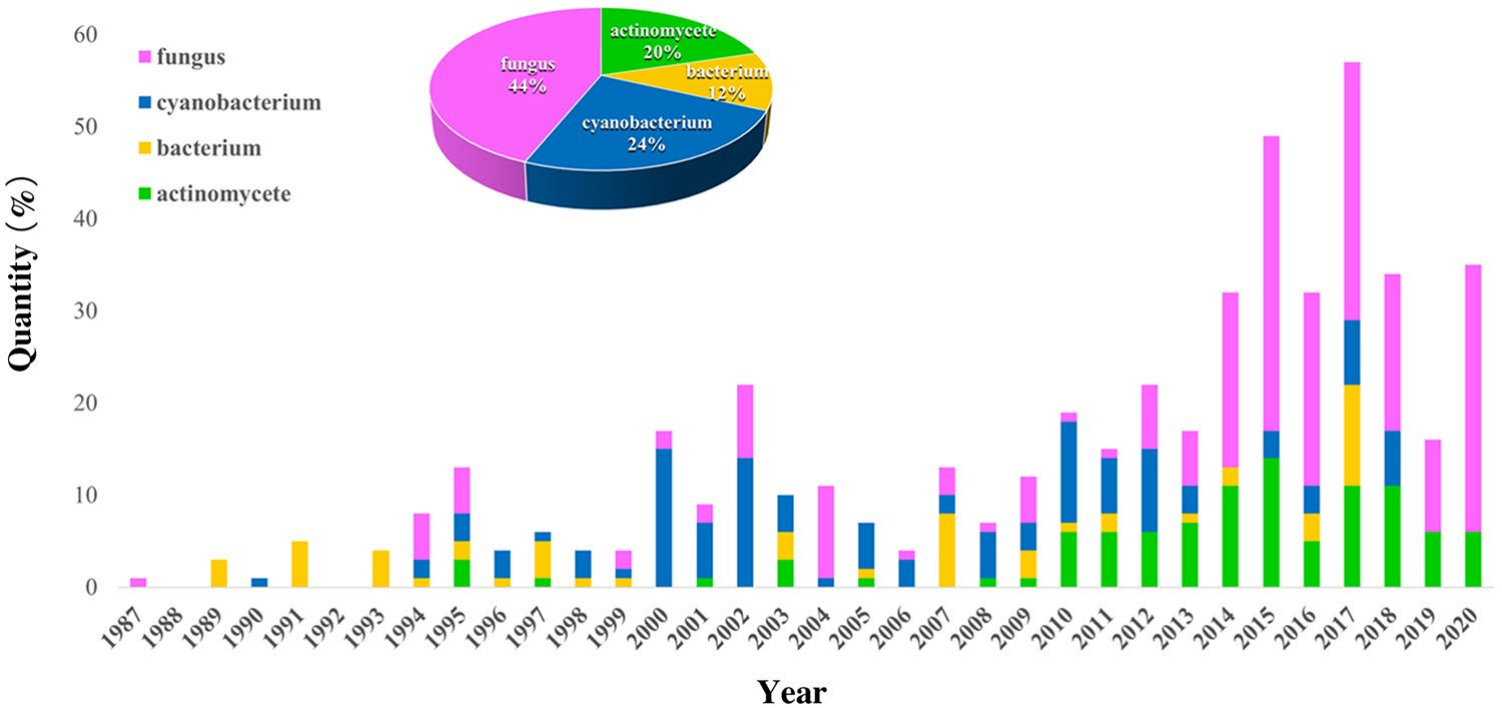
Statistics of sulfur-containing natural products (non-sulfated) from marine microorganisms from 1987 to 2020

In this review, 484 sulfur-containing natural products isolated from marine microorganisms in the period from 1987 to 2020 are categorized by their chemical structures. The isolation, biological activity, structure–activity relationships, pharmacological evaluation, biosynthesis and organic synthesis have also been summarized. Sulfur-containing MNPs have seen an impressive expansion, with a discovery rate from less than ten new compounds in the early twentieth century to more than 20 compounds per year at present. Research in the last decade has contributed more than 65% of sulfur-containing MNPs thanks to more attention (Fig. 7).

**Fig. 8:**
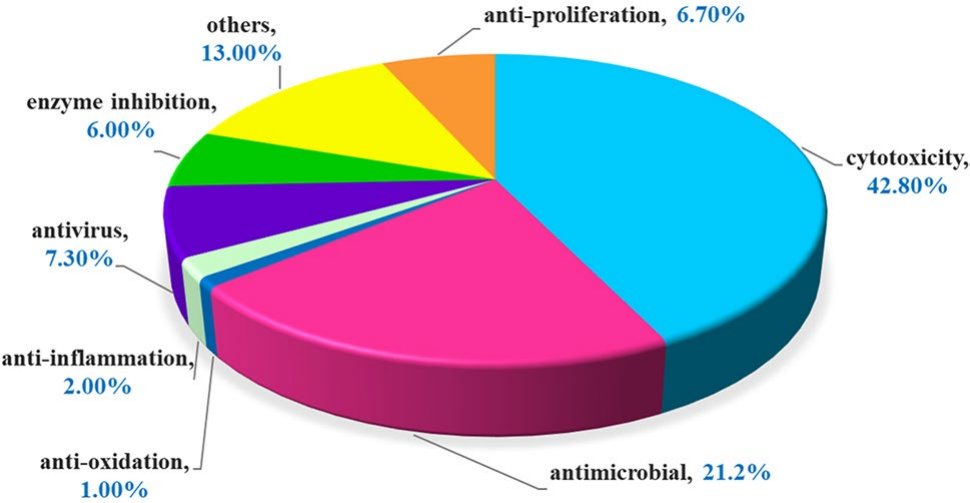
Biological activity distribution of active sulfur-containing natural products from marine microorganisms from 1987 to 2020

What is notable is that the natural products reported from fungi (43%) has increase sharply and fungi have become the most productive microbial source of sulfur-containing MNPs. The cytotoxicity (42.8%), antimicrobial (21.2%) and antivirus activities (7.3%) are the top three rankings in biological activity, followed by anti-proliferation (6.7%), enzyme inhibition (6.0%), anti-inflammation (2.0%), anti-oxidation activities (1.0%) and others (13.0%) (Fig. 8).

Abundant biological activity clearly led to an overall improvement in isolating sulfur-containing MNPs and characterization and provides optimism for drug discovery. However, most compounds are still in the discovery stage, and many potential compounds have not been further studied. The development of natural products into drugs encounters three major problems: material availability, compound druggability and therapeutic targets. Fortunately, genetic engineering may offer adequate sample amount to expand the existing research scope and allow us to explore novel lead compounds. The dereplication technologies represented by molecular networking are applied in isolation procedures (Hou et al. 2019b, c), for example, retimycin A (**223**) and hectochlorins B–D (**345**–**347**). Predicting the substructures of novel compounds has been explored. Class assignment and ontology prediction using mass spectrometry (CANOPUS) is applied to systematically classify unknown metabolites (Duhrkop et al. 2020). Simultaneously, faced with such a large number of compounds, a reliable screening and evaluation system is essential. In recent years, genetic engineering and computer aided drug design started to play an effective way to guide lead compound optimization (Kudo et al. 2020; Yu et al. 2020).

Advances in drug development technologies have provided the ability to easily solve application problems, mainly complex structures and mechanism of actions, long development processes and high capital investment. For instance, nocathiacins I–III (**364**–**366**) are expected to be developed as antibiotics to improve water solubility by modification. Kendomycins C and D (**214** and **215**), apratoxin A (**291**) and symplostatin 1 (**321**) will no longer be restricted by poor selectivity and have the opportunity to be used as anticancer drugs by structure optimization. Meanwhile, complex structures can be simplified to change physical and chemical properties, and dosage form innovation can improve bioavailability, etc. What cannot be ignored is that the biogenetic synthesis and chemical ecological role of sulfur-containing MNPs need further clarification. This is not only a demand and challenge to scientists, but it is also a problem that the pharmaceutical industry is facing. The certainty is that the interest in sulfur-containing drugs has been increasing.

The ‘golden age’ of antibiotic discovery began with microorganisms, and it is wise that we look back to the past and continue to explore untapped molecules with novel strategies. Optimistically, future research on sulfur-containing MNPs will yield even more amazing breakthroughs along with new scientific developments and methods being applied.

## Supplementary Information

Below is the link to the electronic supplementary material.

Supplementary file1 (DOCX 3276 KB)

## References

[CR1] Adams TC, Payette JN, Cheah JH, Movassaghi M (2015). Concise total synthesis of (+)-Luteoalbusins A and B. Org Lett.

[CR2] Adelin E, Martin MT, Bricot MF, Cortial S, Retailleau P, Ouazzani J (2012). Biotransformation of natural compounds: unexpected thio conjugation of Sch-642305 with 3-mercaptolactate catalyzed by *Aspergillus niger* ATCC 16404 cells. Phytochemistry.

[CR3] Asolkar RN, Freel KC, Jensen PR, Fenical W, Kondratyuk TP, Park EJ, Pezzuto JM (2009). Arenamides A–C, cytotoxic NFkappaB inhibitors from the marine actinomycete *Salinispora arenicola*. J Nat Prod.

[CR4] Bai J, Liu D, Yu SW, Proksch P, Lin WH (2014). Amicoumacins from the marine-derived bacterium *Bacillus* sp. with the inhibition of NO production. Tetrahedron Lett.

[CR5] Balk-Bindseil W, Helmke E, Weyland H, Laatsch H (1995). Marine bacteria, VIII. Maremycin A and B, new diketopiperazines from a marine *Streptomyces* sp.. Liebigs Ann.

[CR6] Balunas MJ, Linington RG, Tidgewell K, Fenner AM, Ureña L-D, Togna GD, Kyle DE, Gerwick WH (2010). Dragonamide E, a modified linear lipopeptide from *Lyngbya majuscula* with antileishmanial activity. J Nat Prod.

[CR7] Bao J, Luo JF, Qin XC, Xu XY, Zhang XY, Tu ZC, Qi SH (2014). Dihydrothiophene-condensed chromones from a marine-derived fungus *Penicillium oxalicum* and their structure-bioactivity relationship. Bioorg Med Chem Lett.

[CR8] Bastians H, Schwab M (2011). UCN-01 anticancer drug. Encyclopedia of cancer.

[CR9] Baunach M, Ding L, Willing K, Hertweck C (2015). Bacterial synthesis of unusual sulfonamide and sulfone antibiotics by flavoenzyme–mediated sulfur dioxide capture. Angew Chem Int Ed.

[CR10] Belisle RS, Via CW, Schock TB, Villareal TA, Zimba PV, Beauchesne KR, Moeller PDR, Bertin MJ (2017). Trichothiazole A, a dichlorinated polyketide containing an embedded thiazole isolated from *Trichodesmium* blooms. Tetrahedron Lett.

[CR11] Bell MR, Johnson JR, Wildi BS, Woodward RB (1958). The structure of gliotoxin. J Am Chem Soc.

[CR12] Berman FW, Gerwick WH, Murray TF (1999). Antillatoxin and kalkitoxin, ichthyotoxins from the tropical cyanobacterium *Lyngbya majuscula*, induce distinct temporal patterns of NMDA receptor–mediated neurotoxicity. Toxicon.

[CR13] Bharate SB, Sawant SD, Singh PP, Vishwakarma RA (2013). Kinase inhibitors of marine origin. Chem Rev.

[CR14] Blokhin AV, Yoo HD, Geralds RS, Nagle DG, Gerwick WH, Hamel E (1995). Characterization of the interaction of the marine cyanobacterial natural product curacin A with the colchicine site of tubulin and initial structure-activity studies with analogues. Mol Pharmacol.

[CR15] Boger DL, Ichikawa S (2000). Total syntheses of thiocoraline and BE–22179: establishment of relative and absolute stereochemistry. J Am Chem Soc.

[CR16] Boger DL, Ichikawa S, Tse WC, Hedrick MP, Jin Q (2001). Total syntheses of thiocoraline and BE-22179 and assessment of their dna binding and biological properties. J Am Chem Soc.

[CR17] Bohonos N, Chou TW, Spanggord RJ (1977). Some observations on biodegradation of pollutants in aquatic systems. Jpn J Antibiot.

[CR18] Bose AK, Khanchandani KS, Tavares R, Funke PT (1968). Biosynthetic studies. II. The mode of incorporation of phenylalanine into gliotoxin. J Am Chem Soc.

[CR19] Boudreau PD, Monroe EA, Mehrotra S, Desfor S, Korobeynikov A, Sherman DH, Murray TF, Gerwick L, Dorrestein PC, Gerwick WH (2015). Expanding the described metabolome of the marine cyanobacterium *Moorea producens* JHB through orthogonal natural products workflows. PLoS ONE.

[CR20] Boyer N, Movassaghi M (2012). Concise total synthesis of (+)-gliocladins B and C. Chem Sci.

[CR21] Bray C, Olasoji J (2009). A total synthesis of (+)-bacillamide B. Synlett.

[CR22] Bu YY, Yamazaki H, Ukai K, Namikoshi M (2014). Anti–mycobacterial nucleoside antibiotics from a marine–derived *Streptomyces* sp. TPU1236A. Mar Drugs.

[CR23] Burckhardt T, Harms K, Koert U (2012). Total synthesis of lodopyridone. Org Lett.

[CR24] Cañedo LM, de la Fuente JA, Gesto C, Ferreiro MJ, Jiménez C, Riguera R (1999). Agrochelin, a new cytotoxic alkaloid from the marine bacteria *Agrobacterium* sp. Tetrahedron Lett.

[CR25] Cao DT, Tran VH, Vu VN, Mai HDT, Le THM, Vu TQ, Nguyen HH, Chau VM, Pham VC (2019). Antimicrobial metabolites from a marine-derived Actinomycete *Streptomyces* sp. G278. Nat Prod Res.

[CR26] Cao J, Li XM, Meng LH, Konuklugil B, Li X, Li HL, Wang BG (2019). Isolation and characterization of three pairs of indolediketopiperazine enantiomers containing infrequent *N*-methoxy substitution from the marine algal-derived endophytic fungus *Acrostalagmus luteoalbus* TK–43. Bioorg Chem.

[CR27] Capon RJ, Stewart M, Ratnayake R, Lacey E, Gill JH (2007). Citromycetins and Bilains A–C: new aromatic polyketides and diketopiperazines from australian marine-derived and terrestrial *Penicillium* spp.. J Nat Prod.

[CR28] Carr G, Tay W, Bottriell H, Andersen SK, Mauk AG, Andersen RJ (2009). Plectosphaeroic acids A, B, and C, indoleamine 2,3-dioxygenase inhibitors produced in culture by a marine isolate of the fungus *Plectosphaerella cucumerina*. Org Lett.

[CR29] Carroll AR, Copp BR, Davis RA, Keyzers RA, Prinsep MR (2019). Marine natural products. Nat Prod Rep.

[CR30] Carroll AR, Copp BR, Davis RA, Keyzers RA, Prinsep MR (2020). Marine natural products. Nat Prod Rep.

[CR31] Chan AN, Shiver AL, Wever WJ, Razvi SZ, Traxler MF, Li B (2017). Role for dithiolopyrrolones in disrupting bacterial metal homeostasis. Proc Natl Acad Sci USA.

[CR32] Chang YM, Xing L, Sun CX, Liang S, Liu T, Zhang XM, Zhu TJ, Pfeifer BA, Che Q, Zhang GJ, Li DH (2020). Monacycliones G-K and ent-gephyromycin A, angucycline derivatives from the marine-derived *Streptomyces* sp. HDN15129. J Nat Prod.

[CR33] Che Q, Tan HS, Han XN, Zhang XM, Gu QQ, Zhu TJ, Li DH (2016). Naquihexcin A, a S-Bridged pyranonaphthoquinone dimer bearing an unsaturated hexuronic acid moiety from a sponge–derived *Streptomyces* sp. HDN–10–293. Org Lett.

[CR34] Chen Y, Zhang YX, Li MH, Zhao WM, Shi YH, Miao ZH, Zhang XW, Lin LP, Ding J (2005). Antiangiogenic activity of 11,11′-dideoxyverticillin, a natural product isolated from the fungus *Shiraia bambusicola*. Biochem Biophys Res Commun.

[CR35] Chen QY, Liu Y, Luesch H (2011). Systematic chemical mutagenesis identifies a potent novel apratoxin A/E hybrid with improved in vivo antitumor activity. ACS Med Chem Lett.

[CR36] Chen M, Shao CL, Fu XM, Kong CJ, She ZG, Wang CY (2014). Lumazine peptides penilumamides B–D and the cyclic pentapeptide asperpeptide A from a gorgonian–derived *Aspergillus* sp. fungus. J Nat Prod.

[CR37] Chen L, Chai WY, Wang WL, Song TF, Lian XY, Zhang Z (2017). Cytotoxic bagremycins from mangrove–derived *Streptomyces* sp. Q22. J Nat Prod.

[CR38] Chen YX, Xu MY, Li HJ, Zeng KJ, Ma WZ, Tian GB, Xu J, Yang DP, Lan WJ (2017). Diverse secondary metabolites from the marine-derived fungus *Dichotomomyces cejpii* F31-1. Mar Drugs.

[CR39] Chen YP, Chen RY, Xu JH, Tian YQ, Xu JP, Liu YH (2018). Two new altenusin/thiazole hybrids and a new benzothiazole derivative from the marine sponge-derived fungus *Alternaria* sp. SCSIOS02F49. Molecules.

[CR40] Chi LP, Li XM, Li L, Li X, Wang BG (2020). Cytotoxic thiodiketopiperazine derivatives from the deep sea-derived fungus *Epicoccum nigrum* SD-388. Mar Drugs.

[CR41] Chi LP, Li XM, Li X, Wang BG (2020). New antibacterial thiodiketopiperazines from the deep sea sediment-derived fungus *Epicoccum nigrum* SD-388. Chem Biodivers.

[CR42] Choi H, Pereira AR, Cao Z, Shuman CF, Engene N, Byrum T, Matainaho T, Murray TF, Mangoni A, Gerwick WH (2010). The hoiamides, structurally intriguing neurotoxic lipopeptides from Papua New Guinea marine cyanobacteria. J Nat Prod.

[CR43] Choi EJ, Park JS, Kim YJ, Jung JH, Lee JK, Kwon HC, Yang HO (2011). Apoptosis-inducing effect of diketopiperazine disulfides produced by *Aspergillus* sp. KMD 901 isolated from marine sediment on HCT116 colon cancer cell lines. J Appl Microbiol.

[CR44] Choi H, Mevers E, Byrum T, Valeriote FA, Gerwick WH (2012). Lyngbyabellins K-N from two palmyra atoll collections of the marine cyanobacterium *Moorea bouillonii*. Eur J Org Chem.

[CR45] Christophersen C (1989). Biologically active sulfur compounds from marine organisms. Phosphorus Sulfur.

[CR46] Christophersen C, Anthoni U (1986). Organic sulfur compounds from marine organisms. Sulfur Rep.

[CR47] Ciminiello P, Dell'Aversano C, Fattorusso E, Forino M, Magno S, Di Rosa M, Ianaro A, Poletti R (2002). Structure and stereochemistry of a new cytotoxic polychlorinated sulfolipid from adriatic shellfish. J Am Chem Soc.

[CR48] Ciomei M, Croci V, Ciavolella A, Ballinari D, Pesenti E (2006). Antitumor efficacy of edotecarin as a single agent and in combination with chemotherapy agents in a xenograft model. Clin Cancer Res.

[CR49] Cramer RA, Gamcsik MP, Brooking RM, Najvar LK, Kirkpatrick WR, Patterson TF, Balibar CJ, Graybill JR, Perfect JR, Abraham SN, Steinbach WJ (2006). Disruption of a nonribosomal peptide synthetase in *Aspergillus fumigatus* eliminates gliotoxin production. Eukaryot Cell.

[CR50] Dai JJ, Chen A, Zhu ML, Qi X, Tang W, Liu M, Li DH, Gu QQ, Li J (2019). Penicisulfuranol A, a novel C-terminal inhibitor disrupting molecular chaperone function of Hsp90 independent of ATP binding domain. Biochem Pharmacol.

[CR51] DeLorbe JE, Horne D, Jove R, Mennen SM, Nam S, Zhang FL, Overman LE (2013). General approach for preparing epidithiodioxopiperazines from trioxopiperazine precursors: enantioselective total syntheses of (+)-and (−)-gliocladine C, (+)-leptosin D, (+)-T988C, (+)-bionectin A, and (+)-gliocladin A. J Am Chem Soc.

[CR52] Deng CM, Liu SX, Huang CH, Pang JY, Lin YC (2013). Secondary metabolites of a mangrove endophytic fungus *Aspergillus terreus* (No. GX7-3B) from the South China Sea. Mar Drugs.

[CR53] Deng JJ, Lu CH, Li SR, Hao HL, Li ZY, Zhu J, Li YY, Shen YM (2014). *p*–Terphenyl *O*-*β*-glucuronides, DNA topoisomerase inhibitors from *Streptomyces* sp. LZ35ΔgdmAI. Bioorg Med Chem Lett.

[CR54] Dickschat JS, Reichenbach H, Wagner-Döbler I, Schulz S (2005). Novel pyrazines from the myxobacterium *Chondromyces crocatusand* marine bacteria. Eur J Org Chem.

[CR55] Diettrich J, Kage H, Nett M (2019). Genomics-inspired discovery of massiliachelin, an agrochelin epimer from *Massilia* sp. NR 4–1. Beilstein J Org Chem.

[CR56] Ding LJ, Yuan W, Liao XJ, Han BN, Wang SP, Li ZY, Xu SH, Zhang W, Lin HW (2016). Oryzamides A-E, cyclodepsipeptides from the sponge-derived fungus *Nigrospora oryzae* PF18. J Nat Prod.

[CR57] Ding H, Wang JN, Zhang DS, Ma ZJ (2017). Derivatives of holomycin and cyclopropaneacetic acid from *Streptomyces* sp. DT–A37. Chem Biodivers.

[CR58] Duhrkop K, Nothias LF, Fleischauer M, Reher R, Ludwig M, Hoffmann MA, Petras D, Gerwick WH, Rousu J, Dorrestein PC, Bocker S (2021). Systematic classification of unknown metabolites using high-resolution fragmentation mass spectra. Nat Biotechnol.

[CR59] Duncan KR, Crusemann M, Lechner A, Sarkar A, Li J, Ziemert N, Wang M, Bandeira N, Moore BS, Dorrestein PC, Jensen PR (2015). Molecular networking and pattern-based genome mining improves discovery of biosynthetic gene clusters and their products from *Salinispora* species. Chem Biol.

[CR60] Elnaggar MS, Ebrahim W, Mándi A, Kurtán T, Müller WEG, Kalscheuer R, Singab A, Lin WH, Liu Z, Proksch P (2017). Hydroquinone derivatives from the marine-derived fungus *Gliomastix* sp. RSC Adv.

[CR61] Engelhardt K, Degnes KF, Kemmler M, Bredholt H, Fjaervik E, Klinkenberg G, Sletta H, Ellingsen TE, Zotchev SB (2010). Production of a new thiopeptide antibiotic, TP–1161, by a marine *Nocardiopsis* species. Appl Environ Microbiol.

[CR62] Engelhardt K, Degnes KF, Zotchev SB (2010). Isolation and characterization of the gene cluster for biosynthesis of the thiopeptide antibiotic TP-1161. Appl Environ Microbiol.

[CR63] Faircloth G, Jimeno J, D’lncalci M (1997). 781—biological activity of thiocoraline, a novel marine depsipeptide. Eur J Cancer.

[CR64] Feng YJ, Blunt JW, Cole ALJ, Munro MHG (2004). Novel cytotoxic thiodiketopiperazine derivatives from a *Tilachlidium* sp.. J Nat Prod.

[CR65] Ferezou J-P, Quesneau-Thierry A, Servy C, Zissmann E, Barbier M (1980). Sirodesmin PL biosynthesis in *Phoma lingam* tode, Perkin Trans. J Chem Soc.

[CR66] Flatt PM, O'Connell SJ, McPhail KL, Zeller G, Willis CL, Sherman DH, Gerwick WH (2006). Characterization of the initial enzymatic steps of barbamide biosynthesis. J Nat Prod.

[CR67] Fleming A (1929). The antibacterial action of cultures of a *Penicillium*, with special reference to their use in the isolation of *B. influenzae*. Br J Exp Pathol.

[CR68] Fridrichsons J, McL Mathieson A (1967). The crystal structure of gliotoxin. Acta Crystallogr.

[CR69] Friedländer P (1909). Über den Farbstoff des antiken purpurs aus murex brandaris. Ber Dtsch Chem Ges.

[CR70] Fu P, MacMillan JB (2015). Spithioneines A and B, two new bohemamine derivatives possessing ergothioneine moiety from a marine-derived *Streptomyces spinoverrucosus*. Org Lett.

[CR71] Fu P, MacMillan JB (2015). Thiasporines A–C, thiazine and thiazole derivatives from a marine-derived *Actinomycetospora chlora*. J Nat Prod.

[CR72] Fu P, Zhuang YB, Wang Y, Liu PP, Qi X, Gu KB, Zhang DJ, Zhu WM (2012). New indolocarbazoles from a mutant strain of the marine-derived actinomycete *Streptomyces fradiae* 007M135. Org Lett.

[CR73] Fukuda T, Nagai K, Kurihara Y, Kanamoto A, Tomoda H (2015). Graphiumins I and J, new thiodiketopiperazines from the marine-derived fungus *Graphium* sp. OPMF00224. Nat Prod Sci.

[CR74] Fukuda T, Shinkai M, Sasaki E, Nagai K, Kurihara Y, Kanamoto A, Tomoda H (2015). Graphiumins, new thiodiketopiperazines from the marine-derived fungus *Graphium* sp. OPMF00224. J Antibiot (Tokyo).

[CR75] Galonić DP, Vaillancourt FH, Walsh CT (2006). Halogenation of unactivated carbon centers in natural product biosynthesis: trichlorination of leucine during barbamide biosynthesis. J Am Chem Soc.

[CR76] Gao XG, Liu YQ, Kwong S, Xu ZS, Ye T (2010). Total synthesis and stereochemical reassignment of bisebromoamide. Org Lett.

[CR77] Gao XW, Liu HX, Sun ZH, Chen YC, Tan YZ, Zhang WM (2016). Secondary metabolites from the deep-sea derived fungus *Acaromyces ingoldii* FS121. Molecules.

[CR78] Gao SS, Wang LY, Song ZS, Hothersall J, Stevens ER, Connolly J, Winn PJ, Cox RJ, Crump MP, Race PR, Thomas CM, Simpson TJ, Willis CL (2017). Selected mutations reveal new intermediates in the biosynthesis of mupirocin and the thiomarinol antibiotics. Angew Chem Int Ed.

[CR79] Gardiner DM, Waring P, Howlett BJ (2005). The epipolythiodioxopiperazine (ETP) class of fungal toxins: distribution, mode of action, functions and biosynthesis. Microbiology.

[CR80] Gerwick WH, Moore BS (2012). Lessons from the past and charting the future of marine natural products drug discovery and chemical biology. Chem Biol.

[CR81] Gerwick WH, Proteau PJ, Nagle DG, Hamel E, Blokhin A, Slate DL (1994). Structure of curacin A, a novel antimitotic, antiproliferative and brine shrimp toxic natural product from the marine cyanobacterium *Lyngbya majuscula*. J Org Chem.

[CR82] Gerwick WH, Tong Tan L, Sitachitta N (2001). Nitrogen-containing metabolites from marine cyanobacteria. Alkaloids Chem Biol.

[CR83] González J, Gerwick WH (2007). Venturamides A and B: antimalarial constituents of the panamanian marine cyanobacterium *Oscillatoria* sp. J Nat Prod.

[CR84] Gu BB, Zhang YY, Ding LJ, He S, Wu B, Dong JD, Zhu P, Chen JJ, Zhang JR, Yan XJ (2015). Preparative separation of sulfur-containing diketopiperazines from marine fungus *Cladosporium* sp. using high-speed counter-current chromatography in stepwise elution mode. Mar Drugs.

[CR85] Gunasekera SP, Ritson-Williams R, Paul VJ (2008). Carriebowmide, a new cyclodepsipeptide from the marine cyanobacterium *Lyngbya polychroa*. J Nat Prod.

[CR86] Gutiérrez M, Suyama TL, Engene N, Wingerd JS, Matainaho T, Gerwick WH (2008). Apratoxin D, a potent cytotoxic cyclodepsipeptide from papua new guinea collections of the marine cyanobacteria *Lyngbya majuscula* and *Lyngbya sordida*. J Nat Prod.

[CR87] Hammons JC, Trzoss L, Jimenez PC, Hirata AS, Costa-Lotufo LV, La Clair JJ, Fenical W (2019). Advance of seriniquinone analogues as melanoma agents. ACS Med Chem Lett.

[CR88] Han BN, McPhail KL, Gross H, Goeger DE, Mooberry SL, Gerwick WH (2005). Isolation and structure of five lyngbyabellin derivatives from a Papua New Guinea collection of the marine cyanobacterium *Lyngbya majuscula*. Tetrahedron.

[CR89] Han X, Li P, Luo X, Qiao D, Tang X, Li G (2019). Two new compounds from the marine sponge derived fungus *Penicillium chrysogenum*. Nat Prod Res.

[CR90] Harig T, Schlawis C, Ziesche L, Pohlner M, Engelen B, Schulz S (2017). Nitrogen-containing volatiles from marine *Salinispora pacifica* and *Roseobacter*-group bacteria. J Nat Prod.

[CR91] Harms H, Orlikova B, Ji S, Nesaei-Mosaferan D, Konig GM, Diederich M (2015). Epipolythiodiketopiperazines from the marine derived fungus *Dichotomomyces cejpii* with NF-kappaB inhibitory potential. Mar Drugs.

[CR92] Harrigan GG, Luesch H, Yoshida WY, Moore RE, Nagle DG, Paul VJ, Mooberry SL, Corbett TH, Valeriote FA (1998). Symplostatin 1: a dolastatin 10 analogue from the marine cyanobacterium *Symploca hydnoides*. J Nat Prod.

[CR93] Harrigan GG, Luesch H, Yoshida WY, Moore RE, Nagle DG, Paul VJ (1999). Symplostatin 2: a dolastatin 13 analogue from the marine cyanobacterium *Symploca hydnoides*. J Nat Prod.

[CR94] Horton P, Inman WD, Crews P (1990). Enantiomeric relationships and anthelmintic activity of dysinin derivatives from *Dysidea* marine sponges. J Nat Prod.

[CR95] Hou XM, Xu RF, Gu YC, Wang CY, Shao CL (2015). Biological and chemical diversity of coral–derived microorganisms. Curr Med Chem.

[CR96] Hou XM, Hai Y, Gu YC, Wang CY, Shao CL (2019). Chemical and bioactive marine natural products of coral-derived microorganisms (2015–2017). Curr Med Chem.

[CR97] Hou XM, Li YY, Shi YW, Fang YW, Chao R, Gu YC, Wang CY, Shao CL (2019). Integrating molecular networking and ^1^H NMR to target the isolation of chrysogeamides from a library of marine-derived *Penicillium* fungi. J Org Chem.

[CR98] Hou XM, Liang TM, Guo ZY, Wang CY, Shao CL (2019). Discovery, absolute assignments, and total synthesis of asperversiamides A–C and their potent activity against *Mycobacterium marinum*. Chem Commun (Camb).

[CR99] Hu Y, MacMillan JB (2011). Erythrazoles A-B, cytotoxic benzothiazoles from a marine-derived *Erythrobacter* sp.. Org Lett.

[CR100] Huang LH, Xu MY, Li HJ, Li JQ, Chen YX, Ma WZ, Li YP, Xu J, Yang DP, Lan WJ (2017). Amino acid-directed strategy for inducing the marine-derived fungus *Scedosporium apiospermum* F41-1 to maximize alkaloid diversity. Org Lett.

[CR101] Huang HB, Song YX, Li X, Wang X, Ling CY, Qin XJ, Zhou ZB, Li QL, Wei X, Ju JH (2018). Abyssomicin monomers and dimers from the marine-derived *Streptomyces koyangensis* SCSIO 5802. J Nat Prod.

[CR102] Hughes CC, MacMillan JB, Gaudencio SP, Jensen PR, Fenical W (2009). The ammosamides: structures of cell cycle modulators from a marine-derived *Streptomyces* species. Angew Chem Int Ed.

[CR103] Ilardi EA, Vitaku E, Njardarson JT (2014). Data-mining for sulfur and fluorine: an evaluation of pharmaceuticals to reveal opportunities for drug design and discovery. J Med Chem.

[CR104] Iwasaki A, Tadenuma T, Sumimoto S, Ohshiro T, Ozaki K, Kobayashi K, Teruya T, Tomoda H, Suenaga K (2017). Biseokeaniamides A, B, and C, sterol *O*-acyltransferase inhibitors from an *Okeania* sp. marine cyanobacterium. J Nat Prod.

[CR105] Jabri SY, Overman LE (2013). Enantioselective total syntheses of plectosphaeroic acids B and C. J Org Chem.

[CR106] Jadulco R, Proksch P, Wray V, Sudarsono BA, Gräfe U (2001). New macrolides and furan carboxylic acid derivative from the sponge-derived fungus *Cladosporium herbarum*. J Nat Prod.

[CR107] Jalal MAF, Hossain MB, Van der Helm D, Sanders-Loehr J, Actis LA, Crosa JH (1989). Structure of anguibactin, a unique plasmid-related bacterial siderophore from the fish pathogen *Vibrio anguillarum*. J Am Chem Soc.

[CR108] Jayatilake GS, Thornton MP, Leonard AC, Grimwade JE, Baker BJ (1996). Metabolites from an antarctic sponge-associated bacterium, *Pseudomonas aeruginosa*. J Nat Prod.

[CR109] Jeong S-Y, Ishida K, Ito Y, Okada S, Murakami M (2003). Bacillamide, a novel algicide from the marine bacterium, *Bacillus* sp. SY-1, against the harmful dinoflagellate, *Cochlodinium polykrikoides*. Tetrahedron Lett.

[CR110] Jiang CS, Guo YW (2011). Epipolythiodioxopiperazines from fungi: chemistry and bioactivities. Mini Rev Med Chem.

[CR111] Jiménez JI, Scheuer PJ (2001). New lipopeptides from the Caribbean Cyanobacterium *Lyngbya majuscula*. J Nat Prod.

[CR112] Jiménez JI, Vansach T, Yoshida WY, Sakamoto B, Pörzgen P, Horgen FD (2009). Halogenated fatty acid amides and cyclic depsipeptides from an eastern caribbean collection of the cyanobacterium *Lyngbya majuscula*. J Nat Prod.

[CR113] Jimenez C (2018). Marine natural products in medicinal chemistry. ACS Med Chem Lett.

[CR114] Jo MJ, Patil MP, Jung HI, Seo YB, Lim HK, Son BW, Kim GD (2019). Cristazine, a novel dioxopiperazine alkaloid, induces apoptosis via the death receptor pathway in A431 cells. Drug Dev Res.

[CR115] Johnson JR, Bruce WF, Dutcher JD (1943). Gliotoxin, the antibiotic principle of *Gliocladium fimbriatum*. I. production, physical and biological properties^1^. J Am Chem Soc.

[CR116] Kanoh K, Matsuo Y, Adachi K, Imagawa H, Nishizawa M, Shizuri Y (2005). Mechercharmycins A and B, cytotoxic substances from marine-derived *Thermoactinomyces* sp. YM3–251. J Antibiot.

[CR117] Kanoh K, Matsuo Y, Adachi K, Imagawa H, Nishizawa M, Shizuri Y (2007). Corrections. J Antibiot.

[CR118] Kawahara T, Izumikawa M, Kozone I, Hashimoto J, Kagaya N, Koiwai H, Komatsu M, Fujie M, Sato N, Ikeda H, Shin–Ya K, (2018). Neothioviridamide, a polythioamide compound produced by heterologous expression of a *Streptomyces* sp. cryptic ripp biosynthetic gene cluster. J Nat Prod.

[CR119] Khodamoradi S, Stadler M, Wink J, Surup F (2020). Litoralimycins A and B, new cytotoxic thiopeptides from *Streptomonospora* sp. M2. Mar Drugs.

[CR120] Kim ES (2017). Midostaurin: first global approval. Drugs.

[CR121] Kim J, Ashenhurst JA, Movassaghi M (2009). Total synthesis of (+)–11,11'-dideoxyverticillin A. Science.

[CR122] Kim EJ, Lee JH, Choi H, Pereira AR, Ban YH, Yoo YJ, Kim E, Park JW, Sherman DH, Gerwick WH, Yoon YJ (2012). Heterologous production of 4-*O*-Demethylbarbamide, a marine cyanobacterial natural product. Org Lett.

[CR123] Kim H, Yang I, Patil RS, Kang S, Lee J, Choi H, Kim MS, Nam SJ, Kang H (2014). Anithiactins A–C, modified 2-phenylthiazoles from a mudflat-derived *Streptomyces* sp.. J Nat Prod.

[CR124] Kirby GW, Patrick GL, Robins DJ (1978). *Cyclo*-(*L*-Phenylalanyl-*L*-seryl) as an intermediate in the biosynthesis of gliotoxin. J Chem Soc Perkin Trans.

[CR125] Klein D, Braekman JC, Daloze D, Hoffmann L, Castillo G, Demoulin V (1999). Lyngbyapeptin A, a modified tetrapeptide from *Lyngbya bouillonii* (Cyanophyceae). Tetrahedron Lett.

[CR126] Kong D, Park EJ, Stephen AG, Calvani M, Cardellina JH, Monks A, Fisher RJ, Shoemaker RH, Melillo G (2005). Echinomycin, a small-molecule inhibitor of hypoxia-inducible factor-1 DNA-binding activity. Cancer Res.

[CR127] Kong FD, Wang Y, Liu PP, Dong TH, Zhu WM (2014). Thiodiketopiperazines from the marine-derived fungus *Phoma* sp. OUCMDZ–1847. J Nat Prod.

[CR128] Kong FD, Zhang SL, Zhou SQ, Ma QY, Xie QY, Chen JP, Li JH, Zhou LM, Yuan JZ, Hu Z, Dai HF, Huang XL, Zhao YX (2019). Quinazoline-containing indole alkaloids from the marine-derived fungus *Aspergillus* sp. HNMF114. J Nat Prod.

[CR129] Kudo K, Hashimoto T, Hashimoto J, Kozone I, Kagaya N, Ueoka R, Nishimura T, Komatsu M, Suenaga H, Ikeda H, Shin-ya K (2020). In vitro Cas9-assisted editing of modular polyketide synthase genes to produce desired natural product derivatives. Nat Commun.

[CR130] Kwan JC, Rocca JR, Abboud KA, Paul VJ, Luesch H (2008). Total structure determination of grassypeptolide, a new marine cyanobacterial cytotoxin. Org Lett.

[CR131] Kwan JC, Ratnayake R, Abboud KA, Paul VJ, Luesch H (2010). Grassypeptolides A–C, cytotoxic bis-thiazoline containing marine cyclodepsipeptides. J Org Chem.

[CR132] Kyeremeh K, Acquah KS, Sazak A, Houssen W, Tabudravu J, Deng H, Jaspars M (2014). Butremycin, the 3-hydroxyl derivative of ikarugamycin and a protonated aromatic tautomer of 5'-methylthioinosine from a Ghanaian *Micromonospora* sp. K310. Mar Drugs.

[CR133] Lan WJ, Wang KT, Xu MY, Zhang JJ, Lam CK, Zhong GH, Xu J, Yang DP, Li HJ, Wang LY (2016). Secondary metabolites with chemical diversity from the marine-derived fungus *Pseudallescheria boydii* F19-1 and their cytotoxic activity. RSC Adv.

[CR134] Lane AL, Nam SJ, Fukuda T, Yamanaka K, Kauffman CA, Jensen PR, Fenical W, Moore BS (2013). Structures and comparative characterization of biosynthetic gene clusters for cyanosporasides, enediyne-derived natural products from marine actinomycetes. J Am Chem Soc.

[CR135] Le TC, Yim CY, Park S, Katila N, Yang I, Song MC, Yoon YJ, Choi DY, Choi H, Nam SJ, Fenical W (2017). Lodopyridones B and C from a marine sediment-derived bacterium *Saccharomonospora* sp. Bioorg Med Chem Lett.

[CR136] Lee H, Song WY, Kim M, Lee MW, Kim S, Park YS, Kwak K, Oh MH, Kim HJ (2018). Synthesis and characterization of anguibactin to reveal its competence to function as a thermally stable surrogate siderophore for a gram-negative pathogen, *Acinetobacter baumannii*. Org Lett.

[CR137] Lee J, Gamage CDB, Kim GJ, Hillman PF, Lee C, Lee EY, Choi H, Kim H, Nam SJ, Fenical W (2020). Androsamide, a cyclic tetrapeptide from a marine *Nocardiopsis* sp., suppresses motility of colorectal cancer cells. J Nat Prod.

[CR139] Leet JE, Li WY, Helen AAX, Matson JA, Huang S, Huang R, Cantone JL, Drexler D, Dalterio RA, Lam KS (2003). Nocathiacins, new thiazolyl peptide antibiotics from *Nocardia* sp. II.. J Antibiot.

[CR140] Li XF, Kim SK, Nam KW, Kang JS, Choi HD, Son BW (2006). A new antibacterial dioxopiperazine alkaloid related to gliotoxin from a marine isolate of the fungus *Pseudallescheria*. J Antibiot.

[CR141] Li CY, Ding WJ, Shao CL, She ZG, Lin YC (2008). Secondary metabolites of a marine mangrove fungus (*Penicillium* sp. no. 2556) from South China Sea. J Chin Med Mater.

[CR142] Li WH, Yu SY, Jin MZ, Xia HG, Ma DW (2011). Total synthesis and cytotoxicity of bisebromoamide and its analogues. Tetrahedron Lett.

[CR143] Li F, Guo WQ, Wu L, Zhu TJ, Gu QQ, Li DH, Che Q (2018). Saroclazines A–C, thio-diketopiperazines from mangrove–derived fungi *Sarocladium kiliense* HDN11–84. Arch Pharm Res.

[CR144] Li W, Li XB, Lou HX (2018). Structural and biological diversity of natural *p*-terphenyls. J Asian Nat Prod Res.

[CR145] Li MP, Xu YC, Zuo MX, Liu W, Wang LP, Zhu WM (2019). Semisynthetic derivatives of fradcarbazole A and their cytotoxicity against acute myeloid leukemia cell lines. J Nat Prod.

[CR146] Li CJ, Chen PN, Li HJ, Mahmud T, Wu DL, Xu J, Lan WJ (2020). Potential antidiabetic fumiquinazoline alkaloids from the marine-derived fungus *Scedosporium apiospermum* F41-1. J Nat Prod.

[CR147] Liang WL, Le X, Li HJ, Yang XL, Chen JX, Xu J, Liu HL, Wang LY, Wang KT, Hu KC, Yang DP, Lan WJ (2014). Exploring the chemodiversity and biological activities of the secondary metabolites from the marine fungus *Neosartorya pseudofischeri*. Mar Drugs.

[CR148] Lin Z, Antemano RR, Hughen RW, Tianero MD, Peraud O, Haygood MG, Concepcion GP, Olivera BM, Light A, Schmidt EW (2010). Pulicatins A–E, neuroactive thiazoline metabolites from cone snail-associated bacteria. J Nat Prod.

[CR149] Lin CC, Tantisantisom W, McAlpine SR (2013). Total synthesis and biological activity of natural product urukthapelstatin A. Org Lett.

[CR150] Lin ZJ, Smith MD, Concepcion GP, Haygood MG, Olivera BM, Light A, Schmidt EW (2017). Modulating the serotonin receptor spectrum of pulicatin natural products. J Nat Prod.

[CR151] Liu Y, Law BK, Luesch H (2009). Apratoxin a reversibly inhibits the secretory pathway by preventing cotranslational translocation. Mol Pharmacol.

[CR152] Liu H, Liu Y, Xing X, Xu Z, Ye T (2010). Total synthesis of grassypeptolide. Chem Commun (Camb).

[CR153] Liu N, Shang F, Xi L, Huang Y (2013). Tetroazolemycins A and B, two new oxazole-thiazole siderophores from deep-sea *Streptomyces olivaceus* FXJ8.012. Mar Drugs.

[CR154] Liu Z, Xia G, Chen S, Liu Y, Li H, She Z (2014). Eurothiocin A and B, sulfur-containing benzofurans from a soft coral-derived fungus *Eurotium rubrum* SH-823. Mar Drugs.

[CR155] Liu W, Li HJ, Xu MY, Ju YC, Wang LY, Xu J, Yang DP, Lan WJ (2015). Pseudellones A–C, three alkaloids from the marine-derived fungus *Pseudallescheria ellipsoidea* F42-3. Org Lett.

[CR156] Liu Y, Li XM, Meng LH, Jiang WL, Xu GM, Huang CG, Wang BG (2015). Bisthiodiketopiperazines and acorane sesquiterpenes produced by the marine-derived fungus *Penicillium adametzioides* AS-53 on different culture media. J Nat Prod.

[CR157] Liu Y, Mandi A, Li XM, Meng LH, Kurtan T, Wang BG (2015). Peniciadametizine A, a dithiodiketopiperazine with a unique spiro[furan-2,7'-pyrazino[1,2-b][1,2]oxazine] skeleton, and a related analogue, peniciadametizine B, from the marine sponge–derived fungus *Penicillium adametzioides*. Mar Drugs.

[CR158] Liu CC, Zhang ZZ, Feng YY, Gu QQ, Li DH, Zhu TJ (2019). Secondary metabolites from Antarctic marine-derived fungus *Penicillium crustosum* HDN153086. Nat Prod Res.

[CR159] Liu L, Zheng YY, Shao CL, Wang CY (2019). Metabolites from marine invertebrates and their symbiotic microorganisms: molecular diversity discovery, mining, and application. Mar Life Sci Technol.

[CR160] Lorig-Roach N, Still PC, Coppage D, Compton JE, Crews MS, Navarro G, Tenney K, Crews P (2017). Evaluating nitrogen-containing biosynthetic products produced by saltwater culturing of several california littoral zone gram-negative bacteria. J Nat Prod.

[CR161] Luesch H, Yoshida WY, Moore RE, Paul VJ (2000). Apramides A−G, novel lipopeptides from the marine cyanobacterium *Lyngbya majuscula*. J Nat Prod.

[CR162] Luesch H, Yoshida WY, Moore RE, Paul VJ (2000). Isolation and structure of the cytotoxin lyngbyabellin B and absolute configuration of lyngbyapeptin A from the marine cyanobacterium *Lyngbya majuscula*. J Nat Prod.

[CR163] Luesch H, Yoshida WY, Moore RE, Paul VJ, Mooberry SL (2000). Isolation, structure determination, and biological activity of lyngbyabellin A from the marine cyanobacterium *Lyngbya majuscula*. J Nat Prod.

[CR164] Luesch H, Moore RE, Paul VJ, Mooberry SL, Corbett TH (2001). Isolation of dolastatin 10 from the marine cyanobacterium *Symploca* species VP642 and total stereochemistry and biological evaluation of its analogue symplostatin 1. J Nat Prod.

[CR165] Luesch H, Yoshida WY, Moore RE, Paul VJ, Corbett TH (2001). Total structure determination of apratoxin A, a potent novel cytotoxin from the marine cyanobacterium *Lyngbya majuscula*. J Am Chem Soc.

[CR166] Luesch H, Williams PG, Yoshida WY, Moore RE, Paul VJ (2002). Ulongamides A−F, new β-amino acid-containing cyclodepsipeptides from palauan collections of the marine cyanobacterium *Lyngbya* sp.. J Nat Prod.

[CR167] Luesch H, Yoshida WY, Moore RE, Paul VJ (2002). New apratoxins of marine cyanobacterial origin from guam and palau. Biorg Med Chem.

[CR168] Luesch H, Yoshida WY, Moore RE, Paul VJ (2002). Structurally diverse new alkaloids from Palauan collections of the apratoxin-producing marine cyanobacterium *Lyngbya* sp.. Tetrahedron.

[CR169] Mahyudin NA, Blunt JW, Cole AL, Munro MH (2012). The isolation of a new *S*-methyl benzothioate compound from a marine-derived *Streptomyces* sp.. J Biomed Biotechnol.

[CR170] Malloy KL, Choi H, Fiorilla C, Valeriote FA, Matainaho T, Gerwick WH (2012). Hoiamide D, a marine cyanobacteria-derived inhibitor of p53/MDM2 interaction. Bioorg Med Chem Lett.

[CR171] Maloney KN, Macmillan JB, Kauffman CA, Jensen PR, Dipasquale AG, Rheingold AL, Fenical W (2009). Lodopyridone, a structurally unprecedented alkaloid from a marine actinomycete. Org Lett.

[CR172] Margolin K, Longmate J, Synold TW, Gandara DR, Weber J, Gonzalez R, Johansen MJ, Newman R, Baratta T, Doroshow JH (2001). Dolastatin-10 in metastatic melanoma: a phase II and pharmokinetic trial of the california cancer consortium. Investig New Drugs.

[CR173] Márquez B, Verdier-Pinard P, Hamel E, Gerwick WH (1998). Curacin D, an antimitotic agent from the marine cyanobacterium *Lyngbya majuscula*. Phytochemistry.

[CR174] Marquez BL, Watts KS, Yokochi A, Roberts MA, Verdier-Pinard P, Jimenez JI, Hamel E, Scheuer PJ, Gerwick WH (2002). Structure and absolute stereochemistry of hectochlorin, a potent stimulator of actin assembly. J Nat Prod.

[CR175] Martin J, Da SST, Crespo G, Palomo S, Gonzalez I, Tormo JR, de la Cruz M, Anderson M, Hill RT, Vicente F, Genilloud O, Reyes F (2013). Kocurin, the true structure of PM181104, an anti–methicillin–resistant *Staphylococcus aureus* (MRSA) thiazolyl peptide from the marine–derived bacterium *Kocuria palustris*. Mar Drugs.

[CR176] Martínez V, Davyt D (2013). Total syntheses of bacillamide C and neobacillamide A; revision of their absolute configurations. Tetrahedron Asymmetry.

[CR177] Matsuo Y, Kanoh K, Imagawa H, Adachi K, Nishizawa M, Shizuri Y (2007). Urukthapelstatin A, a novel cytotoxic substance from marine–derived *Mechercharimyces asporophorigenens* YM11–542. J Antibiot.

[CR178] Matsuo Y, Kanoh K, Yamori T, Kasai H, Katsuta A, Adachi K, Shinya K, Shizuri Y (2007). Urukthapelstatin A, a novel cytotoxic substance from marine-derived *Mechercharimyces asporophorigenens* YM11-542. J Antibiot.

[CR179] Matthew S, Ross C, Paul VJ, Luesch H (2008). Pompanopeptins A and B, new cyclic peptides from the marine cyanobacterium *Lyngbya confervoides*. Tetrahedron.

[CR180] Matthew S, Schupp PJ, Luesch H (2008). Apratoxin E, a cytotoxic peptolide from a guamanian collection of the marine cyanobacterium *Lyngbya bouillonii*. J Nat Prod.

[CR181] Matthew S, Paul VJ, Luesch H (2009). Tiglicamides A–C, cyclodepsipeptides from the marine cyanobacterium *Lyngbya confervoides*. Phytochemistry.

[CR182] Matthew S, Salvador LA, Schupp PJ, Paul VJ, Luesch H (2010). Cytotoxic halogenated macrolides and modified peptides from the apratoxin-producing marine cyanobacterium *Lyngbya bouillonii* from Guam. J Nat Prod.

[CR183] McGrath NA, Brichacek M, Njardarson JT (2010). A graphical journey of innovative organic architectures that have improved our lives. J Chem Educ.

[CR184] Meng LH, Li XM, Lv CT, Li CS, Xu GM, Huang CG, Wang BG (2013). Sulfur-containing cytotoxic curvularin macrolides from *Penicillium sumatrense* MA-92, a fungus obtained from the rhizosphere of the mangrove *Lumnitzera racemosa*. J Nat Prod.

[CR185] Meng LH, Li XM, Lv CT, Huang CG, Wang BG (2014). Brocazines A–F, cytotoxic bisthiodiketopiperazine derivatives from *Penicillium brocae* MA-231, an endophytic fungus derived from the marine mangrove plant *Avicennia marina*. J Nat Prod.

[CR186] Meng LH, Zhang P, Li XM, Wang BG (2015). Penicibrocazines A–E, five new sulfide diketopiperazines from the marine–derived endophytic fungus *Penicillium brocae*. Mar Drugs.

[CR187] Meng LH, Wang CY, Mandi A, Li XM, Hu XY, Kassack MU, Kurtan T, Wang BG (2016). Three diketopiperazine alkaloids with spirocyclic skeletons and one bisthiodiketopiperazine derivative from the mangrove-derived endophytic fungus *Penicillium brocae* MA-231. Org Lett.

[CR188] Mevers E, Byrum T, Gerwick WH (2013). Parguerene and precarriebowmide, two classes of lipopeptides from the marine cyanobacterium *Moorea producens*. J Nat Prod.

[CR189] Meyer SW, Mordhorst TF, Lee C, Jensen PR, Fenical W, Kock M (2010). Penilumamide, a novel lumazine peptide isolated from the marine-derived fungus, *Penicillium* sp. CNL-338. Org Biomol Chem.

[CR190] Milligan KE, Marquez BL, Williamson RT, Gerwick WH (2000). Lyngbyabellin B, a toxic and antifungal secondary metabolite from the marine cyanobacterium *Lyngbya majuscula*. J Nat Prod.

[CR191] Mitchell SS, Faulkner DJ, Rubins K, Bushman FD (2000). Dolastatin 3 and two novel cyclic peptides from a palauan collection of *Lyngbya majuscula*. J Nat Prod.

[CR192] Mitova MI, Murphy AC, Lang G, Blunt JW, Cole ALJ, Ellis G, Munro MHG (2008). Evolving trends in the dereplication of natural product extracts. 2. the isolation of chrysaibol, an antibiotic peptaibol from a New Zealand sample of the mycoparasitic fungus *Sepedonium chrysospermum*. J Nat Prod.

[CR193] Molinski TF, Dalisay DS, Lievens SL, Saludes JP (2009). Drug development from marine natural products. Nat Rev Drug Discov.

[CR194] Moore RE, Cheuk C, Yang XQG, Patterson GML, Bonjouklian R, Smitka TA, Mynderse JS, Foster RS, Jones ND, Swartzendruber JK, Deeter JB (1987). Hapalindoles, antibacterial and antimycotic alkaloids from the cyanophyte *Hapalosiphon fontinalis*. J Org Chem.

[CR195] Moosmann P, Ueoka R, Gugger M, Piel J (2018). Aranazoles: extensively chlorinated nonribosomal peptide-polyketide hybrids from the cyanobacterium *Fischerella* sp. PCC 9339. Org Lett.

[CR196] Morgan JB, Liu Y, Coothankandaswamy V, Mahdi F, Jekabsons MB, Gerwick WH, Valeriote FA, Zhou YD, Nagle DG (2015). Kalkitoxin inhibits angiogenesis, disrupts cellular hypoxic signaling, and blocks mitochondrial electron transport in tumor cells. Mar Drugs.

[CR197] Nachtigall J, Schneider K, Bruntner C, Bull AT, Goodfellow M, Zinecker H, Imhoff JF, Nicholson G, Irran E, Sussmuth RD, Fiedler HP (2011). Benzoxacystol, a benzoxazine-type enzyme inhibitor from the deep-sea strain *Streptomyces* sp. NTK 935. J Antibiot (Tokyo).

[CR198] Naidu BN, Sorenson ME, Matiskella JD, Li W, Sausker JB, Zhang Y, Connolly TP, Lam KS, Bronson JJ, Pucci MJ, Yang H, Ueda Y (2006). Synthesis and antibacterial activity of nocathiacin I analogues. Bioorg Med Chem Lett.

[CR199] Nair V, Schuhmann I, Anke H, Kelter G, Fiebig HH, Helmke E, Laatsch H (2016). Marine bacteria, XLVII—psychrotolerant bacteria from extreme antarctic habitats as producers of rare bis- and trisindole alkaloids. Planta Med.

[CR200] Nair V, Kim MC, Golen JA, Rheingold AL, Castro GA, Jensen PR, Fenical W (2020). Verrucosamide, a cytotoxic 1,4-thiazepane-containing thiodepsipeptide from a marine-derived actinomycete. Mar Drugs.

[CR201] Nakamura F, Maejima H, Kawamura M, Arai D, Okino T, Zhao M, Ye T, Lee J, Chang YT, Fusetani N, Nakao Y (2018). Kakeromamide A, a new cyclic pentapeptide inducing astrocyte differentiation isolated from the marine cyanobacterium *Moorea bouillonii*. Bioorg Med Chem Lett.

[CR202] Nakano H, Hara M, Mejiro T, Ando K, Saito Y, Morimoto M (1990) DC1149B, DC1149R, and their manufacture with Trichoderma. JP02218686A

[CR203] Newman DJ, Cragg GM (2020). Natural products as sources of new drugs over the nearly four decades from 01/1981 to 09/2019. J Nat Prod.

[CR204] Newton GL, Jensen PR, Macmillan JB, Fenical W, Fahey RC (2008). An *N*-acyl homolog of mycothiol is produced in marine actinomycetes. Arch Microbiol.

[CR205] Niu S, Liu D, Shao ZZ, Proksch P, Lin WH (2017). Eutypellazines A–M, thiodiketopiperazine-type alkaloids from deep sea derived fungus *Eutypella* sp. MCCC 3A00281. RSC Adv.

[CR206] Niu SW, Liu D, Shao ZZ, Proksch P, Lin WH (2017). Eutypellazines N−S, new thiodiketopiperazines from a deep sea sediment derived fungus *Eutypella* sp. with anti-VRE activities. Tetrahedron Lett.

[CR207] Nogle LM, Gerwick WH (2002). Somocystinamide A, a novel cytotoxic disulfide dimer from a fijian marine cyanobacterial mixed assemblage. Org Lett.

[CR208] Nogle LM, Williamson RT, Gerwick WH (2001). Somamides A and B, two new depsipeptide analogues of dolastatin 13 from a fijian cyanobacterial assemblage of *Lyngbya majuscula* and *Schizothrix* species. J Nat Prod.

[CR209] Ogino J, Moore RE, Patterson GML, Smith CD (1996). Dendroamides, new cyclic hexapeptides from a blue-green alga. multidrug-resistance reversing activity of dendroamide A. J Nat Prod.

[CR210] Ohno O, Terasaki T, Sano T, Hitomi Y, Miyamoto J, Matsuno K (2020). Inhibitory effects of biseokeaniamide A against lipopolysaccharide–induced signal transduction. Bioorg Med Chem Lett.

[CR211] Oliva B, O'Neill A, Wilson JM, O'Hanlon PJ, Chopra I (2001). Antimicrobial properties and mode of action of the pyrrothine holomycin. Antimicrob Agents Chemother.

[CR212] Orjala J, Gerwick WH (1996). Barbamide, a chlorinated metabolite with molluscicidal activity from the caribbean cyanobacterium *Lyngbya majuscula*. J Nat Prod.

[CR213] Ortiz-López F, Alcalde E, Sarmiento-Vizcaíno A, Díaz C, Cautain B, García L, Blanco G, Reyes F (2018). New 3-hydroxyquinaldic acid derivatives from cultures of the marine derived actinomycete *Streptomyces cyaneofuscatus* M-157. Mar Drugs.

[CR214] Pedras MSC, Séguin-Swartz G, Abrams SR (1990). Minor phytotoxins from the blackleg fungus *Phoma lingam*. Phytochemistry.

[CR215] Pereira A, Cao ZY, Murray TF, Gerwick WH (2009). Hoiamide A, a sodium channel activator of unusual architecture from a consortium of two Papua New Guinea cyanobacteria. Chem Biol.

[CR216] Pereira AR, Etzbach L, Engene N, Muller R, Gerwick WH (2011). Molluscicidal metabolites from an assemblage of palmyra atoll cyanobacteria. J Nat Prod.

[CR217] Pereira AR, Kale AJ, Fenley AT, Byrum T, Debonsi HM, Gilson MK, Valeriote FA, Moore BS, Gerwick WH (2012). The carmaphycins: new proteasome inhibitors exhibiting an α, β-epoxyketone warhead from a marine cyanobacterium. ChemBioChem.

[CR218] Perez Baz J, Cañedo LM, Fernández Puentes JL, Silva Elipe MV (1997). Thiocoraline, a novel depsipeptide with antitumor activity produced by a marine *Micromonospora*. II. Physico-chemical properties and structure determination. J Antibiot (Tokyo).

[CR219] Petitbois JG, Casalme LO, Lopez JAV, Alarif WM, Abdel-Lateff A, Al-Lihaibi SS, Yoshimura E, Nogata Y, Umezawa T, Matsuda F, Okino T (2017). Serinolamides and Lyngbyabellins from an *Okeania* sp. cyanobacterium collected from the Red Sea. J Nat Prod.

[CR220] Petkowski JJ, Bains W, Seager S (2018). Natural products containing a nitrogen-sulfur bond. J Nat Prod.

[CR221] Pettit GR, Kamano Y, Brown P, Gust D, Inoue M, Herald CL (1982). Structure of the cyclic peptide dolastatin 3 from *Dolabella auricularia*. J Am Chem Soc.

[CR222] Pettit GR, Kamano Y, Holzapfel CW, Van Zyl WJ, Tuinman AA, Herald CL, Baczynskyj L, Schmidt JM (1987). The structure and synthesis of dolastatin 3. J Am Chem Soc.

[CR223] Philkhana SC, Verma AK, Jachak GR, Hazra B, Basu A, Reddy DS (2017). Identification of new anti-inflammatory agents based on nitrosporeusine natural products of marine origin. Eur J Med Chem.

[CR224] Poncet J (1999). The dolastatins, a family of promising antineoplastic agents. Curr Pharm Des.

[CR225] Prachyawarakorn V, Mahidol C, Sureram S, Sangpetsiripan S, Wiyakrutta S, Ruchirawat S, Kittakoop P (2008). Diketopiperazines and phthalides from a marine derived fungus of the order pleosporales. Planta Med.

[CR226] Prompanya C, Dethoup T, Gales L, Lee M, Pereira JA, Silva AM, Pinto MM, Kijjoa A (2016). New polyketides and new benzoic acid derivatives from the marine sponge-associated fungus *Neosartorya quadricincta* KUFA 0081. Mar Drugs.

[CR227] Raju R, Piggott AM, Khalil Z, Bernhardt PV, Capon RJ (2012). Heronamycin A: a new benzothiazine ansamycin from an Australian marine-derived *Streptomyces* sp.. Tetrahedron Lett.

[CR228] Reddy Penjarla T, Kundarapu M, Syed Mohd B, Bhattacharya A (2017). A straight forward and first total synthesis of Penilumamides B-D. Tetrahedron Lett.

[CR229] Reimer D, Hughes CC (2017). Thiol-based probe for electrophilic natural products reveals that most of the ammosamides are artifacts. J Nat Prod.

[CR230] Ritzau M, Keller M, Wessels P, Stetter KO, Zeeck A (1993) New cyclic polysulfides from hyperthermophilic archaea of the genus *Thermococcus*. Liebigs Ann Chem 91:871–876

[CR231] Rodrigues BSF, Sahm BDB, Jimenez PC, Pinto FCL, Mafezoli J, Mattos MC, Rodrigues-Filho E, Pfenning LH, Abreu LM, Costa-Lotufo LV, Oliveira MCF (2015). Bioprospection of cytotoxic compounds in fungal strains recovered from sediments of the Brazilian Coast. Chem Biodivers.

[CR232] Romero F, Espliego F, Pérez Baz J, García de Quesada T, Grávalos D, de la Calle F, Fernández-Puentes JL (1997). Thiocoraline, a new depsipeptide with antitumor activity produced by a marine *Micromonospora*. I. Taxonomy, fermentation, isolation, and biological activities. J Antibiot (Tokyo).

[CR233] Sarmiento-Vizcaino A, Brana AF, Perez-Victoria I, Martin J, de Pedro N, Cruz M, Diaz C, Vicente F, Acuna JL, Reyes F, Garcia LA, Blanco G (2017). Paulomycin G, a new natural product with cytotoxic activity against tumor cell lines produced by deep-sea sediment derived *Micromonospora matsumotoense* M-412 from the Aviles Canyon in the Cantabrian Sea. Mar Drugs.

[CR234] Sasaki H, Teruya T, Fukazawa H, Suenaga K (2011). Revised structure and structure-activity relationship of bisebromoamide and structure of norbisebromoamide from the marine cyanobacterium *Lyngbya* sp.. Tetrahedron.

[CR235] Sata N, Abinsay H, Yoshida WY, Horgen FD, Sitachitta N, Kelly M, Scheuer PJ (2005). Lehualides A–D, metabolites from a Hawaiian Sponge of the Genus *Plakortis*. J Nat Prod.

[CR236] Scharf DH, Remme N, Habel A, Chankhamjon P, Scherlach K, Heinekamp T, Hortschansky P, Brakhage AA, Hertweck C (2011). A dedicated glutathione *s*-transferase mediates carbon–sulfur bond formation in gliotoxin biosynthesis. J Am Chem Soc.

[CR237] Schwenk S, Ronco C, Oberheide A, Arndt HD (2016). Biomimetic synthesis of urukthapelstatin A by Aza-Wittig Ring contraction. Eur J Org Chem.

[CR238] Seitz T, Fu P, Haut FL, Adam L, Habicht M, Lentz D, MacMillan JB, Christmann M (2016). One-Pot synthesis of 5-hydroxy-4*h*-1,3-thiazin-4-ones: structure revision, synthesis, and nmr shift dependence of thiasporine A. Org Lett.

[CR239] Shanthi J, Senthil A, Gopikrishnan V, Balagurunathan R (2015). Characterization of a potential β-lactamase inhibitory metabolite from a marine *Streptomyces* sp. PM49 active against multidrug-resistant pathogens. Appl Biochem Biotechnol.

[CR240] Shi HY, Xie Y, Hu P, Guo ZQ, Lu YH, Gao Y, Huang CG (2018). Asymmetric synthesis of the C15–C32 fragment of alotamide and determination of the relative stereochemistry. Mar Drugs.

[CR241] Shi ZZ, Miao FP, Fang ST, Yin XL, Ji NY (2018). Sulfurated diketopiperazines from an algicolous isolate of *Trichoderma virens*. Phytochem Lett.

[CR242] Shin J, Fenical W (1987). Isolation of gliovictin from the marine deuteromycete *Asteromyces cruciatus*. Phytochemistry.

[CR243] Shin B, Ahn S, Noh M, Shin J, Oh DC (2015). Suncheonosides A–D, benzothioate glycosides from a marine-derived *Streptomyces* sp.. J Nat Prod.

[CR244] Shiozawa H, Takahashi S (1994). Configurational studies on thiomarinol. J Antibiot (Tokyo).

[CR245] Shiozawa H, Kagasaki T, Kinoshita T, Haruyama H, Domon H, Utsui Y, Kodama K, Takahashi S (1994). Thiomarinol, a new hybrid antimicrobial antibiotic produced by a marine bacterium. Fermentation, isolation, structure, and antimicrobial activity. J Antibiot.

[CR246] Shiozawa H, Kagasaki T, Torikata A, Tanaka N, Fujimoto K, Hata T, Furukawa Y, Takahashi S (1995). Thiomarinols B and C, new antimicrobial antibiotics produced by a marine bacterium. J Antibiot.

[CR247] Shiozawa H, Shimada A, Takahashi S (1997). Thiomarinols D, E, F and G, new hybrid antimicrobial antibiotics produced by a marine bacterium; isolation, structure, and antimicrobial activity. J Antibiot.

[CR248] Sitachitta N, Márquez BL, Thomas Williamson R, Rossi J, Ann Roberts M, Gerwick WH, Nguyen VA, Willis CL (2000). Biosynthetic pathway and origin of the chlorinated methyl group in barbamide and dechlorobarbamide, metabolites from the marine cyanobacterium *Lyngbya majuscula*. Tetrahedron.

[CR249] Smith CJ, Abbanat D, Bernan VS, Maiese WM, Greenstein M, Jompa J, Tahir A, Ireland CM (2000). Novel polyketide metabolites from a species of marine fungi. J Nat Prod.

[CR250] Sobik P, Grunenberg J, Böröczky K, Laatsch H, Wagner-Döbler I, Schulz S (2007). Identification, synthesis, and conformation of tri– and tetrathiacycloalkanes from marine bacteria. J Org Chem.

[CR251] Socha AM, Long RA, Rowley DC (2007). Bacillamides from a hypersaline microbial mat bacterium. J Nat Prod.

[CR252] Son BW, Jensen PR, Kauffman CA, Fenical W (1999). New cytotoxic epidithiodioxopiperazines related to verticillin A from a marine isolate of the fungus *Penicillium*. Nat Prod Lett.

[CR253] Soria-Mercado IE, Pereira A, Cao Z, Murray TF, Gerwick WH (2009). Alotamide A, a novel neuropharmacological agent from the marine cyanobacterium *Lyngbya bouillonii*. Org Lett.

[CR254] Spoof L, Blaszczyk A, Meriluoto J, Ceglowska M, Mazur–Marzec H, (2015). Structures and activity of new anabaenopeptins produced by baltic sea cyanobacteria. Mar Drugs.

[CR255] Stierle AA, Cardellina JH, Singleton FL (1991). Benzothiazoles from a putatitve bacterial symbiont of the marine sponge *Tedania ignis*. Tetrahedron Lett.

[CR256] Sudek S, Haygood MG, Youssef DT, Schmidt EW (2006). Structure of trichamide, a cyclic peptide from the bloom-forming cyanobacterium *Trichodesmium erythraeum*, predicted from the genome sequence. Appl Environ Microbiol.

[CR257] Suhadolnik RJ, Chenoweth RG (1958). Biosynthesis of Gliotoxin. I.1 incorporation of phenylalanine–1– and –2–C14. J Am Chem Soc.

[CR258] Sun Y, Takada K, Takemoto Y, Yoshida M, Nogi Y, Okada S, Matsunaga S (2012). Gliotoxin analogues from a marine-derived fungus, *Penicillium* sp., and their cytotoxic and histone methyltransferase inhibitory activities. J Nat Prod.

[CR259] Sun YL, Bao J, Liu KS, Zhang XY, He F, Wang YF, Nong XH, Qi SH (2013). Cytotoxic dihydrothiophene-condensed chromones from the marine-derived fungus *Penicillium oxalicum*. Planta Med.

[CR260] Sun ZH, Gu JY, Ye W, Wen LX, Lin QB, Li SN, Chen YC, Li HH, Zhang WM (2018). Geospallins A(–)C: new thiodiketopiperazines with inhibitory activity against angiotensin-converting enzyme from a deep-sea-derived fungus *Geosmithia pallida* FS140. Mar Drugs.

[CR261] Supong K, Thawai C, Suwanborirux K, Choowong W, Supothina S, Pittayakhajonwut P (2012). Antimalarial and antitubercular C-glycosylated benz[α]anthraquinones from the marine-derived *Streptomyces* sp. BCC45596. Phytochem Lett.

[CR262] Suzumura KI, Yokoi T, Funatsu M, Nagai K, Suzuki K (2003). YM–266183 and YM–266184, novel thiopeptide antibiotics produced by *Bacillus cereus* isolated from a marine sponge. Part 2. Structure elucidation. J Antibiot.

[CR263] Suzumura KI, Yokoi T, Funatsu M, Nagai K, Suzuki K (2003). YM-266183 and YM-266184, novel thiopeptide antibiotics produced by *Bacillus cereus* isolated from a marine sponge. Part I. Taxonomy, fermentation, isolation, physico–chemical properties and biological properties. J Antibiot.

[CR264] Takahashi C, Numata A, Ito Y, Matsumura E, Araki H, Iwaki H, Kushida K (1994). Leptosins, antitumour metabolites of a fungus isolated from a marine alga. J Chem Soc.

[CR265] Takahashi C, Numata A, Matsumura E, Minoura K, Eto H, Shingu T, Ito T, Hasegawa T (1994). Leptosins I and J, cytotoxic substances produced by a *Leptosphaeria* sp. Physico-chemical properties and structures. J Antibiot (Tokyo).

[CR266] Takahashi C, Minoura K, Yamada T, Numata A, Kushida K, Shingu T, Hagishita S, Nakai H, Sato T, Harada H (1995). Potent cytotoxic metabolites from a *Leptosphaeria* species. Structure determination and conformational analysis. Tetrahedron.

[CR267] Takahashi C, Takai Y, Kimura Y, Numata A, Shigematsu N, Tanaka H (1995). Cytotoxic metabolites from a fungal adherent of a marine alga. Phytochemistry.

[CR268] Takaishi S, Tuchiya N, Sato A, Negishi T, Takamatsu Y, Matsushita Y, Watanabe T, Iijima Y, Haruyama H, Kinoshita T, Tanaka M, Kodama K (1998). B–90063, a novel endothelin converting enzyme inhibitor isolated from a new marine bacterium, *Blastobacter* sp. SANK 71894. J Antibiot (Tokyo).

[CR269] Tamaoki T, Nomoto H, Takahashi I, Kato Y, Morimoto M, Tomita F (1986). Staurosporine, a potent inhibitor of phospholipid Ca++ dependent protein kinase. Biochem Biophys Res Commun.

[CR270] Tan RX, Jensen PR, Williams PG, Fenical W (2004). Isolation and structure assignments of rostratins A−D, cytotoxic disulfides produced by the marine-derived fungus *Exserohilum rostratum*. J Nat Prod.

[CR271] Taori K, Paul VJ, Luesch H (2008). Structure and activity of largazole, a potent antiproliferative agent from the floridian marine cyanobacterium *Symploca* sp.. J Am Chem Soc.

[CR272] Taori K, Paul VJ, Luesch H (2008). Structure and activity of largazole, a potent antiproliferative agent from the floridian marine cyanobacterium *Symploca* sp.. J Am Chem Soc.

[CR273] Tatsuta K, Suzuki Y, Toriumi T, Furuya Y, Hosokawa S (2007). The first total synthesis and structural determination of (+)-BE-52440A. Tetrahedron Lett.

[CR274] Teruya T, Sasaki H, Fukazawa H, Suenaga K (2009). Bisebromoamide, a potent cytotoxic peptide from the marine cyanobacterium *Lyngbya* sp.: isolation, stereostructure, and biological activity. Org Lett.

[CR275] Thesmar P, Baudoin O (2019). Efficient and divergent total synthesis of (−)-Epicoccin G and (−)-Rostratin A enabled by double C(sp(3))-H activation. J Am Chem Soc.

[CR276] Thornburg CC, Thimmaiah M, Shaala LA, Hau AM, Malmo JM, Ishmael JE, Youssef DT, McPhail KL (2011). Cyclic depsipeptides, grassypeptolides D and E and Ibu–epidemethoxylyngbyastatin 3, from a Red Sea *Leptolyngbya* cyanobacterium. J Nat Prod.

[CR277] Thornburg CC, Cowley ES, Sikorska J, Shaala LA, Ishmael JE, Youssef DT, McPhail KL (2013). Apratoxin H and apratoxin A sulfoxide from the Red Sea cyanobacterium *Moorea producens*. J Nat Prod.

[CR278] Tian YQ, Qin XC, Lin XP, Kaliyaperumal K, Zhou XF, Liu J, Ju ZR, Tu ZC, Liu YH (2015). Sydoxanthone C and acremolin B produced by deep-sea-derived fungus *Aspergillus* sp. SCSIO Ind09F01. J Antibiot (Tokyo).

[CR279] Tidgewell K, Engene N, Byrum T, Media J, Doi T, Valeriote FA, Gerwick WH (2010). Evolved diversification of a modular natural product pathway: apratoxins F and G, two cytotoxic cyclic depsipeptides from a Palmyra collection of *Lyngbya bouillonii*. ChemBioChem.

[CR280] Trzoss L, Fukuda T, Costa-Lotufo LV, Jimenez P, La Clair JJ, Fenical W (2014). Seriniquinone, a selective anticancer agent, induces cell death by autophagocytosis, targeting the cancer–protective protein dermcidin. Proc Natl Acad Sci USA.

[CR281] Usami Y, Aoki S, Hara T, Numata A (2002). New dioxopiperazine metabolites from a *Fusarium* species separated from a marine alga. J Antibiot.

[CR282] Usami Y, Yamaguchi J, Numata AJC (2004). Gliocladins A-C and Glioperazine: cytotoxic dioxo- or trioxopiperazine metabolites from a *Gliocladium* sp. separated from a sea hare. Heterocycles.

[CR283] Vaaland IC, Lindbäck E, Sydnes MO (2019). Total synthesis of anithiactins A–C and thiasporine A. Tetrahedron Lett.

[CR284] Wagner M, Abdel-Mageed WM, Ebel R, Bull AT, Goodfellow M, Fiedler HP, Jaspars M (2014). Dermacozines H-J isolated from a deep-sea strain of *Dermacoccus abyssi* from Mariana Trench sediments. J Nat Prod.

[CR285] Wang R, Seyedsayamdost MR (2017). Roseochelin B, an algaecidal natural product synthesized by the *Roseobacter Phaeobacter inhibens* in response to algal sinapic acid. Org Lett.

[CR286] Wang WL, Wang Y, Tao HW, Peng XP, Liu PP, Zhu WM (2009). Cerebrosides of the halotolerant fungus *Alternaria raphani* isolated from a sea salt field. J Nat Prod.

[CR287] Wang FZ, Huang Z, Shi XF, Chen YC, Zhang WM, Tian XP, Li J, Zhang S (2012). Cytotoxic indole diketopiperazines from the deep sea-derived fungus *Acrostalagmus luteoalbus* SCSIO F457. Bioorg Med Chem Lett.

[CR288] Wang JF, Liu PP, Wang Y, Wang H, Li J, Zhuang YB, Zhu WM (2012). Antimicrobial aromatic polyketides from gorgonian-associated fungus, *Penicillium commune* 518. Chin J Chem.

[CR289] Wang Y, Li ZL, Bai J, Zhang LM, Wu X, Zhang L, Pei YH, Jing YK, Hua HM (2012). 2,5–diketopiperazines from the marine-derived fungus *Aspergillus fumigatus* YK-7. Chem Biodivers.

[CR290] Wang Q, Song FH, Xiao X, Huang P, Li L, Monte A, Abdel-Mageed WM, Wang J, Guo H, He WN, Xie F, Dai HQ, Liu MM, Chen CX, Xu H, Liu M, Piggott AM, Liu XT, Capon RJ, Zhang LX (2013). Abyssomicins from the South China Sea deep-sea sediment *Verrucosispora* sp.: natural thioether Michael addition adducts as antitubercular prodrugs. Angew Chem Int Ed.

[CR291] Wang LP, Mei XG, Wang C, Zhu WM (2015). Biomimetic semi-synthesis of fradcarbazole A and its analogues. Tetrahedron.

[CR292] Wang KT, Xu MY, Liu W, Li HJ, Xu J, Yang DP, Lan WJ, Wang LY (2016). Two additional new compounds from the marine-derived fungus *Pseudallescheria ellipsoidea* F42–3. Molecules.

[CR293] Wang B, Tao Y, Liu Q, Liu N, Jin Z, Xu X (2017). Algicidal activity of bacillamide alkaloids and their analogues against marine and freshwater harmful algae. Mar Drugs.

[CR294] Wang DY, Wang Y, Ouyang YF, Fu P, Zhu WM (2019). Cytotoxic *p*-terphenyls from a marine-derived *Nocardiopsis* species. J Nat Prod.

[CR295] Wang NZ, Saidhareddy P, Jiang XF (2020). Construction of sulfur-containing moieties in the total synthesis of natural products. Nat Prod Rep.

[CR296] Wang QY, Zhang KJ, Wang W, Zhang GJ, Zhu TJ, Che Q, Gu QQ, Li DH (2020). Amphiepicoccins A–J: epipolythiodioxopiperazines from the fish-gill-derived fungus *Epicoccum nigrum* HDN17-88. J Nat Prod.

[CR297] Wang WX, Feng HM, Sun CX, Che Q, Zhang GJ, Zhu TJ, Li DH (2020). Thiocladospolides F-J, antibacterial sulfur containing 12-membered macrolides from the mangrove endophytic fungus *Cladosporium oxysporum* HDN13-314. Phytochemistry.

[CR298] Weindling R (1932). *Trichoderma lignorum* as a parasite of other soil fungi. Phytopathology.

[CR299] White JD, Xu Q, Lee CS, Valeriote FA (2004). Total synthesis and biological evaluation of (+)-kalkitoxin, a cytotoxic metabolite of the cyanobacterium *Lyngbya majuscula*. Org Biomol Chem.

[CR300] Williams PG, Yoshida WY, Moore RE, Paul VJ (2002). Isolation and structure determination of obyanamide, a novel cytotoxic cyclic depsipeptide from the marine cyanobacterium *Lyngbya confervoides*. J Nat Prod.

[CR301] Williams PG, Luesch H, Yoshida WY, Moore RE, Paul VJ (2003). Continuing studies on the cyanobacterium *Lyngbya* sp.: isolation and structure determination of 15-norlyngbyapeptin A and lyngbyabellin D. J Nat Prod.

[CR302] Williams PG, Yoshida WY, Moore RE, Paul VJ (2004). Micromide and guamamide: cytotoxic alkaloids from a species of the marine cyanobacterium *Symploca*. J Nat Prod.

[CR303] Winstead JA, Suhadolnik RJ (1960). Biosnthesis of Gliotoxin. II.1,2 further studies on the incorporation of carbon-14 and tritium-labeled precursors. J Am Chem Soc.

[CR304] Wishart DS, Feunang YD, Guo AC, Lo EJ, Marcu A, Grant JR, Sajed T, Johnson D, Li C, Sayeeda Z, Assempour N, Iynkkaran I, Liu Y, Maciejewski A, Gale N, Wilson A, Chin L, Cummings R, Le D, Pon A (2018). DrugBank 5.0: a major update to the DrugBank database for 2018. Nucleic Acids Res.

[CR305] Woo CM, Gholap SL, Herzon SB (2013). Insights into lomaiviticin biosynthesis. isolation and structure elucidation of (−)-homoseongomycin. J Nat Prod.

[CR306] Wu M, Okino T, Nogle LM, Marquez BL, Williamson RT, Sitachitta N, Berman FW, Murray TF, McGough K, Jacobs R, Colsen K, Asano T, Yokokawa F, Shioiri T, Gerwick WH (2000). Structure, synthesis, and biological properties of kalkitoxin, a novel neurotoxin from the marine cyanobacterium *Lyngbya majuscula*. J Am Chem Soc.

[CR307] Wyche TP, Hou YP, Braun D, Cohen HC, Xiong MP, Bugni TS (2011). First natural analogs of the cytotoxic thiodepsipeptide thiocoraline A from a marine *Verrucosispora* sp.. J Org Chem.

[CR308] Wyche TP, Piotrowski JS, Hou Y, Braun D, Deshpande R, McIlwain S, Ong IM, Myers CL, Guzei IA, Westler WM, Andes DR, Bugni TS (2014). Forazoline A: marine-derived polyketide with antifungal in vivo efficacy. Angew Chem Int Ed.

[CR309] Xie ZP, Zhou L, Guo L, Yang XP, Qu GW, Wu CJ, Zhang SM (2016). Grisemycin, a bridged angucyclinone with a methylsulfinyl moiety from a marine-derived *Streptomyces* sp.. Org Lett.

[CR310] Yamada T, Iwamoto C, Yamagaki N, Yamanouchi T, Minoura K, Yamori T, Uehara Y, Andoh T, Umemura K, Numata A (2002). Leptosins M-N1, cytotoxic metabolites from a *Leptosphaeria* species separated from a marine alga. Structure determination and biological activities. Tetrahedron.

[CR311] Yamada T, Iwamoto C, Yamagaki N, Yamanouchi T, Minoura K, Hagishita S, Numata A (2004). Leptosins O-S, cytotoxic metabolites of a strain of *Leptosphaeria* sp. isolated from a marine alga. Heterocycles.

[CR312] Yamada T, Kogure H, Kataoka M, Kikuchi T, Hirano T (2020). Halosmysin A, a novel 14-membered macrodiolide isolated from the marine-algae-derived fungus *Halosphaeriaceae* sp.. Mar Drugs.

[CR313] Yamazaki H, Rotinsulu H, Narita R, Takahashi R, Namikoshi M (2015). Induced production of halogenated epidithiodiketopiperazines by a marine-derived *Trichoderma* cf. *brevicompactum* with Sodium Halides. J Nat Prod.

[CR314] Yamazaki H, Takahashi O, Murakami K, Namikoshi M (2015). Induced production of a new unprecedented epitrithiodiketopiperazine, chlorotrithiobrevamide, by a culture of the marine–derived *Trichoderma* cf. *brevicompactum* with dimethyl sulfoxide. Tetrahedron Lett.

[CR315] Yamazaki H, Rotinsulu H, Takahashi O, Kirikoshi R, Namikoshi M (2016). Induced production of a new dipeptide with a disulfide bridge by long-term fermentation of marine–derived *Trichoderma* cf. *brevicompactum*. Tetrahedron Lett.

[CR316] Yang AG, Si LL, Shi ZP, Tian L, Liu D, Zhou DM, Proksch P, Lin WH (2013). Nitrosporeusines A and B, unprecedented thioester–bearing alkaloids from the Arctic *Streptomyces nitrosporeus*. Org Lett.

[CR317] Ye XW, Chai WY, Lian XY, Zhang ZZ (2017). Novel propanamide analogue and antiproliferative diketopiperazines from mangrove *Streptomyces* sp. Q24. Nat Prod Res.

[CR318] Yi L, Cui CB, Li CW, Peng JX, Gu QQ (2016). Chromosulfine, a novel cyclopentachromone sulfide produced by a marine-derived fungus after introduction of neomycin resistance. RSC Adv.

[CR319] Yin JD, Zhang CJ, Huang JG, Zhang JP, Liu D, Huang J, Proksch P, Lin WH (2018). Violaceimides A–E, sulfur–containing metabolites from a sponge–associated fungus *Aspergillus violaceus*. Tetrahedron Lett.

[CR320] Ying YC, Taori K, Kim H, Hong JY, Luesch H (2008). Total synthesis and molecular target of largazole, a histone deacetylase inhibitor. J Am Chem Soc.

[CR321] Yokokawa F, Shioiri T (2002). Total synthesis of somamide A, an Ahp (3-amino-6-hydroxy-2-piperidone)-containing cyclic depsipeptide. Tetrahedron Lett.

[CR322] Yoo HD, Gerwick WH (1995). Curacins B and C, new antimitotic natural products from the marine cyanobacterium *Lyngbya majuscula*. J Nat Prod.

[CR323] Yu LL, Li ZY, Peng CS, Li ZY, Guo YW (2009). Neobacillamide A, a novel thiazole-containing alkaloid from the marine bacterium *Bacillus vallismortis* C89, associated with South China Sea Sponge Dysidea avara. Helv Chim Acta.

[CR324] Yu GH, Wang YJ, Yu RL, Feng YY, Wang L, Che Q, Gu QQ, Li DH, Li J, Zhu TJ (2018). Chetracins E and F, cytotoxic epipolythiodioxopiperazines from the marine-derived fungus *Acrostalagmus luteoalbus* HDN13-530. RSC Adv.

[CR325] Yu RL, Wang JY, So LY, Harvey PJ, Shi J, Liang JZ, Dou Q, Li X, Yan XY, Huang YH, Xu QL, Kaas Q, Chow HY, Wong KY, Craik DJ, Zhang XH, Jiang T, Wang Y (2020). Enhanced activity against multidrug–resistant bacteria through coapplication of an analogue of tachyplesin i and an inhibitor of the QseC/B signaling pathway. J Med Chem.

[CR326] Yun K, Khong TT, Leutou AS, Kim GD, Hong J, Lee CH, Son BW (2016). Cristazine, a new cytotoxic dioxopiperazine alkaloid from the mudflat-sediment-derived fungus *Chaetomium cristatum*. Chem Pharm Bull (Tokyo).

[CR327] Yurchenko AN, Smetanina OF, Ivanets EV, Kalinovsky AI, Khudyakova YV, Kirichuk NN, Popov RS, Bokemeyer C, von Amsberg G, Chingizova E, Afiyatullov SS, Dyshlovoy SA (2016). Pretrichodermamides D–F from a marine algicolous fungus *Penicillium* sp. KMM 4672. Mar Drugs.

[CR328] Yurchenko AN, Berdyshev DV, Smetanina OF, Ivanets EV, Zhuravleva OI, Rasin AB, Khudyakova YV, Popov RS, Dyshlovoy SA, von Amsberg G, Afiyatullov SS (2019). Citriperazines A-D produced by a marine algae–derived fungus *Penicillium* sp. KMM 4672. Nat Prod Res.

[CR329] Zhang W, Ma ZH, Mei D, Li CX, Zhang XL, Li YX (2006). Total synthesis and reassignment of stereochemistry of obyanamide. Tetrahedron.

[CR330] Zhang N, Chen YL, Jiang RX, Li EW, Chen XL, Xi ZJ, Guo YL, Liu XZ, Zhou YG, Che YS, Jiang XJ (2014). PARP and RIP 1 are required for autophagy induced by 11'-deoxyverticillin A, which precedes caspase-dependent apoptosis. Autophagy.

[CR331] Zhang C, Naman CB, Engene N, Gerwick WH (2017). Laucysteinamide A, a hybrid PKS/NRPS metabolite from a Saipan Cyanobacterium, cf. *Caldora penicillata*. Mar Drugs.

[CR332] Zhang XF, Chen L, Chai WY, Lian XY, Zhang ZZ (2017). A unique indolizinium alkaloid streptopertusacin A and bioactive bafilomycins from marine-derived *Streptomyces* sp. HZP–2216E. Phytochemistry.

[CR333] Zhang DS, Jiang YJ, Li JQ, Ding WJ, Chen Z, Ma ZJ (2018). Thioquinomycins A–D, novel naphthothiophenediones from the marine-derived *Streptomyces* sp. SS17F. Tetrahedron.

[CR334] Zhang D, Shu CY, Lian XY, Zhang ZZ (2018). New antibacterial bagremycins F and G from the marine-derived *Streptomyces* sp. ZZ745. Mar Drugs.

[CR335] Zhang FZ, Li XM, Yang SQ, Meng LH, Wang BG (2019). Thiocladospolides A-D, 12-membered macrolides from the mangrove–derived endophytic fungus *Cladosporium cladosporioides* MA–299 and structure revision of pandangolide 3. J Nat Prod.

[CR336] Zhang SW, Xie Q, Sun CL, Tian XP, Gui C, Qin XJ, Zhang H, Ju JH (2019). Cytotoxic kendomycins containing the carbacylic ansa scaffold from the marine-derived *Verrucosispora* sp. SCSIO 07399. J Nat Prod.

[CR337] Zhang FZ, Li XM, Meng LH, Wang BG (2020). Cladocladosin A, an unusual macrolide with bicyclo 5/9 ring system, and two thiomacrolides from the marine mangrove-derived endophytic fungus, *Cladosporium cladosporioides* MA-299. Bioorg Chem.

[CR338] Zhao WY, Zhu TJ, Han XX, Fan GT, Liu HB, Zhu WM, Gu QQ (2009). A new gliotoxin analogue from a marine-derived fungus *Aspergillus fumigatus* Fres. Nat Prod Res.

[CR339] Zhen X, Gong T, Liu F, Zhang PC, Zhou WQ, Li Y, Zhu P (2015). A new analogue of echinomycin and a new cyclic dipeptide from a marine-derived *Streptomyces* sp. LS298. Mar Drugs.

[CR340] Zhen F, Sun ZH, Zhong L, Chen YC, Liu HX, Li HH, Zhang WM (2016). Dichotocejpins A–C: new diketopiperazines from a deep-sea-derived fungus *Dichotomomyces cejpii* FS110. Mar Drugs.

[CR341] Zhou X, Huang HB, Chen YC, Tan JH, Song YX, Zou JH, Tian XP, Hua Y, Ju JH (2012). Marthiapeptide A, an anti-infective and cytotoxic polythiazole cyclopeptide from a 60 L scale fermentation of the deep sea-derived *Marinactinospora thermotolerans* SCSIO 00652. J Nat Prod.

[CR342] Zhou B, Ji YY, Zhang HJ, Shen L (2019) Gephyyamycin and cysrabelomycin, two new angucyclinone derivatives from the *Streptomyces* sp. HN–A124. Nat Prod Res 1–6 10.1080/14786419.2019.1660336 34190022

[CR343] Zhu ML, Zhang XM, Feng HM, Dai JJ, Li J, Che Q, Gu QQ, Zhu TJ, Li DH (2017). Penicisulfuranols A–F, alkaloids from the mangrove endophytic fungus *Penicillium janthinellum* HDN13–309. J Nat Prod.

[CR344] Zhu ML, Zhang XW, Huang XN, Wang HT, Anjum K, Gu QQ, Zhu TJ, Zhang GJ, Li DH (2020). Irregularly bridged epipolythiodioxopiperazines and related analogues: sources, structures, and biological activities. J Nat Prod.

[CR345] Zhu ML, Yang Z, Wang HT, Gan Q, Zhang GJ, Che Q, Zhu TJ, Gu QQ, Han BN, Li DH (2020). Penispirozines A-H, three classes of dioxopiperazine alkaloids with spirocyclic skeletons isolated from the mangrove-derived *Penicillium janthinellum*. J Nat Prod.

