# Supplementary material for: The intriguing chemistry and biology of sulfur-containing natural products from marine microorganisms (1987–2020)
Source: Mar Life Sci Technol. 2021 May 19;3(4):488–518. doi: 10.1007/s42995-021-00101-2 (PMC10077240; doi:10.1007/s42995-021-00101-2)
Supplement: Supplementary file 1 — Supplementary file1 (DOCX 3276 KB) [file 42995_2021_101_MOESM1_ESM.docx]

**Supporting Information**

**The Intriguing Chemistry and Biology of Sulfur-Containing Natural Products from Marine Microorganisms (1987–2020)**

Yang Hai,^1,2^ **·** Mei-Yan Wei,^1,3^ **·** Chang-Yun Wang,^1,2^ **·** Yu-Cheng Gu,^4^ **·** Chang-Lun Shao^1,2,*^

1 Key Laboratory of Marine Drugs, The Ministry of Education of China, School of Medicine and Pharmacy, Ocean University of China, Qingdao 266003, China

2 Laboratory for Marine Drugs and Bioproducts, Qingdao National Laboratory for Marine Science and Technology, Qingdao 266200, China

3 College of Food Science and Engineering, Ocean University of China, Qingdao 266003, China

4 Syngenta Jealott’s Hill International Research Centre, Bracknell, Berkshire, RG42 6EY, United Kingdom

Correspondence: shaochanglun@163.com (Chang-Lun Shao)

、

**List of Supporting Information**

**Supplementary Fig. S1** The chemical structures of compounds **1**–**16**

**Supplementary Fig. S2** The chemical structures of compounds **17**–**33**

**Supplementary Fig. S3** The chemical structures of compounds **34**–**40**

**Supplementary Fig. S4** The chemical structures of compounds **41**–**61**

**Supplementary Fig. S5** The chemical structures of compounds **62**–**101**

**Supplementary Fig. S6** The chemical structures of compounds **102–113**

**Supplementary Fig. S7** The chemical structures of compounds **114–125**

**Supplementary Fig. S8** The chemical structures of compounds **126**–**140**

**Supplementary Fig. S9** The chemical structures of compounds **141**–**146**

**Supplementary Fig. S10** The chemical structures of compounds **147**–**174**

**Supplementary Fig. S11** The chemical structures of compounds **175**–**191**

**Supplementary Fig. S12** The chemical structures of compounds **192**–2**10**

**Supplementary Fig. S13** The chemical structures of compounds **211–221**

**Supplementary Fig. S14** The chemical structures of compounds **222–232**

**Supplementary Fig. S15** The chemical structures of compounds **233**–**243**

**Supplementary Fig. S16** The chemical structures of compounds **244**–**261**

**Supplementary Fig. S17** The chemical structures of compounds **262**–**268**

**Supplementary Fig. S18** The chemical structures of compounds **269**–**290**

**Supplementary Fig. S19** The chemical structures of compounds **291**–**299**

**Supplementary Fig. S20** The chemical structures of compounds **300–314**

**Supplementary Fig. S21** The chemical structures of compounds **315–325**

**Supplementary Fig. S22** The chemical structures of compounds **326–343**

**Supplementary Fig. S23** Example: lyngbyabellins methanolysis and a regioselective ester cleavage at the C–24 or C–16 positions

**Supplementary Fig. S24** The chemical structures of compounds **344**–**366**

**Supplementary Fig. S25** The chemical structures of compounds **367**–**380**

**Supplementary Fig. S26** The chemical structures of compounds **381**–**397**

**Supplementary Fig. S27** The chemical structures of compounds **398**–**426**

**Supplementary Fig. S28** The chemical structures of compounds **427**–**434**

**Supplementary Fig. S29** The chemical structures of compounds **435**–**444**

**Supplementary Fig. S30** The chemical structures of compounds **445**–**454**

**Supplementary Fig. S31** The chemical structures of compounds **455**–**460**

**Supplementary Fig. S32** The chemical structures of compounds **461**–**472**

**Supplementary Fig. S33** The chemical structures of compounds **473**–**481**

**Supplementary Fig. S34** The chemical structures of compounds **482**–**484**

**Supplementary Fig. S35** Proposed biosynthesis pathway of some ETPs by Guo and co-workers (some changes)

**Supplementary Fig. S36** Total synthesis of (+)-gliocladin A by Overman and co-workers (key steps)

**Supplementary Fig. S37** Total synthesis of 11,11’-dideoxyverticillin A by Movassaghi and co-workers (key steps)

**Supplementary Fig. S38** Total synthesis of luteoalbusins A and B by Movassaghi and co-workers (key steps)

**Supplementary Fig. S1** The chemical structures of compounds **1**–**16**

**Supplementary Fig. S2** The chemical structures of compounds **17**–**33**

**Supplementary Fig. S3** The chemical structures of compounds **34**–**40**

**Supplementary Fig. S4** The chemical structures of compounds **41**–**61**

**Supplementary Fig. S5** The chemical structures of compounds **62**–**101**

**Supplementary Fig. S6** The chemical structures of compounds **102**–**113**

**Supplementary Fig. S7** The chemical structures of compounds **114**–**125**

**Supplementary Fig. S8** The chemical structures of compounds **126**–**140**

**Supplementary Fig. S9** The chemical structures of compounds **141**–**146**

**Supplementary Fig. S10** The chemical structures of compounds **147**–**174**

**Supplementary Fig. S11** The chemical structures of compounds **175**–**191**

**Supplementary Fig. S12** The chemical structures of compounds **192**–**210**

**Supplementary Fig. S13** The chemical structures of compounds **211**–**221**

**Supplementary Fig. S14** The chemical structures of compounds **222**–**232**

**Supplementary Fig. S15** The chemical structures of compounds **233**–**243**

**Supplementary Fig. S16** The chemical structures of compounds **244**–**261**

**Supplementary Fig. S17** The chemical structures of compounds **262**–**268**

**Supplementary Fig. S18** The chemical structures of compounds **269**–**290**

**Supplementary Fig. S19** The chemical structures of compounds **291**–**299**

**Supplementary Fig. S20** The chemical structures of compounds **300**–**314**

**Supplementary Fig. S21** The chemical structures of compounds **315**–**325**

**Supplementary Fig. S22** The chemical structures of compounds **326**–**343**

**Supplementary Fig. S23** Example: lyngbyabellins methanolysis and a regioselective ester cleavage at the C–24 or C–16 positions

**Supplementary Fig. S24** The chemical structures of compounds **344**–**366**

**Supplementary Fig. S25** The chemical structures of compounds **367**–**380**

**Supplementary Fig. S26** The chemical structures of compounds **381**–**397** and **394a–394f**

**Supplementary Fig. S27** The chemical structures of compounds **398**–**426**

**Supplementary Fig. S28** The chemical structures of compounds **427**–**434**

**Supplementary Fig. S29** The chemical structures of compounds **435**–**444**

**Supplementary Fig. S30** The chemical structures of compounds **445**–**454**

**Supplementary Fig. S31** The chemical structures of compounds **455**–**460**

**Supplementary Fig. S32** The chemical structures of compounds **461**–**472**

**Supplementary Fig. S33** The chemical structures of compounds **473**–**481**

**Supplementary Fig. S34** The chemical structures of compounds **482**–**484**

**Supplementary Fig. S35** Proposed biosynthesis pathway of some ETPs by Guo and co-workers (some changes)

**Supplementary Fig. S36** Total synthesis of (+)-gliocladin A by Overman and co-workers (key steps)

**Supplementary Fig. S37** Total synthesis of 11,11’-dideoxyverticillin A by Movassaghi and co-workers (key steps)

**Supplementary Fig. S38** Total synthesis of luteoalbusins A and B by Movassaghi and co-workers (key steps)
